# The global, regional and national burden of peptic ulcer disease from 1990 to 2019: a population-based study

**DOI:** 10.1186/s12876-022-02130-2

**Published:** 2022-02-10

**Authors:** Xin Xie, Kaijie Ren, Zhangjian Zhou, Chengxue Dang, Hao Zhang

**Affiliations:** 1 Department of Surgical Oncology, The First Affiliated Hospital of Xi’an Jiaotong University, Xi’an, 710061 Shaanxi China; 2 Department of Nuclear Medicine, The First Affiliated Hospital of Xi’an Jiaotong University, Xi’an, 710061 Shaanxi China; 3 Department of Oncology, The Second Affiliated Hospital of Xi’an Jiaotong University, Xi’an, 710004 Shaanxi China; 4 Clinical Medicine and Cancer Research Center of Shaanxi Province, Xi’an, 710061 Shaanxi China

**Keywords:** Peptic ulcer disease, Disease burden, Sociodemographic characteristics, Global Burden of Disease, Injuries and Risk Factors Study

## Abstract

**Background:**

Peptic ulcer disease (PUD) is a common digestive disorder, of which the prevalence decreased in the past few decades. However, the decreasing tendency has plateaued in recent years due to changes in risk factors associated with the etiology of PUD, such as non-steroidal anti-inflammatory drug use. In this study, we investigated the epidemiological and the sociodemographic characteristics of PUD in 204 countries and territories from 1990 to 2019 based on data from the Global Burden of Disease, Injuries and Risk Factors (GBD) Study.

**Methods:**

Demographic characteristics and annual prevalence, incidence, mortality, disability-adjusted life years (DALYs) and age-standardized death rate (ASR) data associated with PUD were obtained and analyzed. According to the sociodemographic index (SDI), the numbers of patients, ASRs, estimated annual percentage changes and geographical distributions were assessed with a generalized linear model and presented in world maps. All evaluations of numbers and rates were calculated per 100,000 population with 95% uncertainty intervals (UIs).

**Results:**

In 2019, the global prevalence of PUD was approximately 8.09 [95% UI 6.79–9.58] million, representing a 25.82% increase from 1990. The age-standardized prevalence rate was 99.40 (83.86–117.55) per 100,000 population in 2019, representing a decrease of 143.37 (120.54–170.25) per 100,000 population from 1990. The age-standardized DALY rate in 2019 was decreased by 60.64% [74.40 (68.96–81.95) per 100,000 population] compared to that in 1990. In both sexes, the numbers and ASRs of the prevalence, incidence, deaths and DALYs were higher in males than in females over 29 years. Regionally, South Asia had the highest age-standardized prevalence rate [156.62 (130.58–187.05) per 100,000 population] in 2019. A low age-standardized death rate was found in the high-income super-region. Among nations, Kiribati had the highest age-standardized prevalence rate [330.32 (286.98–379.81) per 100,000 population]. Regarding socioeconomic status, positive associations between the age-standardized prevalence, incidence, death rate, DALYs and SDI were observed globally in 2019.

**Conclusions:**

Morbidity and mortality due to PUD decreased significantly from 1990 to 2019, while a gradual upward inclination has been observed in recent 15 years, which might be associated with changes in risk factors for PUD. Attention and efforts by healthcare administrators and society are needed for PUD prevention and control.

**Supplementary Information:**

The online version contains supplementary material available at 10.1186/s12876-022-02130-2.

## Background

Peptic ulcer disease (PUD), a common disorder of the digestive system, is defined as digestive tract injury that results in a mucosal break greater than 3–5 mm, with a visible depth reaching the submucosa [1, 2]. Mainly occurring in the stomach and proximal duodenum, PUD accounts for an estimated lifetime prevalence of 5–10% and an annual incidence of 0.1–0.3% in the general population in Western countries [2, 3]. Due to nonspecific symptoms, PUD assessment and treatment requires clinical caution due to severe complications such as bleeding, perforation, penetration into adjacent organs and gastrointestinal obstruction, all of which could require acute endoscopic or surgical treatment [1, 4, 5].

Similar to several digestive disorders, the prevalence of PUD initially increased and then subsequently decreased. Jennings et al*.* analyzed PUD epidemiological data spanning 150 years and found that the incidence of and mortality due to PUD increased markedly during the nineteenth century and then decreased steadily due to improvements in environmental hygiene and medical therapeutic strategies [6]. During the first 50 years of the twentieth century in the United States, PUD affected approximately 10% of the adult population [7]. Several studies which were conducted in the past 20–30 years indicated a sharp decreasing tendency in the PUD prevalence, PUD-related hospital admissions and PUD-associated mortality due to new anti-PUD therapies application, such as *Helicobacter pylori* (*H. pylori*) eradication and proton-pump inhibitors (PPIs) using [8–11]. However, the widespread use of nonsteroidal anti-inflammatory drugs (NSAIDs), histamine 2 receptor antagonists, and selective serotonin reuptake inhibitors, as well as increased physiological stress, have been reported as risk factors and have changed the landscape of PUD in recent years [12, 13]. The details of the epidemiological changes caused by these relatively new risk factors are still controversial.

In this study, we analyzed PUD burdens in 204 countries or territories from 1990 to 2019 based on data from the Global Burden of Disease, Injuries and Risk Factors (GBD) Study, which is updated in 2020 and contains epidemiological and socioeconomic data of 354 diseases globally, allowing evaluations of the burdens, distributions and trends of PUD in different regions. Our study aims to investigate the current landscape and changes in the epidemiological characteristics of PUD to support healthcare-associated policy makers in developing improved PUD prevention strategies.

## Methods

### Data acquisition

The GBD study provides comprehensive epidemiological estimates of the prevalence of, incidence of, disability-adjusted life years (DALYs) due to, and mortality associated with diseases and injuries across specific groups of countries and territories by sex, age and year. Annual (inclusive dates: Jan 1st, 1990 to Dec 31st, 2019) prevalence rates, incidence rates, DALYs and deaths and the corresponding age-standardized rates (ASRs) were extracted from the Global Health Data Exchange (GHDx) database (http://ghdx.healthdata.org/). These data were socioecologically classified into seven GBD super-regions, 21 GBD regions and 204 countries/territories by sociodemographic index (SDI) quintiles, which is a composite indicator of lag-distributed income per capita, ranging from 0 (less developed) to 1 (most developed). SDI value reflects the degree of social development and correlates with total fertility, per capita income, and average years of education [14]. All countries and territories were classified into five quintiles based on the SDI (http://ghdx.healthdata.org/record/ihme-data/gbd-2019-sociodemographic-index-sdi-1950-2019). Besides, super-regions and regions in GBD database are groups of countries rather than geological concepts for analysis (http://www.healthdata.org/sites/default/files/files/Data_viz/GBD_2017_Tools_Overview.pdf).

### Statistical analyses

ASRs and numbers were analyzed to compare PUD prevalence and mortality trends among different cohorts. DALYs refer to the years lived with disability and years of life lost [15]. Estimated annual percentage changes (EAPCs) indicate ASR trends during a defined period. The specific EAPC was calculated using a generalized linear model (GLM) considering a Gaussian distribution for the ASR. Under the assumption of linearity on the log scale, which is equivalent to a constant change assumption, the EAPC was calculated. An EAPC estimation greater than zero indicates an increasing ASR trend, while an estimation less than zero indicates a decreasing ASR trend. If the 95% confidence interval (CI) of the EAPC crossed zero, the change in ASR was not obvious over time.

World maps and graphs were generated to display the distribution and change trends in global, regional, and national disease burdens attributable to PUD. Uncertainty was incorporated by sampling 1,000 draws combining uncertainty from a number of sources, including input data, corrections of measurement errors and estimates of residual nonsampling errors. The 2.5th and 97.5th centiles of the ordered draws were defined as uncertainty intervals (UIs). All calculations and figures were performed and made in EXCEL 2019 (Microsoft Corporation) and R software (version 4.0.0) with the “Rcan”, “ggplot2” and other packages.

## Results

### Global burden and demographic profiles of PUD

Our study indicated that there were approximately 8.09 million (95% UI 6.79 to 9.58 million) prevalent cases of PUD in 2019, which represented an increase of 25.82% from 1990 [6.43 million (95% UI 5.41 to 7.63 million)]. Moreover, the age-standardized prevalence rate in 2019 was 99.40 per 100,000 (95% UI 83.86 to 117.55 per 100,000) population, which represented a decrease from 1990 [143.37 per 100,000 (95% UI 120.54 to 170.25 per 100,000) population](Additional file 1: Table S1). Between 1990 and 2019, the number of incident cases of PUD increased from 2.82 million (95% UI 2.36 to 3.30 million) to more than 3.59 million (95% UI 3.03 to 4.22), representing an increase of 27.3% in the global incident cases of PUD. However, the global age-standardized incidence rate of PUD showed a decreasing trend, at 63.84 (95% UI 54.09 to 75.54) per 100,000 population in 1990 and 44.26 (95% UI 37.32 to 51.87) per 100,000 population in 2019 (Additional file 1: Table S2). At the global level, nearly 6.03 (95% UI 5.59 to 6.64) million DALYs were attributable to PUD, with an age-standardized rate of 74.40 (95% UI 68.96 to 81.95) DALYs per 100,000 population in 2019. The age-standardized rate of DALYs decreased by 60.64% from 1990. Similar trends were also found in PUD-related deaths (Additional file 1 : Tables S3, S4).

**Fig. 1:**
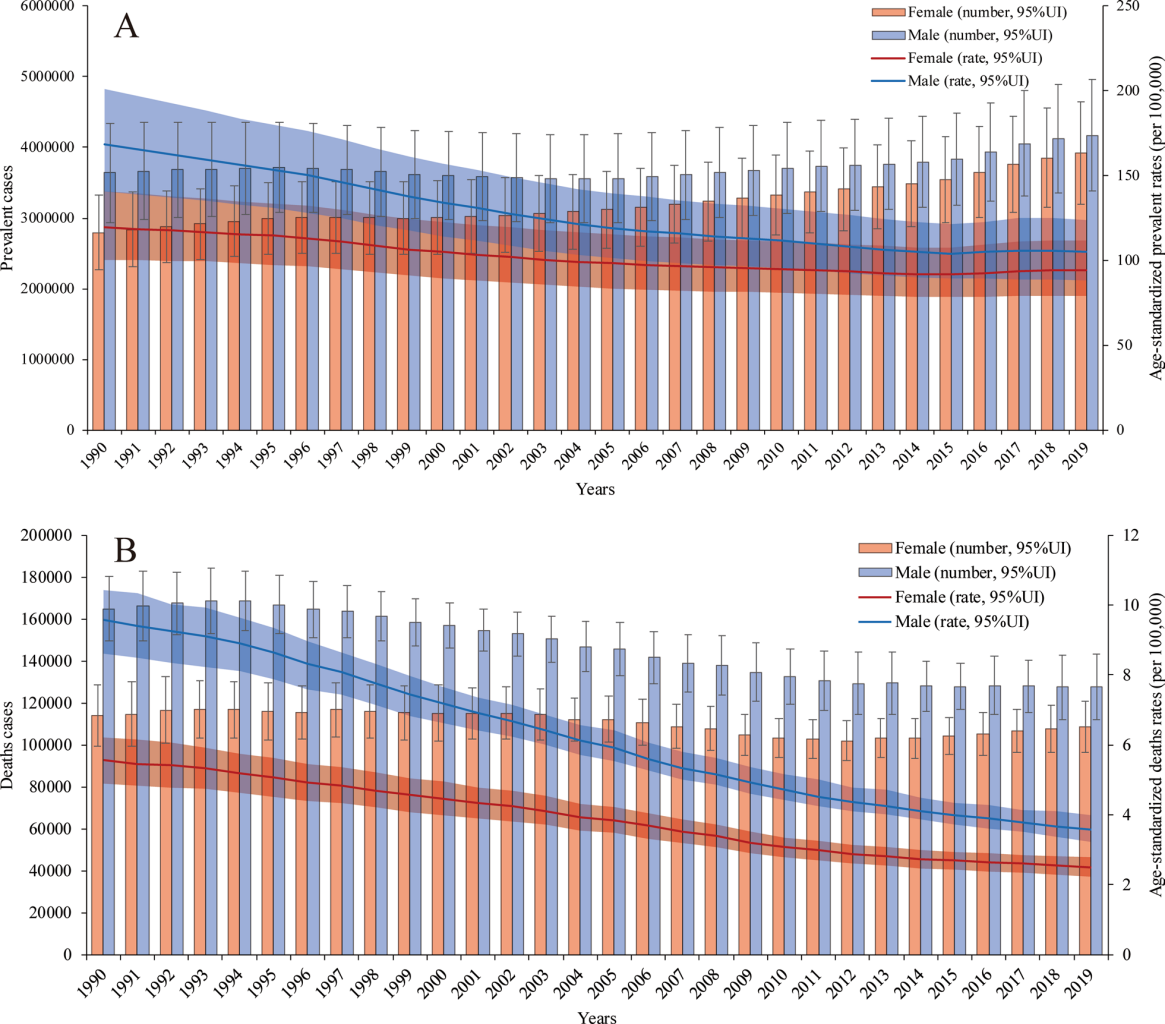
Prevalence rates and deaths with age-standardized rate changes in all years from 1990 to 2019. **a** The numbers of prevalent cases and age-standardized prevalence rates in males and females. **b** The numbers of deaths and age-standardized death rates in males and females

Both the number of prevalent cases and age-standardized prevalence rate were higher in males than in females in all years from 1990 to 2019. However, the difference between the two groups decreased, mainly because the number of prevalent cases and age-standardized prevalence rate in males decreased faster than those in females. Overall, in 2019, 3.92 (95% UI 3.29 to 4.64) million prevalent cases occurred in females, whereas 4.17 (95% UI 3.49 to 4.97) million prevalent cases occurred in males. The proportion of prevalent cases between males and females was 1:0.94. The age-standardized prevalence rate was 94.23 (95% UI 79.10 to 111.93) per 100,000 population in females and 104.98 (95% UI 88.26 to 124.10) per 100,000 population in males in 2019 (Fig. 1a).

From Jan.1st, 1990 to Dec 31st, 2019, the number of PUD-related deaths has shown a gradual, fluctuating decreasing trend in females and a relatively significant decreasing trend in males. Moreover, the age-standardized death rates in both groups showed downward trends. Among males, there were 127,522.08 (95% UI 115,260.65 to 143,079.71) PUD-related deaths and 3.57 (95% UI 3.23 to 4.00) per 100,000 population PUD-related age-standardized deaths in 2019, whereas there were 164,933.87 (95% UI 146,881.12 to 180,422.89) deaths and 9.58 (95% UI 8.62 to 10.43) age-standardized deaths in 1990. Among females, there were 108,617.41 (95% UI 96,020.68 to 120,954.17) PUD-related deaths and 2.50 (95% UI 2.21 to 2.79) per 100,000 population PUD-related age-standardized deaths in 2019, whereas there were 114,044.63 (95% UI 99,995.18 to 128,749.67) death and 5.56 (95% UI 4.91 to 6.22) age-standardized deaths in 1990. The number of PUD-related deaths was lowest in 2012 [102,041.21 (95% UI 92,732.31 to 111,554.31)]. This may be related to a variety of factors, such as the age distributions of the different sexes and the proportions of aging populations around the world (Fig. 1b). The patterns of incidence (Additional file 1: Figure S1) and DALYs (Additional file 1: Figure S2) by sex and year were relatively similar to those of prevalence and death, respectively.

**Fig. 2:**
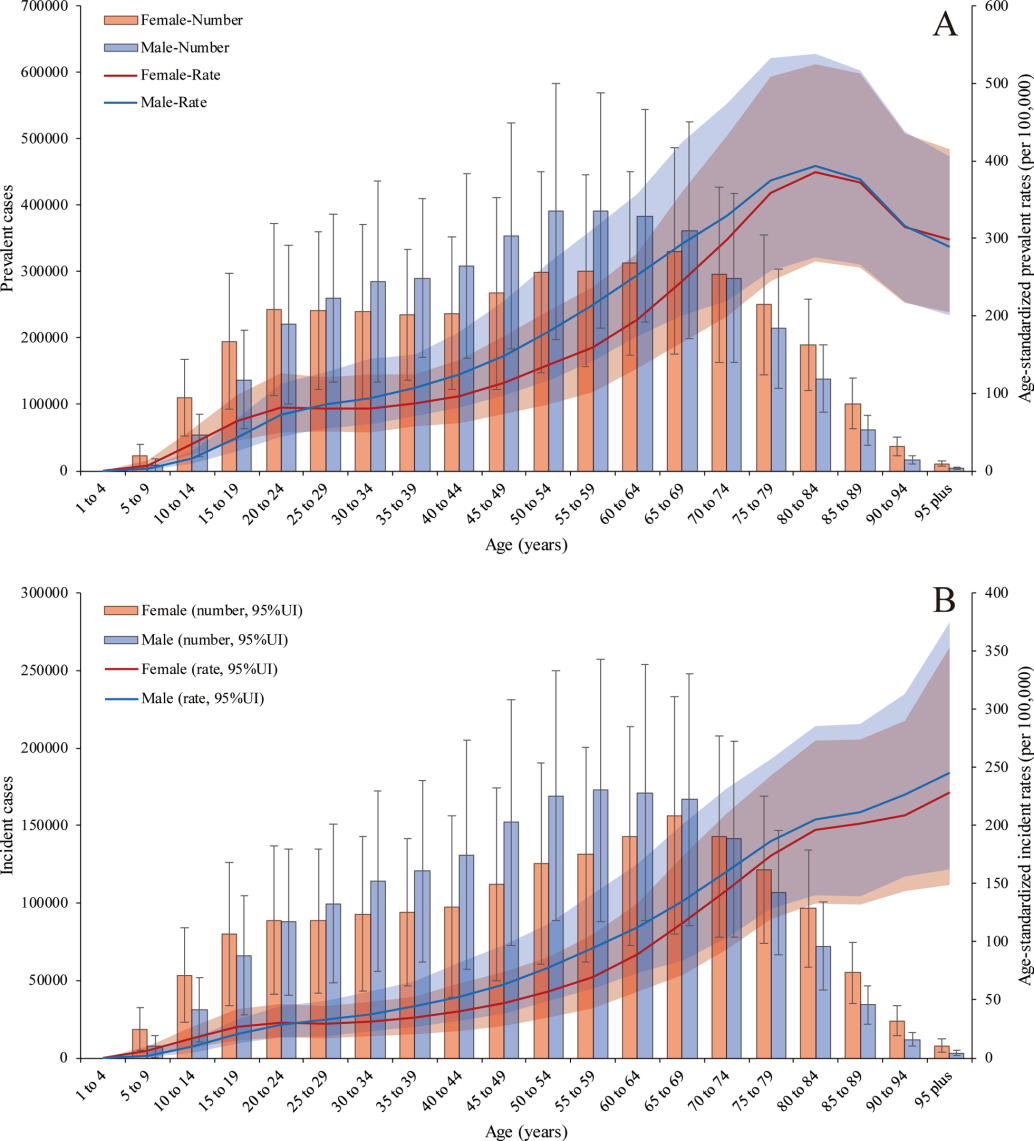
Prevalent and incident cases with age-standardized rate changes in 2019. **a** The numbers of prevalent cases and age-standardized prevalence rates in males and females. **b** The numbers of incident cases and age-standardized death rates in males and females

The global prevalence of PUD was higher in females than in males on both ends of the age spectrum (more than 70 and less than 24 years old). The PUD prevalence rates peaked in 65- to 69-year-old females [330,974.81 (95% UI 223,943.66 to 485,784.86)] and 55- to 59-years-old males [391,973.56 (95% UI 259,447.97 to 569,117.47)] in 2019. In addition, the age-standardized prevalence rates increased with age, peaking at 80–84 years in both males [393.04 (95% UI 275.22 to 537.73)] and females [385.16 (95% UI 270.52 to 525.41)] and then decreased until patients reached the oldest age group in 2019. Age-standardized prevalence rates were also higher in females than in males at both ends of the age spectrum (more than 85 and less than 24 years old) (Fig. 2a). However, the age-standardized incidence rates were higher in males than in females and increased with age, peaking in the more than 95-year-old group in both males and females in 2019 (Fig. 2b). For the age-standardized death rates (Additional file 1: Figure S3), there was a sharp increase in those aged more than 70 years, and the trend in males increased more than that in females; there was a similar trend in DALYs (Additional file 1: Figure S4). PUD-related deaths peaked in 80- to 84-year old female patients; at this point, the number of deaths in female patients exceeded that in male patients, which peaked in 75- to 79-year-old males.

### Regional burden of PUD

For 30 years, the age-standardized prevalence rate in South Asia [156.62 (95% UI 130.58 to 187.05) in 2019] was highest among all the GBD super-regions. However, it showed a sharp decreasing trend. The super-regions with lowest age-standardized prevalence rates were Latin America and the Caribbean [41.77 (95% UI 35.53 to 49.29) in 2019] (Additional file 1: Figure S5). Moreover, the age-standardized incidence rate trends of the 7 super-regions were similar to the prevalence rate trends from 1990 to 2019 (Additional file 1: Figure S6). The high-income super-region had the lowest age-standardized death rate from 1990 to 2019 [1.08 (95% UI 0.96 to 1.19) in 2019]. South Asia presented the largest decreasing trend in the age-standardized death rate during the study period. The percent change from 1990 to 2019 was 66.92% (95% UI 59.25% to 73.22%). Central Europe, Eastern Europe, and Central Asia experienced a fluctuating but gradually decreasing trend (Additional file 1: Figure S7, S8). Among the 21 GBD regions, the prevalence (Additional file 1: Figure S9) and incidence cases (Additional file 1: Figure S10) were highest in South Asia [2.52 (95% UI 2.09 to 3.01) million for prevalence in 2019] and East Asia [1.49 (95% UI 1.22 to 1.82) million for prevalence in 2019], with increasing trends. Although the numbers of PUD-related DALYs (Additional file 1: Figure S11) and deaths (Additional file 1: Figure S12) in South Asia and East Asia accounted for the largest proportions, the number of DALYs showed a decreasing trend. The number of deaths in East Asia showed a slight but fluctuating decrease.

In 2019, the age-standardized prevalence rates in males were higher than those in females in 16 GBD regions, with the exception of Western sub-Saharan Africa, South Asia, North Africa and the Middle East, High-income North America and Central sub-Saharan Africa. The region with the greatest difference in prevalence between males and females was High-income Asia Pacific (male:female, 2.35:1), followed by Central Asia (male:female, 2.13:1)(Additional file 1: Figure S13). The age-standardized incidence rate showed almost the same trend (Additional file 1: Figure S14). The age-standardized DALY rate was slightly higher in females than in males in only South Asia; in the rest of the GBD regions, males had a higher DALY rate than females. The region with the greatest difference in DALYs between males and females was Eastern Europe (male:female, 2.97:1), followed by Central Asia (male:female, 2.46:1)(Additional file 1: Figure S15). The trend of death was similar to that of DALYs in 2019 (Additional file 1: Figure S16). From 1990 to 2019, the age-standardized prevalence rate in males in all GBD regions decreased to varying degrees, but among females, four regions showed variable increases: Eastern Europe, Southern sub-Saharan Africa, Western sub-Saharan Africa and Central sub-Saharan Africa. The age-standardized death rates decreased among all 21 GBD regions, except in females in Eastern Europe and Central Asia. After estimating the EAPCs in the age-standardized prevalence and DALY rates, only females in Eastern Europe showed a consistent increasing trend (Additional file 1: Figure S17, S18, S19, S20).

### National burden of PUD

**Fig. 3:**
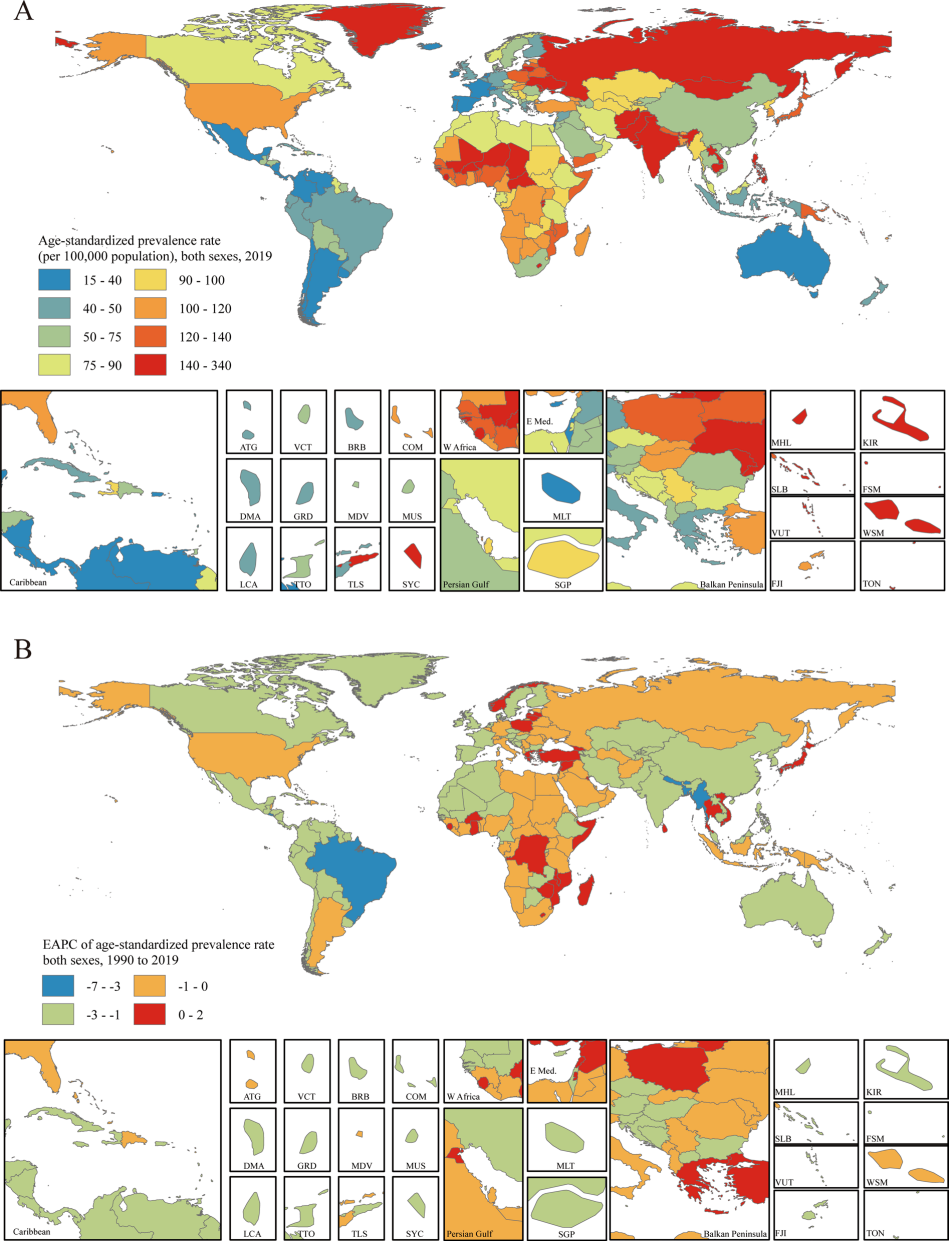
Distribution of age-standardized prevalence rates and EAPCs in age-standardized prevalence rates of PUD globally. **a** The age-standardized prevalence rate (per 100,000 population) in both sexes globally in 2019. **b** The EAPC in the age-standardized prevalence rate in both sexes globally from 1990 to 2019. Maps in Fig. 3 were designed and plotted by ArcGIS (version 9.0). *ATG* Antigua and Barbuda, *BRB* Barbados, *COM* Comoros, *DMA* Dominica, *FJI* Fiji, *FSM* Federated States of Micronesia, *GRD* Grenada, *KIR* Kiribati, *LCA* Saint Lucia, *MDV* Maldives, *MHL* Marshall Islands, *MLT* Malta, *MUS* Mauritius, *SGP* Singapore, *SLB* Solomon Islands, *SYC* Seychelles, *TLS* Timor-Leste, *TON* Tonga, *TTO* Trinidad and Tobago, *VCT* Saint Vincent and the Grenadines, *VUT* Vanuatu, *WSM* Samoa

The age-standardized prevalence rate estimated for PUD in 2019 ranged from 15.19 to 330.32 per 100,000 population. Kiribati [330.32 (95% UI 286.98 to 379.81)], Vanuatu [247.62 (95% UI 214.30 to 284.91)] and Greenland [209.77 (95% UI 182.50 to 239.31)] had the highest age-standardized prevalence rates in 2019. Israel [15.19 (95% UI 11.83 to 18.85)], Costa Rica [17.28 (95% UI 14.59 to 20.33)] and Panama [19.95 (95% UI 16.25 to 23.98)] had the lowest rates (Fig. 3a). The EAPCs in the age-standardized prevalence rates from 1990 to 2019 differed substantially between countries and territories. Turkey [1.39 (95% CI 1.01 to 1.77)], Norway [1.27 (95% CI 0.86 to 1.69)] and Ghana [0.84 (95% CI 0.43 to 1.26)] showed the largest increases, and Bangladesh [-6.80 (95% CI − 7.07 to − 6.53)], Brazil [-4.76 (95% CI − 5.16 to − 4.35)] and Bhutan [-4.24 (95% CI − 4.55 to − 3.94)] showed the largest decreases (Fig. 3b). The countries and territories with the highest age-standardized PUD prevalence rates in 2019 also had the highest age-standardized incidence rates (Additional file 1: Figure S21). The country with the highest EAPC in incidence rate was Norway [1.08 (95% CI 0.45 to 1.71)] (Additional file 1: Figure S22).

Age-standardized PUD-associated death rates in 2019 varied from 0.46 to 22.48 per 100,000 population. Cambodia [22.48 (95% UI 17.42 to 28.98)], Kiribati [21.78 (95% UI 16.38 to 27.96)] and Laos [19.24 (95% UI 14.04 to 26.14)] had the highest age-standardized death rates in 2019. Sri Lanka [0.46 (95% UI 0.32 to 0.64)], Italy [0.58 (95% UI 0.50 to 0.64)] and Israel [0.60 (95% UI 0.48 to 0.74)] had the lowest rates (Additional file 1: Figure S23a). Only eight of the 204 countries and territories showed potential increasing EAPCs in age-standardized death rates (red regions in Additional file 1: Figure S23b). Among them, only one country, Lesotho [1.19 (95% CI 0.04 to 2.36)], showed a definite increase, with all 95% CIs greater than zero. Bangladesh [-10.08 (95% CI − 11.83 to − 8.29)], the Republic of Korea [-7.34 (95% CI − 9.61 to − 5.02)] and Spain [-7.24 (95% CI − 10.46 to − 3.91)] had the largest decreases in age-standardized death rates from 1990 to 2010 (Additional file 1: Figure S23b).

### Socioeconomic profiles of PUD

**Fig. 4:**
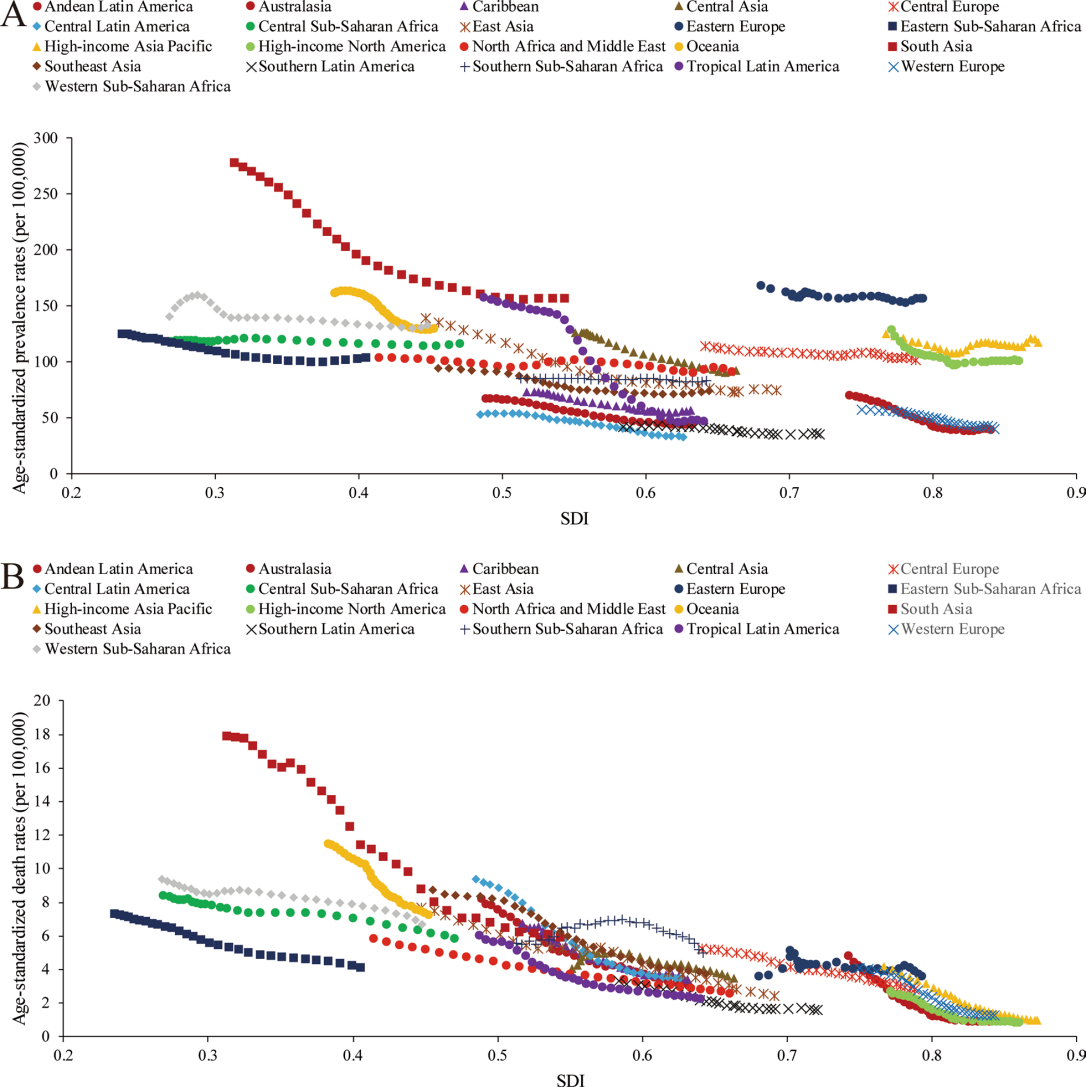
Trends of age-standardized prevalence and death rates (per 100,000 population) in 21 GBD regions by SDI from 1990 to 2019. **a** Trends of age-standardized prevalence rates; **b** trends of age-standardized death rates by SDI

**Fig. 5:**
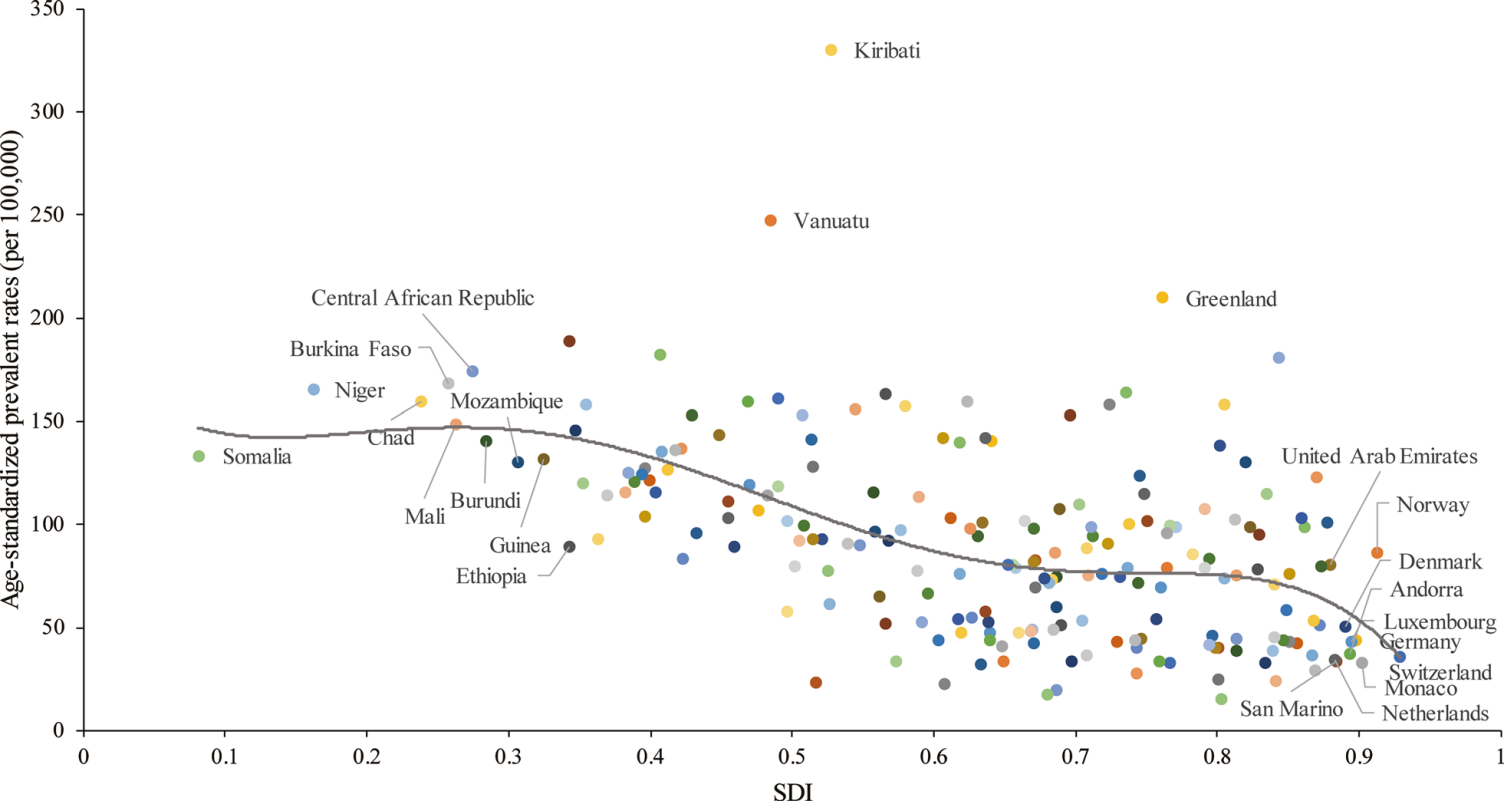
Age-standardized prevalence rates (per 100,000 population) of PUD in 204 countries globally by SDI in 2019. The gray line represents the expected age-standardized prevalence rate based on the SDI in 2019

A lower SDI was associated with higher age-standardized prevalence rates, incidence rates, DALYs and deaths associated with PUD, with values that were higher than the global rate in the two highest SDI quintiles and lower than the global rate in the three lowest SDI quintiles. The age-standardized prevalence rates in the high-SDI and low-SDI quintiles were 80.98 (95% UI 68.19 to 95.81) and 145.35 (95% UI 123.83 to 169.90) per 100,000 population in 2019, respectively (Additional file 1: Figure S24). The high-SDI quintile [1.18 (95% UI 1.04 to 1.29)] was associated with the lowest age-standardized death rate in 2019, while the low-SDI quintile [6.15 (95% UI 5.31 to 7.04)] had the second-highest rate, which was just slightly lower than that of the low-middle-SDI quintile (Additional file 1: Figure S25). In contrast, the largest decreases in age-standardized prevalence, incidence, DALY and death rates from 1990 to 2019 occurred in the low-SDI and low-middle-SDI quintiles. For the 21 GBD regions, positive associations were found between the age-standardized prevalence (Fig. 4a), incidence (Additional file 1: Figure S26), DALY (Additional file 1: Figure S27) and death (Fig. 4b) rates and SDI between 1990 and 2019. However, in the Saharan African regions, the downward trend of the prevalence rate associated with increasing SDI was not obvious. In the Southern sub-Saharan African region, the death rate showed an inverted U-shaped pattern; the death rate and SDI were positively correlated when the SDI was < 0.59 and negatively correlated when the SDI > 0.59. Positive associations between age-standardized prevalence, incidence, DALY and death rates and SDI for 204 countries and territories in 2019 were observed. The age-standardized prevalence rate was higher than the expected level in some countries such as Kiribati, Vanuatu and Greenland (Fig. 5). The estimated age-standardized DALY rate decreased when the SDI improved. Some countries and territories also had DALY rates that were significantly higher than expected, such as Kiribati, Cambodia and Laos (Additional file 1: Figure S28).

## Discussion

PUD is usually defined as a greater than 3- to 5-mm rupture of the gastric or duodenal mucosa, which is caused by an imbalance in factors that protect the gastric and duodenal mucosa and factors that can cause damage. In this study, we analyzed the prevalence trends of PUD at the global, regional and national levels from 1990 to 2019, along with PPIs medication use over the span of thirty years. The prevalence of PUD in 2019 was approximately 8.09 million worldwide, and this study exhibited continues increasing tendency in the number of prevalent. Similar to the results of other research reports [16, 17], the incidence of PUD showed a slight increase from 2006 to 2019, but the ASR showed a decreasing trend. However, in recent years, this downward trend has plateaued, which may be related to the fact that the main ulcer etiology has shifted in many countries from *H. pylori* infection to non-steroidal anti-inflammatory drug (NSAIDs) use [1]. For PUD, which is a chronic disease, it is often necessary to take appropriate drugs for a long period of time, and its recurrent characteristics and potentially serious complications have a significant impact on the social economy and medical and health costs.

PUD may be attributed to many etiologies, such as *H. pylori* infection, NSAIDs use, gastric bypass surgery, smoking, selective serotonin reuptake inhibitor use, stress, lifestyle habits and genetic characteristics, which have been identified as the main risk factors [1, 2]. During the study period, especially in the first 20 years, the incidence of and mortality due to PUD showed significant decreasing trends, which were closely related to PPIs use and the widespread administration of anti-*H. pylori* treatment, which started in the late 1980s. In the last 10 years of the study period, the incidence of and mortality due to PUD showed relatively stable trends that did not decline with the further promotion of anti-*H. pylori* treatment. However, there was an increase in the use of NSAIDs, especially aspirin and other drugs, and these drugs often lead to serious complications in patients with PUD. In previous studies, especially in Australia, a country with an inexplicable history of *H. pylori*, *H. pylori* infection was associated with 70% to 90% of PUD cases [18, 19]. Although these values are lower in some other studies, *H. pylori* infection is still a key factor in the pathogenesis of PUD [20]. Despite anti-inflammatory effect, NSAIDs are always used in antipyrexia and analgesic therapy, which makes NSAIDs as most commonly prescribed medicine [21]. Targeting cyclooxygenases enzymes (COXs), NSAIDs are divided into non-selective NSAIDs and selective COX-2 inhibitors, such as aspirin and celecoxib repectively [22]. However, NSAIDs could cause gastrointestinal adverse effects including ulcers, bleeding or perforation [21, 23]. Drugs such as aspirin and other NSAIDs account for approximately 10% of PUD cases. NSAIDs have a stronger correlation with duodenal ulcers than with gastric ulcers. In recent decades, the use of these drugs has increased dramatically [24, 25]. They account for approximately 5% to 10% of all prescription drugs each year and have shown an increasing trend [26]. In general practice, the prevalence of NSAIDs use in patients over 65 years old is as high as 96% [27]. A study from Norway indicated that approximately 7.3% of elderly patients over 60 years old took at least one NSAIDs prescription within one year period [28]. Differ from aspirin, selective COX-2 inhibitors have a weaker association with PUD than nonselective NSAIDs, which suppress COX-1 activity to inhibit gastric mucosa repair [29, 30].

For sever obesity patients, bariatric surgery, such as Roux-en-Y gastric bypass (RYGBP) surgery and duodenal switch (DS) surgery, could be a proper therapy to reduce weight and comorbidities [31]. Due to well-established procedure and nearly 70 years of surgical experience, RYGBP is considered as gold standard for bariatric surgery [32]. Although overweight and related metabolic symptoms would be reduced after gastric bypass surgery, several complications still might influence the recovery of operated patients, such as marginal ulcer (MU). MU developed at or distal to gastroenteral anastomosis and occurs in approximately 5% of obese patients undergoing gastric bypass surgery [31, 33]. In patients with upper gastrointestinal symptoms after gastric bypass surgery, the incidence can reach 27% to 36%, indicating gastric bypass surgery history might contribute to the development of PUD [1, 34].

In the vast majority of GBD regions, the incidence of and mortality due to PUD showed significant downward trends with increasing SDI; this is closely related to the awareness of *H. pylori* treatment and the appropriate management of other chronic diseases. However, in some areas, this relationship is questionable, especially in the southern sub-Saharan African region. The cause of this phenomenon is still controversial and may be related to the epidemiological characteristics and risk factors for PUD in such countries and territories. Although the incidence of *H. pylori* infection had decreased as SDIs increased in these areas, in some countries, the infection rate remains very high. NSAIDs use rates in these areas were significantly lower than those in developed countries [35, 36]. Studies have also shown that the rates of severe PUD-related complications, such as perforation of the digestive tract, are high in these countries and territories and appear to be associated with Khat intake [37]. When stratified by SDI, the incidence of PUD showed decreasing trends in different groups over time. The decrease was more obvious in low- and low-middle-SDI regions than in high-SDI regions, and the incidence of PUD tended to plateau in high-SDI regions; however, it was still far lower than those in low-SDI regions. PUD-related deaths decreased in all groups. In 2019, in different countries and regions, the prevalence of PUD generally decreased with increasing SDI, but in some Pacific islands, such as Kiribati and Vanuatu, the prevalence of PUD remained abnormally high. This may be related to the high *H. pylori* infection rate among and the ethnic characteristics of Pacific islanders. Some studies found that Maori and Pacific Island adults and children living in South Auckland had a high rate of infection with *H. pylori* compared to Europeans from the same area [38]. Data showed that household crowding involving children in New Zealand contributed to 44% of *H. pylori* infections in Pacific Islanders, 36% in Maori people and 14% in Europeans [39, 40].

The age-standardized incidence rate showed an increasing annual trend with increasing age. This is different from the incidence rate of *H. pylori*. A multicenter cross-sectional study showed that the positive rate of *H. pylori* serum antibody increased linearly from the 30- to 39-year-old age group to the 60- to 69-year-old group, while the infection rates of *H. pylori* in people aged 20 to 29 and over 70 years old were low. The change in the *H. pylori* infection rate showed a trend of initially increasing and then decreasing with age [41]. The consistent increase in the age-standardized incidence rate may be associated with an increased incidence of drug-related PUD in older people who are more likely to suffer from other chronic diseases and require other medications, such as NSAIDs. Studies have shown that the use of NSAIDs peaks at approximately 50 years old, and after 70 years old, the use of NSAIDs, especially aspirin, decreases, which is related to the increased incidence of NSAID-related adverse reactions in the elderly population [42, 43]. Estrogen can prevent ulcers by inhibiting the synthesis and release of gastrin and reducing the secretion of gastric acid. The decrease in estrogen levels in elderly females may be a protective factor for PUD [44] and may also cause a slight decrease in the growth trend of the age-standardized incidence rate in elderly women over 80 years old.

Regarding sex, at the end of the twentieth century, the incidence of and mortality due to PUD showed significant decreases, and the trends in males were more obvious than those in females. Over time, the decreasing trends of the age-standardized incidence and mortality rates became less steep, but the rates in males remained higher than those in females. PUD-related deaths and DALYs were significantly lower in females than in males. However, further analysis of PUD-related factors revealed that the incidence in females was slightly higher than that in males aged less than 20 years. In 2019, among the different GBD regions, the incidence rates of PUD in the Oceania, High-income Asia Pacific, Eastern Europe, East Asia and Central Asia regions were significantly higher in males than in females, while in other regions, there was no significant difference in the incidence between the sexes. In some regions, such as Western sub-Saharan Africa, the rate in females was significantly higher than that in males. However, the PUD-related death rate was higher in males than in females, except in Central sub-Saharan Africa. In West African countries, such as Ghana and Nigeria, the incidence of PUD in females accounted for approximately 54–57% [35]. Research results have shown that there is a sex difference in the influence of acetic acid-induced gastric ulcer formation in rats. After the administration of certain interventions, the natural defensive mechanism in the gastric mucosa was adversely disturbed in male rats but activated in female rats [45]. This phenomenon is similar to previous clinical and the current research results, although the specific mechanism is not completely clear.

There were increasing trends in the EAPCs in the age-standardized prevalence and DALY rates among only female patients in Eastern Europe (marginally increasing incidence and death rate trends) from 1990 to 2019, mainly due to the increase in the number of females in Russia and the Ukraine. In these two countries, especially in Siberia, the *H. pylori* infection rates were significantly higher than those in other regions. However, there were no differences in *H. pylori* infection rates between the sexes and among ethnicities [46, 47]. Therefore, the consistently increased risk of PUD among females in these areas may be related to factors such as drugs or bypass surgery [48, 49]. These reasons require further study to produce high-level evidence-based medical evidence.

The roles of anti-*H. pylori* therapy and acid suppression therapy in the treatment and prevention of PUD are clear. Identifying high-risk populations and preventing drug-related complications are particularly important at this stage. Anti-*H. pylori* treatment can reduce the incidence of PUD and reduce the risk of gastric cancer in high-risk groups. Similar to acid suppression therapy, it will not increase the economic burden of disease treatment. However, there is still some controversy regarding whether to administer anti-*H. pylori* treatment in all positive patients considering the extensive administration of antibiotics, as it may promote the production of other resistant bacteria, leading to the occurrence of *Clostridium difficile*-associated diarrhea, and increase the potential risk of cardiovascular disease [50, 51]. Acid suppressant drugs, mainly PPIs, seem to be the most effective class of gastroprotectants for the management of PUD [52]. Especially in recent years, the risk factors for PUD have gradually changed from *H. pylori* infection to drug-related PUD, so treatment with PPIs has gradually increased in importance. Especially in patients with gastrointestinal bleeding, PPIs can significantly reduce the incidence of adverse outcomes. However, PPIs also have certain medication risks, such as an increased risk of fracture and the development of other infections. These risks still lack strong evidence, and the benefits of PPIs greatly outweigh their associated risks. The current controversy mainly concerns the durations and doses of PPIs and whether oral PPIs have the same effect as intravenous medication in maintenance treatment [53].

The study has some limitations. First, critical information about disease burden in some countries does not exist or was unavailable, making it difficult to illustrate and understand health trends. Second, compared with other chronic noncommunicable diseases with high mortality rates, the mortality rate associated with PUD is relatively low, but PUD has the characteristic of recurrence. The high prevalence rate also results in a high disease burden. However, compared with that of malignant tumors, diabetes, and cardiovascular and cerebrovascular diseases, evidence indicating that PUD should be considered a high-priority disease is lacking [54].

## Conclusions

In conclusion, this study focused on the epidemiological characteristics of PUD in different countries and territories, different age groups and different sexes. Overall, the risk of morbidity and mortality due to PUD decreased significantly, but with the passage of time for *H. pylori* eradication, the downward trend gradually weakened, which might be related to the gradual shift in the main risk factors for PUD from *H. pylori* infection to wide use of NSAIDs. Therefore, additional medical and health-related attention is needed to control the incidence of PUD and the occurrence of adverse events.

## Supplementary Information

**Additional file 1: Table S1**. PUD prevalence in 1990 and 2019 for both sexes and estimated annual percentage change in age-standardized rates by location; **Table S2**. PUD incidence in 1990 and 2019 for both sexes and estimated annual percentage change in age-standardized rates by location; **Table S3**. DALYs of PUD in 1990 and 2019 for both sexes and estimated annual percentage change in age-standardized rates by location; **Table S4**. PUD death in 1990 and 2019 for both sexes and estimated annual percentage change in age-standardized rates by location; **Figure S1**. Incident cases with age-standardized incidence rate (per 100,000 population) changes in all years from 1990 to 2019; **Figure S2**. DALYs with age-standardized rate (per 100,000 population) changes in all years from 1990 to 2019; **Figure S3**. Deaths with age-standardized death rate (per 100,000 population) changes by age in 2019; **Figure S4**. DALYs with age-standardized DALY rate (per 100,000 population) changes by age in 2019; **Figure S5**. Age-standardized prevalent rate changes in PUD in seven super-regions in all years from 1990 to 2019; **Figure S6**. Trends of age-standardized incidence rates (per 100,000 population) in seven super-regions in all years from 1990 to 2019; **Figure S7**. Trends of age-standardized death rates (per 100,000 population) in seven super-regions in all years from 1990 to 2019; **Figure S8**. Trends of age-standardized DALY rates (per 100,000 population) in seven super-regions in all years from 1990 to 2019; **Figure S9**. Trends of prevalent cases of PUD in 21 GBD regions in all years from 1990 to 2019; **Figure S10**. Trends of incident cases of PUD in 21 GBD regions in all years from 1990 to 2019; **Figure S11**. Trends of DALYs due to PUD in 21 GBD regions in all years from 1990 to 2019; **Figure S12**. Trends of PUD-related deaths in 21 GBD regions in all years from 1990 to 2019; **Figure S13**. Age-standardized prevalence rates (per 100,000 population) of PUD in males and females in 21 GBD regions in 2019; **Figure S14**. Age-standardized incident rates (per 100,000 population) of PUD in males and females in 21 GBD regions in 2019; **Figure S15**. Age-standardized DALY rates (per 100,000 population) due to PUD in males and females in 21 GBD regions in 2019; **Figure S16**. Age-standardized death rates (per 100,000 population) due to PUD in males and females in 21 GBD regions in 2019; **Figure S17**. Estimated annual percentage changes in age-standardized prevalent rates in different regions between 1990 and 2019; **Figure S18**. Estimated annual percentages of age-standardized incident rates (per 100,000 population) in 21 GBD regions between 1999 and 2019; **Figure S19**. Estimated annual percentages of age-standardized DALY rates (per 100,000 population) in 21 GBD regions between 1999 and 2019; **Figure S20**. Estimated annual percentages of age-standardized death rates (per 100,000 population) in 21 GBD regions between 1999 and 2019; **Figure S21**. Distributions of age-standardized incidence rates (per 100,000 population) of PUD in different regions in 2019; **Figure S22**. Distributions of age-standardized incidence rates (per 100,000 population) of PUD in different regions from 1999 to 2019; **Figure S23**. Distributions of age-standardized death rates and EAPCs in age-standardized prevalence rates of PUD globally; **Figure S24**. Trends of age-standardized prevalence rates (per 100,000 population) in different SDI regions from 1990 to 2019; **Figure S25**. Age-standardized death rates (per 100,000 population) from 1990 to 2019 in different SDI regions; **Figure S26**. Trends of age-standardized incidence rates (per 100,000 population) of PUD in 21 GBD regions by SDI; **Figure S27**. Trends of age-standardized DALY rates (per 100,000 population) of PUD in 21 GBD regions by SDI; **Figure S28**. Age-standardized DALY rates (per 100,000 population) due to PUD globally in 204 countries and territories by SDI in 2019.

## Data Availability

The datasets generated and/or analyzed during the current study are available in the Global Burden of Disease, Injuries and Risk Factors Study (http://ghdx.healthdata.org/).

## Abbreviations

PUD: Peptic ulcer disease
PPI: Proton-pump inhibitor
NSAIDs: Nonsteroidal anti-inflammatory drugs
GBD: Global Burden of Disease, Injuries and Risk Factors Study
DALY: Disability-adjusted life years
ASR: Age-standardized rate
GHDx: Global Health Data Exchange database
SDI: Sociodemographic index
EAPC: Estimated annual percentage change
GLM: Generalized linear model
CI: Confidence interval
UI: Uncertainty interval
