# Supplementary material for: The global, regional and national burden of peptic ulcer disease from 1990 to 2019: a population-based study
Source: BMC Gastroenterol. 2022 Feb 10;22:58. doi: 10.1186/s12876-022-02130-2 (PMC8832644; doi:10.1186/s12876-022-02130-2)
Supplement: Supplementary file 1 — Additional file 1: Table S1. PUD prevalence in 1990 and 2019 for both sexes and estimated annual percentage change in age-standardized rates by location; Table S2. PUD incidence in 1990 and 2019 for both sexes and estimated annual percentage change in age-standardized rates by location; Table S3. DALYs of PUD in 1990 and 2019 for both sexes and estimated annual percentage change in age-standardized rates by location; Table S4. PUD death in 1990 and 2019 for both sexes and estimated annual percentage change in age-standardized rates by location; Figure S1. Incident cases with age-standardized incidence rate (per 100,000 population) changes in all years from 1990 to 2019; Figure S2. DALYs with age-standardized rate (per 100,000 population) changes in all years from 1990 to 2019; Figure S3. Deaths with age-standardized death rate (per 100,000 population) changes by age in 2019; Figure S4. DALYs with age-standardized DALY rate (per 100,000 population) changes by age in 2019; Figure S5. Age-standardized prevalent rate changes in PUD in seven super-regions in all years from 1990 to 2019; Figure S6. Trends of age-standardized incidence rates (per 100,000 population) in seven super-regions in all years from 1990 to 2019; Figure S7. Trends of age-standardized death rates (per 100,000 population) in seven super-regions in all years from 1990 to 2019; Figure S8. Trends of age-standardized DALY rates (per 100,000 population) in seven super-regions in all years from 1990 to 2019; Figure S9. Trends of prevalent cases of PUD in 21 GBD regions in all years from 1990 to 2019; Figure S10. Trends of incident cases of PUD in 21 GBD regions in all years from 1990 to 2019; Figure S11. Trends of DALYs due to PUD in 21 GBD regions in all years from 1990 to 2019; Figure S12. Trends of PUD-related deaths in 21 GBD regions in all years from 1990 to 2019; Figure S13. Age-standardized prevalence rates (per 100,000 population) of PUD in males and females in 21 GBD regions in 2019; Figure S14. Ag [file 12876_2022_2130_MOESM1_ESM.docx]

**Additional files**

Supplement to: **The global, regional and national burden of peptic ulcer disease from 1990 to 2019: a population-based study**

**Contents:**

Additional file 1: Table S1. PUD prevalence in 1990 and 2019 for both sexes and estimated annual percentage change in age-standardized rates by location.

Additional file 1: Table S2. PUD incidence in 1990 and 2019 for both sexes and estimated annual percentage change in age-standardized rates by location.

Additional file 1: Table S3. DALYs of PUD in 1990 and 2019 for both sexes and estimated annual percentage change in age-standardized rates by location.

Additional file 1: Table S4. PUD death in 1990 and 2019 for both sexes and estimated annual percentage change in age-standardized rates by location.

Additional file 1: Figure S1. Incident cases with age-standardized incidence rate (per 100,000 population) changes in all years from 1990 to 2019.

Additional file 1: Figure S2. DALYs with age-standardized rate (per 100,000 population) changes in all years from 1990 to 2019.

Additional file 1: Figure S3. Deaths with age-standardized death rate (per 100,000 population) changes by age in 2019.

Additional file 1: Figure S4. DALYs with age-standardized DALY rate (per 100,000 population) changes by age in 2019.

Additional file 1: Figure S5. Age-standardized prevalent rate changes in PUD in seven super-regions in all years from 1990 to 2019.

Additional file 1: Figure S6. Trends of age-standardized incidence rates (per 100,000 population) in seven super-regions in all years from 1990 to 2019.

Additional file 1: Figure S7. Trends of age-standardized death rates (per 100,000 population) in seven super-regions in all years from 1990 to 2019.

Additional file 1: Figure S8. Trends of age-standardized DALY rates (per 100,000 population) in seven super-regions in all years from 1990 to 2019.

Additional file 1: Figure S9. Trends of prevalent cases of PUD in 21 GBD regions in all years from 1990 to 2019.

Additional file 1: Figure S10. Trends of incident cases of PUD in 21 GBD regions in all years from 1990 to 2019.

Additional file 1: Figure S11. Trends of DALYs due to PUD in 21 GBD regions in all years from 1990 to 2019.

Additional file 1: Figure S12. Trends of PUD-related deaths in 21 GBD regions in all years from 1990 to 2019.

Additional file 1: Figure S13. Age-standardized prevalence rates (per 100,000 population) of PUD in males and females in 21 GBD regions in 2019.

Additional file 1: Figure S14. Age-standardized incident rates (per 100,000 population) of PUD in males and females in 21 GBD regions in 2019.

Additional file 1: Figure S15. Age-standardized DALY rates (per 100,000 population) due to PUD in males and females in 21 GBD regions in 2019.

Additional file 1: Figure S16. Age-standardized death rates (per 100,000 population) due to PUD in males and females in 21 GBD regions in 2019.

Additional file 1: Figure S17. Estimated annual percentage changes in age-standardized prevalent rates in different regions between 1990 and 2019.

Additional file 1: Figure S18. Estimated annual percentages of age-standardized incident rates (per 100,000 population) in 21 GBD regions between 1999 and 2019.

Additional file 1: Figure S19. Estimated annual percentages of age-standardized DALY rates (per 100,000 population) in 21 GBD regions between 1999 and 2019.

Additional file 1: Figure S20. Estimated annual percentages of age-standardized death rates (per 100,000 population) in 21 GBD regions between 1999 and 2019.

Additional file 1: Figure S21. Distributions of age-standardized incidence rates (per 100,000 population) of PUD in different regions in 2019.

Additional file 1: Figure S22. Distributions of age-standardized incidence rates (per 100,000 population) of PUD in different regions from 1999 to 2019.

Additional file 1: Figure S23. Distributions of age-standardized death rates and EAPCs in age-standardized prevalence rates of PUD globally.

Additional file 1: Figure S24. Trends of age-standardized prevalence rates (per 100,000 population) in different SDI regions from 1990 to 2019.

Additional file 1: Figure S25. Age-standardized death rates (per 100,000 population) from 1990 to 2019 in different SDI regions.

Additional file 1: Figure S26. Trends of age-standardized incidence rates (per 100,000 population) of PUD in 21 GBD regions by SDI.

Additional file 1: Figure S27. Trends of age-standardized DALY rates (per 100,000 population) of PUD in 21 GBD regions by SDI.

Additional file 1: Figure S28. Age-standardized DALY rates (per 100,000 population) due to PUD globally in 204 countries and territories by SDI in 2019.

**Additional file 1:**

**Table S1. PUD prevalence in 1990 and 2019 for both sexes and estimated annual percentage change in age-standardized rates by location**

| **Characteristic** | **Rate^*^** | | **Number** | | **EAPC^¶^** |
| --- | --- | --- | --- | --- | --- |
|  | **No (95% UI)** | | **No (95% UI)** | | **No (95% CI)** |
|  | **1990** | **2019** | **1990** | **2019** | **1990-2019** |
| **Global** | 143.37  (120.54 - 170.25) | 99.4  (83.86 - 117.55) | 6434103.07  (5405962.92 - 7627970.56) | 8090475.71  (6794576.11 - 9584000.2) | -1.45  (-1.83 - -1.07) |
| **Central Asia** | 126.16  (109.34 - 144.92) | 92.65  (79.84 - 106.92) | 69744.94  (60863.86 - 80521.83) | 82145.34  (70420.08 - 95427.79) | -1.36  (-1.75 - -0.97) |
| **Armenia** | 157.1  (135.62 - 180.59) | 107.39  (92.86 - 123.47) | 4880.98  (4207.06 - 5633.7) | 4121.72  (3570.71 - 4693.72) | -1.71  (-2.08 - -1.33) |
| **Azerbaijan** | 117.09  (100.71 - 135) | 73.4  (61.74 - 87.44) | 7265.51  (6214.05 - 8452.34) | 8181.78  (6860.21 - 9710.03) | -2.17  (-2.59 - -1.74) |
| **Georgia** | 99.02  (84.65 - 114.62) | 109.44  (94.33 - 126.71) | 5994.04  (5080.03 - 6938.92) | 5174.3  (4488.63 - 5896.71) | 0.43  (0 - 0.86) |
| **Kazakhstan** | 140.11  (120.97 - 161.61) | 90.8  (78.25 - 105.6) | 20229.99  (17453.54 - 23421.16) | 17229.61  (14815.11 - 20064.81) | -1.8  (-2.18 - -1.41) |
| **Kyrgyzstan** | 127.16  (109.8 - 145.86) | 66.64  (56.84 - 78.25) | 4374.69  (3753.71 - 5048) | 3948.04  (3314.74 - 4717.6) | -2.62  (-3.06 - -2.18) |
| **Mongolia** | 145.54  (124.95 - 168.24) | 142.16  (122.88 - 163.6) | 1957.91  (1679.08 - 2285.31) | 4095.38  (3526.87 - 4737.22) | 0  (-0.35 - 0.34) |
| **Tajikistan** | 140.37  (121.34 - 162.26) | 90.4  (77.73 - 104.64) | 5135.66  (4408.79 - 5967.66) | 6759.05  (5691.68 - 7965.25) | -1.98  (-2.35 - -1.6) |
| **Turkmenistan** | 101.95  (87.58 - 118.74) | 98.2  (83.89 - 114.25) | 2710.23  (2308.89 - 3184.16) | 4905.3  (4171.46 - 5733.91) | -0.19  (-0.6 - 0.22) |
| **Uzbekistan** | 118.95  (103.93 - 136.21) | 94.25  (81.84 - 108.77) | 17195.92  (14835.86 - 19923.34) | 27730.16  (23674.71 - 32439.31) | -1.19  (-1.57 - -0.81) |
| **Central Europe** | 113.95  (98.13 - 132.05) | 101.81  (87.89 - 118.26) | 161727.98  (139697.21 - 187060.56) | 174813.72  (151160.09 - 200645.59) | -0.28  (-0.68 - 0.12) |
| **Albania** | 73.36  (61.6 - 87.66) | 71.83  (56.41 - 89.03) | 1922.19  (1594.96 - 2326.94) | 2415.87  (1925.99 - 2991.3) | -0.22  (-0.73 - 0.29) |
| **Bosnia and Herzegovina** | 112.18  (95.59 - 130.57) | 76.06  (63.17 - 90.53) | 4962.57  (4216.76 - 5838.03) | 3597.44  (3019.01 - 4232.01) | -1.5  (-1.95 - -1.06) |
| **Bulgaria** | 101.56  (88.44 - 116.46) | 79.03  (67.25 - 92.55) | 11629.83  (10134.87 - 13308.57) | 8523.06  (7303.93 - 9814.46) | -1.01  (-1.46 - -0.56) |
| **Croatia** | 115.47  (100.7 - 133) | 83.63  (71.7 - 97.39) | 7073.49  (6157.15 - 8131.64) | 5690.59  (4929.08 - 6526.51) | -1.3  (-1.7 - -0.9) |
| **Czechia** | 102.22  (89.23 - 117.16) | 78.32  (66.81 - 91.59) | 13239.07  (11574.74 - 15049.69) | 12903.97  (11122.43 - 14866.15) | -1.12  (-1.54 - -0.69) |
| **Hungary** | 146.32  (128.8 - 166.16) | 107.87  (93.56 - 125) | 19790.35  (17395.18 - 22395.89) | 16665.26  (14510.77 - 19029.03) | -1.29  (-1.65 - -0.93) |
| **Montenegro** | 94.31  (80.89 - 109.01) | 78.81  (66.33 - 93.69) | 607.31  (518.45 - 702.24) | 650.75  (547.77 - 759.93) | -0.69  (-1.13 - -0.25) |
| **North Macedonia** | 90.56  (78.11 - 104.9) | 71.74  (59.21 - 85.63) | 1793.84  (1541.04 - 2087.81) | 2014.15  (1671.42 - 2395.03) | -1.07  (-1.53 - -0.61) |
| **Poland** | 129.45  (106.65 - 157.03) | 138.55  (119.26 - 161.1) | 54822.79  (45094.84 - 66750.15) | 79919.86  (68390.12 - 94019.16) | 0.6  (0.23 - 0.97) |
| **Romania** | 88.91  (76.58 - 103.46) | 69.52  (57.92 - 83.32) | 23869.08  (20572.25 - 27574.06) | 18722.05  (15797.89 - 21901.44) | -0.43  (-0.88 - 0.02) |
| **Serbia** | 108.6  (93.34 - 124.4) | 99.26  (85.94 - 114.45) | 12218.62  (10496.27 - 13942.01) | 13569.6  (11705.07 - 15483.87) | -0.08  (-0.48 - 0.32) |
| **Slovakia** | 122.98  (106.32 - 141.49) | 102.47  (87.04 - 120.38) | 7096.83  (6135.72 - 8136.32) | 7906.98  (6743.73 - 9103.88) | -0.91  (-1.29 - -0.54) |
| **Slovenia** | 113.62  (98.64 - 129.59) | 70.97  (58.02 - 85.2) | 2702.01  (2342.3 - 3077.13) | 2234.14  (1861.15 - 2640.75) | -2.12  (-2.55 - -1.69) |
| **Eastern Europe** | 168.1  (137.65 - 203.08) | 156.97  (129.18 - 188.79) | 439449.91  (359260.76 - 532690.52) | 439984.88  (363828.61 - 533969.23) | -0.18  (-0.51 - 0.15) |
| **Belarus** | 142.94  (122.01 - 167.19) | 123.6  (103.58 - 147.1) | 17093.36  (14603.47 - 19800.52) | 15066.05  (12812.88 - 17624.16) | -0.54  (-0.91 - -0.18) |
| **Estonia** | 140.35  (121.88 - 162.05) | 115.21  (97.45 - 136.72) | 2622.27  (2283.17 - 3016.39) | 2041.15  (1761.68 - 2351.43) | -0.23  (-0.58 - 0.12) |
| **Latvia** | 150.93  (130.57 - 175.88) | 130.61  (110.82 - 155.38) | 4825.57  (4195.81 - 5563.52) | 3519.21  (3053.17 - 4055.51) | 0.01  (-0.32 - 0.35) |
| **Lithuania** | 156.14  (132.97 - 184.94) | 180.78  (155.85 - 210.78) | 6453.87  (5535.96 - 7592.51) | 7311.27  (6400.2 - 8363.8) | 0.67  (0.35 - 0.98) |
| **Republic of Moldova** | 179.01  (154.19 - 206.82) | 153.2  (132.76 - 178.51) | 8197.49  (7056.84 - 9448.52) | 7374.51  (6423.73 - 8470.56) | -0.54  (-0.86 - -0.21) |
| **Russian Federation** | 162.08  (131.43 - 196.38) | 157.72  (129.92 - 191.08) | 279490.16  (226587.9 - 341077.86) | 310300.75  (254048.7 - 380291.64) | -0.01  (-0.34 - 0.32) |
| **Ukraine** | 190.82  (154.98 - 231.2) | 163.9  (132.83 - 200.26) | 120767.2  (97739.72 - 147354.56) | 94371.94  (75679.6 - 115085.12) | -0.59  (-0.91 - -0.27) |
| **Australasia** | 70.02  (60.32 - 80.72) | 39.6  (31.96 - 48.03) | 16165.5  (13904.14 - 18691.58) | 15950.31  (13142.69 - 19216.13) | -2.43  (-3.01 - -1.85) |
| **Australia** | 69.01  (59.35 - 80.14) | 38.53  (30.61 - 47.29) | 13264.36  (11400.38 - 15368.6) | 13009.05  (10547.16 - 15841.16) | -2.52  (-3.11 - -1.93) |
| **New Zealand** | 75.01  (61.47 - 91.58) | 45.33  (38.39 - 53.72) | 2901.14  (2373.54 - 3567.27) | 2941.26  (2497.27 - 3514.31) | -2.03  (-2.57 - -1.5) |
| **High-income Asia Pacific** | 125.36  (104.56 - 149.39) | 117.1  (95.43 - 141.63) | 245335.2  (203920.33 - 293877.57) | 335708.28  (279074.64 - 404674.34) | -0.01  (-0.39 - 0.38) |
| **Brunei Darussalam** | 222.05  (190.5 - 256.42) | 98.79  (82.57 - 118.64) | 297.99  (252.91 - 350.22) | 415.24  (332.55 - 510.47) | -3.59  (-3.93 - -3.24) |
| **Japan** | 124.31  (102.53 - 149.41) | 123.28  (101.28 - 148.36) | 192125.32  (157607.12 - 232488.48) | 255491.45  (214268.73 - 305616.51) | 0.34  (-0.04 - 0.72) |
| **Republic of Korea** | 134.27  (114.99 - 155.29) | 101.15  (80.62 - 124.6) | 49126.4  (41375.48 - 58182.72) | 72545.78  (56823.23 - 89914.43) | -1.39  (-1.78 - -1) |
| **Singapore** | 139.7  (120.01 - 162.46) | 99.04  (78.51 - 122.78) | 3785.49  (3202.64 - 4506.86) | 7255.81  (5635.83 - 9182.01) | -1.44  (-1.83 - -1.06) |
| **High-income North America** | 128.46  (107.02 - 154.14) | 100.65  (86.67 - 116.47) | 436149.28  (363548.53 - 524616.35) | 541480.06  (466230.27 - 632344.61) | -0.62  (-1.02 - -0.22) |
| **Canada** | 112.34  (96.19 - 130.49) | 79.45  (65.3 - 95.93) | 35632.91  (30518.77 - 41417.29) | 42158.34  (35389.78 - 50270.96) | -1.44  (-1.88 - -1) |
| **Greenland** | 383.1  (331.27 - 435.18) | 209.77  (182.5 - 239.31) | 144.17  (124.96 - 164.68) | 141.59  (122.98 - 161.68) | -2.53  (-2.78 - -2.28) |
| **United States of America** | 130.18  (107.91 - 157.06) | 103.26  (89.26 - 119.57) | 400362.23  (332462.34 - 484861.88) | 499171.54  (428251.14 - 583178.49) | -0.53  (-0.93 - -0.14) |
| **Southern Latin America** | 42.13  (35.89 - 49.04) | 35.43  (29.47 - 41.98) | 19860.02  (16937.24 - 23198.68) | 27349.3  (22906.92 - 32304.18) | -0.79  (-1.45 - -0.13) |
| **Argentina** | 40.22  (33.98 - 47.14) | 36.45  (30.4 - 43.18) | 12963.74  (10962.06 - 15186.56) | 18455.69  (15444.98 - 21791.07) | -0.43  (-1.11 - 0.25) |
| **Chile** | 47.95  (41.13 - 55.02) | 33.55  (27.68 - 40.29) | 5317.2  (4553.38 - 6193.58) | 7419.71  (6142.36 - 8878.08) | -1.58  (-2.18 - -0.97) |
| **Uruguay** | 43.57  (36.94 - 50.92) | 33.49  (27.42 - 40.11) | 1578.28  (1345.05 - 1827.69) | 1472.53  (1234.1 - 1750.19) | -1.1  (-1.77 - -0.42) |
| **Western Europe** | 57.31  (48.84 - 66.83) | 39.97  (32.52 - 48.25) | 299184.7  (257782.32 - 346967.9) | 267119.65  (222158.18 - 318411.81) | -1.29  (-1.87 - -0.7) |
| **Andorra** | 47.21  (39.79 - 55.66) | 37.18  (29.9 - 45.28) | 27.08  (22.46 - 32.23) | 46.86  (37.59 - 57.33) | -1.07  (-1.71 - -0.43) |
| **Austria** | 104.39  (89.38 - 120.52) | 58.5  (47 - 71.71) | 10460.38  (9026.07 - 11973.75) | 7317.81  (5946.9 - 8822.18) | -2.44  (-2.9 - -1.98) |
| **Belgium** | 56.62  (48.25 - 65.93) | 43.44  (34.47 - 53.82) | 7712.85  (6620.99 - 8834.32) | 7066.72  (5764.03 - 8527.92) | -0.54  (-1.09 - 0.02) |
| **Cyprus** | 32.14  (27.24 - 37.69) | 24.08  (19.57 - 29.45) | 246.42  (208.32 - 290.03) | 421.43  (342.77 - 513.93) | -1.09  (-1.87 - -0.3) |
| **Denmark** | 80  (69.44 - 90.95) | 50.69  (42.63 - 59.76) | 5981.31  (5191.12 - 6783.44) | 4683.49  (4028.1 - 5425.94) | -1.66  (-2.13 - -1.18) |
| **Finland** | 68.78  (59.36 - 79.34) | 42.49  (34.94 - 50.76) | 4549.69  (3923.09 - 5242.56) | 3612.32  (3041.41 - 4243.68) | -2.01  (-2.56 - -1.47) |
| **France** | 47.67  (40.44 - 55.36) | 32.7  (25.66 - 40.36) | 35844.68  (30819.71 - 41307.37) | 30579.57  (24608.07 - 37028.17) | -1.77  (-2.44 - -1.09) |
| **Germany** | 56.36  (48.3 - 65.41) | 43.82  (35.56 - 53.41) | 63645.88  (54623.2 - 72995.71) | 59479.51  (49713.89 - 69992.58) | -0.35  (-0.9 - 0.21) |
| **Greece** | 37.79  (31.15 - 45) | 41.95  (34.66 - 49.87) | 5114.21  (4275.52 - 6017.3) | 7659.21  (6529.05 - 8950.94) | 0.4  (-0.26 - 1.06) |
| **Iceland** | 42.24  (35.6 - 49.83) | 29.17  (22.95 - 36.27) | 114.52  (97.11 - 134.07) | 132.07  (105.84 - 162.76) | -1.03  (-1.7 - -0.35) |
| **Ireland** | 66.23  (57.26 - 75.96) | 36.63  (29.42 - 45.12) | 2621.83  (2259.21 - 3001.75) | 2357.55  (1912.07 - 2870.41) | -2.58  (-3.14 - -2.01) |
| **Israel** | 26.65  (22.51 - 30.96) | 15.19  (11.83 - 18.85) | 1276.65  (1086.25 - 1480.5) | 1559.2  (1234.93 - 1920.06) | -2.67  (-3.57 - -1.76) |
| **Italy** | 52.79  (43.27 - 64.06) | 40.43  (33.41 - 48.73) | 41633.75  (34501.4 - 50657.72) | 43056.59  (35819.65 - 52056.54) | -0.94  (-1.53 - -0.35) |
| **Luxembourg** | 53.74  (44.97 - 62.95) | 43.03  (33.66 - 53.6) | 268.71  (226.36 - 311.58) | 361.98  (283.14 - 449.62) | -1.2  (-1.75 - -0.64) |
| **Malta** | 40.32  (34.24 - 46.8) | 24.56  (20.01 - 29.48) | 170.67  (144.85 - 198.59) | 177.13  (147.67 - 210.28) | -1.95  (-2.67 - -1.22) |
| **Monaco** | 30.91  (24.25 - 38.56) | 32.58  (25.03 - 41.16) | 14.96  (12.13 - 18.32) | 19.32  (15.09 - 24.19) | 0.16  (-0.59 - 0.92) |
| **Netherlands** | 51.27  (43.67 - 59.58) | 34  (26.51 - 41.91) | 9754.18  (8353.35 - 11250.79) | 8476.22  (6747.08 - 10392.04) | -1.8  (-2.44 - -1.16) |
| **Norway** | 91.64  (74.95 - 110.37) | 86.25  (69.01 - 105.16) | 5252.04  (4347.9 - 6344.64) | 6121.17  (4934.17 - 7438.18) | 1.27  (0.86 - 1.69) |
| **Portugal** | 42.93  (36.66 - 49.54) | 27.73  (22.25 - 33.7) | 5542.35  (4744.06 - 6363.1) | 4854.99  (4012.79 - 5783.41) | -2.1  (-2.8 - -1.4) |
| **San Marino** | 35.35  (28.64 - 43.03) | 33.85  (26.6 - 42.13) | 10.37  (8.48 - 12.48) | 16.15  (12.91 - 19.9) | -0.23  (-0.95 - 0.49) |
| **Spain** | 48.41  (41.09 - 56.07) | 32.89  (25.56 - 40.9) | 23936.04  (20461.58 - 27534.94) | 22634.44  (17826.53 - 28065.82) | -1.84  (-2.51 - -1.17) |
| **Sweden** | 67.86  (55.7 - 82.53) | 51.36  (41.01 - 63.08) | 8639.33  (7132.41 - 10497.82) | 7720.42  (6266.11 - 9424.38) | -1.18  (-1.7 - -0.65) |
| **Switzerland** | 40.05  (32.95 - 47.74) | 35.52  (27.9 - 43.73) | 3705.91  (3101.69 - 4364.91) | 4625.93  (3699.52 - 5633.41) | -0.56  (-1.2 - 0.09) |
| **United Kingdom** | 75.57  (62.9 - 91.21) | 44.11  (36.7 - 53.09) | 62412.48  (51617.86 - 76043.12) | 43906.71  (36887.56 - 52798.61) | -2.2  (-2.73 - -1.65) |
| **Andean Latin America** | 67.17  (58.44 - 76.77) | 43.37  (37.31 - 50.57) | 17462.47  (15082.74 - 20397.06) | 25931.83  (22164.34 - 30508.75) | -1.76  (-2.32 - -1.21) |
| **Bolivia (Plurinational State of)** | 86.2  (73.52 - 99.92) | 51.9  (44.56 - 60.57) | 3515.44  (3021.38 - 4091.1) | 5121.11  (4336.01 - 6060.77) | -2.21  (-2.71 - -1.71) |
| **Ecuador** | 71.08  (62.03 - 80.49) | 44.04  (38.85 - 49.55) | 4753.09  (4089.73 - 5469.25) | 6997.12  (6179.03 - 7881.9) | -1.81  (-2.34 - -1.28) |
| **Peru** | 60.31  (52.2 - 70.14) | 40.94  (34.17 - 48.85) | 9193.93  (7843.37 - 10885.06) | 13813.61  (11476.1 - 16610.4) | -1.57  (-2.15 - -0.98) |
| **Caribbean** | 73.13  (63.41 - 83.89) | 56.31  (47.71 - 66.15) | 21482.03  (18558.64 - 24849.91) | 28403.4  (24105.85 - 33182.72) | -1.18  (-1.7 - -0.66) |
| **Antigua and Barbuda** | 46.02  (39.23 - 53.97) | 40.52  (32.93 - 49.32) | 25.4  (21.44 - 30.14) | 41.25  (33.35 - 50.1) | -0.64  (-1.28 - 0) |
| **Bahamas** | 53.93  (46.51 - 62.32) | 46.35  (39.43 - 54.7) | 106.35  (89.7 - 126.13) | 192.13  (163.38 - 227.89) | -0.78  (-1.36 - -0.2) |
| **Barbados** | 59.16  (51.08 - 68.2) | 43.98  (37.15 - 51.94) | 162.31  (141.32 - 186.93) | 177.48  (151.62 - 204.84) | -1.35  (-1.92 - -0.77) |
| **Belize** | 49.36  (42.12 - 57.48) | 43.98  (36.29 - 53.01) | 59.65  (49.69 - 71.23) | 161.21  (131.4 - 197.23) | -0.63  (-1.24 - -0.03) |
| **Bermuda** | 56.81  (48.94 - 65.59) | 44.58  (36.44 - 53.63) | 37.03  (31.6 - 43.12) | 42.35  (35.51 - 49.87) | -1.18  (-1.76 - -0.59) |
| **Cuba** | 64.63  (55.86 - 74.76) | 47.89  (39.98 - 57.4) | 6921.76  (5964.78 - 8026.31) | 7419.07  (6276.86 - 8731.51) | -1.34  (-1.9 - -0.78) |
| **Dominica** | 54.83  (46.97 - 63.55) | 43.26  (36.51 - 51.42) | 37.06  (31.85 - 42.98) | 34.66  (29.41 - 40.49) | -1.07  (-1.66 - -0.47) |
| **Dominican Republic** | 56.73  (49.04 - 65.42) | 52.56  (44.51 - 62.05) | 2901.39  (2467.74 - 3433.52) | 5432.93  (4563.82 - 6445.16) | -0.46  (-1.02 - 0.1) |
| **Grenada** | 65.52  (56.81 - 75.18) | 49.31  (42.25 - 58.14) | 45.91  (39.5 - 52.7) | 55.47  (47.31 - 65.64) | -1.31  (-1.86 - -0.76) |
| **Guyana** | 115.84  (101.36 - 132.15) | 76.33  (66.13 - 87.24) | 593.54  (516.93 - 680.37) | 543.6  (466.87 - 629.05) | -1.65  (-2.08 - -1.21) |
| **Haiti** | 132.93  (113.09 - 154.91) | 95.82  (81.64 - 111.55) | 5747.81  (4909.41 - 6681.69) | 8605.79  (7328.5 - 10061.01) | -1.38  (-1.77 - -1) |
| **Jamaica** | 69.07  (59.6 - 79.34) | 48.78  (41.18 - 58.3) | 1277.85  (1101.79 - 1476.05) | 1466.18  (1240.06 - 1754.08) | -1.61  (-2.15 - -1.08) |
| **Puerto Rico** | 47.37  (40.33 - 55.58) | 38.46  (30.27 - 47.84) | 1719.65  (1466.33 - 2021.96) | 1743.02  (1387.65 - 2132.17) | -1.03  (-1.68 - -0.37) |
| **Saint Kitts and Nevis** | 60.61  (52.63 - 69.51) | 44.6  (37.3 - 52.78) | 21.9  (18.89 - 25.09) | 30.28  (25.25 - 35.92) | -1.46  (-2.04 - -0.88) |
| **Saint Lucia** | 53.88  (46.41 - 62.08) | 42.3  (35.48 - 50.87) | 53.98  (45.98 - 63.36) | 87.47  (72.6 - 104.57) | -1.17  (-1.78 - -0.57) |
| **Saint Vincent and the Grenadines** | 78.06  (67.64 - 89.16) | 54.6  (46.69 - 63.38) | 62.04  (53.38 - 71.58) | 70.6  (60.45 - 81.45) | -1.61  (-2.11 - -1.09) |
| **Suriname** | 82.76  (71.73 - 94.85) | 58.11  (49.81 - 67.39) | 249.65  (214.48 - 288.66) | 351.12  (300.81 - 407.19) | -1.59  (-2.07 - -1.1) |
| **Trinidad and Tobago** | 74.4  (64.54 - 85.08) | 54.17  (46.21 - 63.03) | 701.17  (605.83 - 808.3) | 931.62  (794.63 - 1080.51) | -1.26  (-1.76 - -0.75) |
| **United States Virgin Islands** | 43.32  (36.78 - 51.06) | 40.19  (33.19 - 48.32) | 41.96  (35.31 - 50) | 55.01  (45.96 - 64.81) | -0.41  (-1.06 - 0.25) |
| **Central Latin America** | 53.05  (45.79 - 61.37) | 33.05  (28.46 - 38.46) | 53254.52  (45666.33 - 62249.84) | 80456.14  (68953.63 - 94070.4) | -1.85  (-2.47 - -1.24) |
| **Colombia** | 50.48  (44.38 - 57.4) | 32.31  (26.59 - 38.89) | 10535.05  (9143.59 - 12253.36) | 16736.64  (13781.48 - 20167.39) | -1.58  (-2.2 - -0.95) |
| **Costa Rica** | 25.15  (21.62 - 28.87) | 17.28  (14.59 - 20.33) | 467.18  (399.95 - 541.07) | 885.95  (746.57 - 1041.07) | -1.58  (-2.47 - -0.68) |
| **El Salvador** | 73.26  (64.31 - 83.22) | 33.95  (29.39 - 39.02) | 2420.75  (2120.08 - 2756.01) | 2050.86  (1772.09 - 2360.27) | -3.29  (-3.87 - -2.71) |
| **Guatemala** | 97.76  (85.46 - 110.77) | 61.45  (53.9 - 69.37) | 4826.42  (4178.99 - 5546.8) | 8046.51  (7037.97 - 9197.98) | -1.85  (-2.26 - -1.44) |
| **Honduras** | 78.86  (68.58 - 90.52) | 57.58  (49.93 - 66.24) | 2166.28  (1864.91 - 2494.54) | 4011.88  (3466.02 - 4619.03) | -1.53  (-2.01 - -1.05) |
| **Mexico** | 55.3  (46.1 - 66.34) | 33.52  (28.31 - 39.99) | 28025.03  (23149.76 - 33588.62) | 40048.63  (33710.7 - 47912.72) | -1.97  (-2.58 - -1.36) |
| **Nicaragua** | 32.83  (28.34 - 37.74) | 23.34  (19.56 - 27.59) | 662.3  (563.34 - 782.12) | 1234.3  (1019.28 - 1481.02) | -1.46  (-2.24 - -0.68) |
| **Panama** | 26.39  (22.71 - 30.41) | 19.95  (16.25 - 23.98) | 445.19  (380.52 - 517.81) | 836.65  (678.64 - 1011.06) | -1.36  (-2.22 - -0.48) |
| **Venezuela (Bolivarian Republic of)** | 32.13  (27.85 - 36.83) | 22.46  (18.97 - 26.35) | 3706.31  (3174.09 - 4284.87) | 6604.74  (5539.64 - 7809.57) | -1.56  (-2.34 - -0.78) |
| **Tropical Latin America** | 157.65  (131.9 - 189.59) | 47.35  (39.62 - 57.08) | 171525.71  (142478.41 - 204817.24) | 115939.07  (96299.73 - 140459.34) | -4.72  (-5.12 - -4.31) |
| **Brazil** | 159.09  (132.88 - 191.66) | 47.19  (39.51 - 56.97) | 168953.8  (140258.39 - 202124.76) | 112662.33  (93322.37 - 136592.7) | -4.76  (-5.16 - -4.35) |
| **Paraguay** | 98.7  (85.05 - 113.66) | 52.35  (44.26 - 61.75) | 2571.91  (2188.66 - 3015.66) | 3276.73  (2730.41 - 3938.63) | -2.41  (-2.9 - -1.91) |
| **North Africa and Middle East** | 103.99  (88.17 - 122.22) | 91.05  (75.32 - 109.9) | 255741.16  (213147.66 - 308510.31) | 522218.09  (424464.85 - 637548.73) | -0.37  (-0.78 - 0.05) |
| **Afghanistan** | 220.71  (187.99 - 256.05) | 188.59  (162.33 - 219.47) | 18896.12  (16068.93 - 21956.77) | 45214.37  (38275.31 - 53269.49) | -0.76  (-1.03 - -0.49) |
| **Algeria** | 103.26  (88.12 - 121.14) | 80.67  (67.49 - 96.94) | 17036.88  (14200.24 - 20447.53) | 32260.01  (26456.01 - 39265.1) | -1.23  (-1.67 - -0.79) |
| **Bahrain** | 143.63  (123.1 - 165.55) | 101.52  (84.34 - 120.89) | 383.97  (313.71 - 471.4) | 1437.88  (1121.33 - 1819.85) | -1.51  (-1.88 - -1.14) |
| **Egypt** | 87.71  (73.92 - 103.41) | 78.66  (65.6 - 94.17) | 36642.85  (30592.11 - 43421.13) | 68566.63  (56436.32 - 83021.95) | -0.55  (-1.02 - -0.09) |
| **Iran (Islamic Republic of)** | 106.71  (88.42 - 128.06) | 81.75  (66.51 - 99.36) | 41498.26  (33735.33 - 50384.48) | 70763.35  (56631.31 - 87439.7) | -1.15  (-1.58 - -0.73) |
| **Iraq** | 73.97  (62 - 88.45) | 69.46  (55.71 - 86.16) | 8627.12  (7077.72 - 10605.51) | 26019.81  (20214.8 - 32948.99) | -0.26  (-0.77 - 0.25) |
| **Jordan** | 75.88  (64.77 - 89.27) | 74.96  (60.64 - 91.31) | 1593.19  (1296.64 - 1967.11) | 7663.08  (6087.76 - 9476.26) | -0.17  (-0.66 - 0.32) |
| **Kuwait** | 73.2  (61.02 - 88.58) | 76.34  (59.43 - 95.07) | 1013.04  (783.17 - 1308.12) | 3884.79  (2899.07 - 5048.53) | 0.09  (-0.4 - 0.58) |
| **Lebanon** | 85.62  (72.25 - 102.13) | 88.21  (71.52 - 106.41) | 2226.56  (1866.71 - 2688.71) | 4729.13  (3837.16 - 5746.63) | 0.01  (-0.44 - 0.46) |
| **Libya** | 87.05  (74.12 - 102.45) | 75.69  (62.52 - 92.17) | 2323.56  (1924.4 - 2802.67) | 5274.54  (4234.66 - 6551.49) | -0.7  (-1.16 - -0.23) |
| **Morocco** | 122  (103.89 - 142.13) | 89.63  (75.66 - 106.75) | 22515.51  (19073.69 - 26745.18) | 31351.19  (26242.75 - 37764.84) | -1.36  (-1.76 - -0.95) |
| **Oman** | 101.48  (85.36 - 119.87) | 85.42  (69.34 - 105.61) | 1235.63  (992.74 - 1523.58) | 3764.92  (2848.32 - 4986.01) | -0.91  (-1.34 - -0.47) |
| **Palestine** | 79.53  (65 - 97.36) | 77.2  (61.51 - 96.3) | 1063.42  (845.36 - 1336.74) | 3088.13  (2411.99 - 3934.72) | 0.23  (-0.25 - 0.71) |
| **Qatar** | 98.78  (82.25 - 116.92) | 94.74  (75.8 - 116.25) | 324.12  (246.86 - 420.21) | 2997.28  (2204.48 - 3996.78) | -0.13  (-0.56 - 0.3) |
| **Saudi Arabia** | 79.18  (66.05 - 94.36) | 73.84  (59.26 - 90.96) | 8505.97  (6771.71 - 10556.88) | 27923.88  (21413.2 - 35501) | -0.27  (-0.76 - 0.22) |
| **Sudan** | 104.73  (88.95 - 122.51) | 93.12  (78.32 - 112.07) | 13645.97  (11310.81 - 16336.12) | 28317.26  (22817.49 - 35266.12) | -0.67  (-1.08 - -0.25) |
| **Syrian Arab Republic** | 39.21  (32.5 - 47.38) | 47.59  (37.15 - 59.22) | 3096.36  (2504.65 - 3841.66) | 6819.96  (5279.59 - 8577.82) | 0.29  (-0.34 - 0.92) |
| **Tunisia** | 92.07  (77.89 - 108.59) | 82.77  (67.79 - 100.78) | 5820.39  (4830.79 - 7035.16) | 10455.96  (8458.19 - 12778.61) | -0.55  (-1 - -0.1) |
| **Turkey** | 109.15  (90.02 - 132.09) | 114.77  (91.16 - 142.13) | 55428.11  (44223.42 - 67957.42) | 106354.98  (83943.05 - 131714.87) | 1.39  (1.01 - 1.77) |
| **United Arab Emirates** | 92.18  (77.64 - 108.63) | 80.16  (65.43 - 97.92) | 1134.14  (873.26 - 1472.85) | 8578.92  (6303.42 - 11283.82) | -0.67  (-1.12 - -0.22) |
| **Yemen** | 170.79  (146.01 - 198.43) | 126.9  (108.84 - 148.72) | 12557.98  (10698.96 - 14769.01) | 26221.43  (22013.06 - 31416.88) | -1.3  (-1.63 - -0.96) |
| **South Asia** | 277.71  (232.48 - 329.81) | 156.62  (130.58 - 187.05) | 2067960.9  (1737831.47 - 2452046.12) | 2520121.44  (2085275.77 - 3013097.61) | -2.31  (-2.6 - -2.02) |
| **Bangladesh** | 510.65  (433.61 - 598.94) | 114.48  (95.52 - 139.08) | 306229.54  (260587.76 - 355312.12) | 175253.42  (144388.5 - 215505.61) | -6.8  (-7.07 - -6.53) |
| **Bhutan** | 308.56  (265.67 - 358.37) | 111.16  (95.49 - 129.36) | 1252.56  (1069.69 - 1463.04) | 751.63  (637.73 - 890.5) | -4.24  (-4.55 - -3.94) |
| **India** | 256.47  (212.65 - 307.49) | 163.48  (135.7 - 195.25) | 1536086.86  (1279468.18 - 1834878.42) | 2092061.54  (1730049.92 - 2504232.24) | -1.76  (-2.05 - -1.46) |
| **Nepal** | 293.66  (252.87 - 340.21) | 136.82  (118.35 - 157.82) | 37427.26  (32213.82 - 43262.61) | 34785.94  (29952.95 - 40478.38) | -3.26  (-3.56 - -2.97) |
| **Pakistan** | 257.6  (214.72 - 308.71) | 143.2  (119.29 - 170.2) | 186964.69  (156019.56 - 221795.13) | 217268.91  (179308.04 - 260944.31) | -2.28  (-2.57 - -1.98) |
| **East Asia** | 138.67  (114.74 - 169.67) | 74.43  (61.56 - 89.61) | 1394744.71  (1141896.42 - 1702486.96) | 1489858.38  (1219467.49 - 1820689.9) | -2.44  (-2.86 - -2.02) |
| **China** | 138.17  (113.5 - 169.44) | 74.46  (61.5 - 89.8) | 1342862.06  (1094311.96 - 1644127.89) | 1440379.08  (1177194.15 - 1762007.63) | -2.42  (-2.84 - -1.99) |
| **Democratic People's Republic of Korea** | 157.12  (134.95 - 182.85) | 96.44  (82.47 - 111.38) | 28585.67  (24363.05 - 33509.9) | 30899.75  (26245.5 - 35782.88) | -1.91  (-2.27 - -1.54) |
| **Taiwan (Province of China)** | 143.23  (124.77 - 162.84) | 53.72  (44.44 - 63.77) | 23296.98  (20294.89 - 26689.17) | 18579.54  (15372.51 - 21991.34) | -3.92  (-4.34 - -3.49) |
| **Oceania** | 161.27  (140.23 - 185.31) | 129.88  (112.37 - 151.12) | 7979.91  (6759.73 - 9321.72) | 13860.34  (11737 - 16267.3) | -0.99  (-1.33 - -0.65) |
| **American Samoa** | 117.1  (99.83 - 135.48) | 94.08  (80.2 - 110.28) | 40.69  (33.9 - 48.2) | 49.59  (41.82 - 58.47) | -1.01  (-1.42 - -0.6) |
| **Cook Islands** | 93.94  (79.07 - 110.32) | 95.75  (80.54 - 113.76) | 14.79  (12.26 - 17.62) | 20.05  (16.99 - 23.64) | 0.01  (-0.43 - 0.44) |
| **Fiji** | 136.17  (118.1 - 155.44) | 101.43  (87.21 - 118.64) | 774.92  (655.08 - 901.14) | 870.73  (739.19 - 1024.01) | -1.36  (-1.74 - -0.98) |
| **Guam** | 83.73  (70.24 - 99.4) | 75.65  (60.96 - 92.62) | 97.71  (79.82 - 119.42) | 132.14  (107.02 - 160.38) | -0.57  (-1.05 - -0.09) |
| **Kiribati** | 433.17  (374.31 - 497.07) | 330.32  (286.98 - 379.81) | 261.42  (224 - 303.59) | 325.45  (282.57 - 376.26) | -1.2  (-1.41 - -1) |
| **Marshall Islands** | 218.32  (189.21 - 251.51) | 155.99  (135.29 - 180.29) | 66.24  (56.78 - 77.58) | 72.69  (62.4 - 84.46) | -1.42  (-1.72 - -1.13) |
| **Micronesia (Federated States of)** | 252.1  (218.86 - 288.32) | 157.01  (135.2 - 180.28) | 189.12  (162.59 - 219.21) | 139.57  (120.08 - 162.92) | -2.11  (-2.4 - -1.82) |
| **Nauru** | 155.06  (133.66 - 178.56) | 139.81  (120.16 - 161.16) | 10.27  (8.73 - 12.04) | 10.57  (8.83 - 12.38) | -0.55  (-0.87 - -0.22) |
| **Niue** | 112.34  (95.76 - 130.47) | 98.56  (83.27 - 116.63) | 2.42  (2.07 - 2.81) | 1.86  (1.59 - 2.18) | -0.7  (-1.1 - -0.29) |
| **Northern Mariana Islands** | 101.05  (86.06 - 117.58) | 98.94  (83.29 - 117.22) | 34.46  (27.97 - 42.1) | 47.77  (40.49 - 56.23) | -0.24  (-0.65 - 0.17) |
| **Palau** | 102.7  (88.02 - 119.89) | 100.48  (84.77 - 119.13) | 13.36  (11.06 - 15.85) | 20.16  (16.98 - 23.87) | -0.19  (-0.61 - 0.23) |
| **Papua New Guinea** | 149.3  (128.89 - 173.14) | 124.58  (106.99 - 145.65) | 4741.3  (3957.58 - 5610.14) | 9651.7  (8112.01 - 11368.21) | -0.84  (-1.19 - -0.49) |
| **Samoa** | 165.08  (141.61 - 189.94) | 140.75  (121.51 - 161.78) | 192.17  (164.07 - 224.89) | 244.05  (208.62 - 284.12) | -0.85  (-1.18 - -0.52) |
| **Solomon Islands** | 246.64  (212.79 - 284.89) | 182.4  (157.85 - 210.98) | 586.65  (501.76 - 691.1) | 906.76  (779.7 - 1054.61) | -1.29  (-1.57 - -1.01) |
| **Tokelau** | 116.64  (99.7 - 135.11) | 97.96  (82.59 - 115.06) | 1.64  (1.4 - 1.91) | 1.29  (1.09 - 1.53) | -0.85  (-1.25 - -0.45) |
| **Tonga** | 187.18  (161.7 - 214.53) | 141.85  (121.49 - 163.72) | 131.62  (112.48 - 153.49) | 123.99  (105.8 - 144.05) | -1.28  (-1.6 - -0.96) |
| **Tuvalu** | 139.22  (119.62 - 160.02) | 113.44  (96.79 - 131.77) | 11.12  (9.5 - 12.89) | 12.73  (10.81 - 14.88) | -0.97  (-1.34 - -0.6) |
| **Vanuatu** | 336.45  (291.94 - 384.91) | 247.62  (214.3 - 284.91) | 368.42  (318.36 - 425.69) | 574.59  (497.1 - 669.04) | -1.35  (-1.58 - -1.11) |
| **Southeast Asia** | 94.42  (80.73 - 110.66) | 73.83  (61.75 - 87.61) | 322786.71  (271952.17 - 380053.94) | 495038.72  (407014.44 - 594479.99) | -1.11  (-1.57 - -0.65) |
| **Cambodia** | 282.42  (245.74 - 327.7) | 159.33  (136.44 - 184.5) | 17070.92  (14804.93 - 19680.21) | 21297.31  (18145.84 - 24685.5) | -2.46  (-2.73 - -2.18) |
| **Indonesia** | 53.68  (44.13 - 64.72) | 47.88  (38.91 - 58.34) | 70783.54  (57074.96 - 86408) | 121540.93  (96367.56 - 149399.36) | -0.67  (-1.27 - -0.07) |
| **Lao People's Democratic Republic** | 298.18  (257.41 - 343.66) | 161.24  (138.71 - 185.15) | 8422.73  (7261.96 - 9680.8) | 8749.64  (7581.89 - 10082.93) | -2.62  (-2.89 - -2.35) |
| **Malaysia** | 94.9  (81.81 - 109.15) | 79.18  (67.56 - 91.68) | 10225.47  (8702.21 - 11994.35) | 22856.45  (19390.08 - 26788.77) | -1.01  (-1.44 - -0.58) |
| **Maldives** | 64.22  (54.1 - 76.22) | 65.01  (51.57 - 80.71) | 80.93  (65.88 - 98.58) | 338.41  (257.96 - 434.67) | -0.08  (-0.62 - 0.47) |
| **Mauritius** | 103  (90.17 - 117.67) | 53.55  (45.16 - 63.05) | 919.47  (802.27 - 1061.19) | 858.19  (727.23 - 1002.19) | -2.88  (-3.36 - -2.4) |
| **Myanmar** | 215.16  (186.67 - 248.1) | 92.66  (79.47 - 107.83) | 64954.02  (56074.9 - 74608.74) | 47519.84  (40770.22 - 55556.58) | -3.49  (-3.83 - -3.15) |
| **Philippines** | 220.14  (181.68 - 264.72) | 159.89  (132.44 - 193.71) | 92221.6  (76104.52 - 110478.45) | 146851.43  (120835.52 - 177726.16) | -1.21  (-1.51 - -0.91) |
| **Seychelles** | 268.3  (232.86 - 309.63) | 158.42  (136.15 - 181.96) | 161.46  (140.57 - 185.76) | 180.66  (154.36 - 208.62) | -2.2  (-2.48 - -1.92) |
| **Sri Lanka** | 44.18  (35.54 - 53.96) | 51.09  (39.83 - 64.26) | 6704.58  (5340.07 - 8390.2) | 12446.4  (9736.59 - 15598.95) | 0.48  (-0.15 - 1.12) |
| **Thailand** | 49.7  (41.08 - 59.81) | 60.21  (49.5 - 72.37) | 24750.76  (20113.73 - 30496.76) | 53942.95  (44278.58 - 64553.4) | 0.82  (0.24 - 1.4) |
| **Timor-Leste** | 241.04  (209.18 - 277.37) | 140.91  (121.95 - 162.98) | 1119.44  (964.65 - 1296.06) | 1348.68  (1155.52 - 1564.93) | -2.28  (-2.57 - -1.98) |
| **Viet Nam** | 49.34  (40.93 - 59.34) | 54.39  (43.59 - 66.91) | 24942.75  (20236.49 - 30746.23) | 56459.28  (44589.99 - 70172.95) | 0.27  (-0.33 - 0.87) |
| **Central Sub-Saharan Africa** | 118.68  (102.38 - 137.11) | 116.55  (99.89 - 135.38) | 40708.15  (34501.8 - 47693.24) | 97137.75  (81816.84 - 115774.3) | -0.12  (-0.5 - 0.26) |
| **Angola** | 132.58  (114.73 - 153.24) | 119.28  (103.27 - 138.23) | 8256.68  (7026.14 - 9682.23) | 21088.77  (17585.13 - 25318.95) | -0.47  (-0.83 - -0.1) |
| **Central African Republic** | 181.11  (155.81 - 209.25) | 173.85  (150.15 - 201.1) | 3230.24  (2744.97 - 3757.61) | 6026.55  (5120.38 - 7055.02) | -0.16  (-0.47 - 0.14) |
| **Congo** | 108.95  (93.98 - 126.19) | 92.13  (78.69 - 107.98) | 1738.98  (1467.3 - 2046.06) | 3499.41  (2904.25 - 4198.02) | -0.69  (-1.1 - -0.27) |
| **Democratic Republic of the Congo** | 111.43  (95.4 - 128.77) | 115.48  (98.73 - 134.76) | 26409.36  (22223.32 - 31046.33) | 64593.64  (54059.57 - 77033.48) | 0.08  (-0.3 - 0.47) |
| **Equatorial Guinea** | 149.26  (127.76 - 172.68) | 86.47  (73.39 - 102.74) | 401.32  (341.46 - 468.05) | 813.9  (645.59 - 1024.59) | -2.28  (-2.67 - -1.88) |
| **Gabon** | 96.25  (82.92 - 111.96) | 80.12  (68 - 94.47) | 671.56  (568.7 - 794.98) | 1115.48  (920.72 - 1348.89) | -0.78  (-1.23 - -0.34) |
| **Eastern Sub-Saharan Africa** | 124.83  (106.47 - 145.6) | 103.87  (88.9 - 121.26) | 155761.47  (129351.75 - 186016.29) | 298069.15  (247370.95 - 359430.8) | -0.87  (-1.25 - -0.47) |
| **Burundi** | 157.08  (134.5 - 182.5) | 140.58  (121.36 - 162.3) | 5994.65  (5005.48 - 7135.21) | 10968.13  (9268.96 - 12975.49) | -0.51  (-0.86 - -0.16) |
| **Comoros** | 132.1  (113.56 - 152.45) | 102.8  (88.12 - 120.05) | 428.18  (362.4 - 502.55) | 643.44  (543.03 - 760.75) | -1.06  (-1.44 - -0.67) |
| **Djibouti** | 117.33  (100.43 - 135.61) | 89.33  (75.71 - 104.99) | 362.32  (299.83 - 435.13) | 877.54  (730.22 - 1052.02) | -1.18  (-1.58 - -0.77) |
| **Eritrea** | 141.64  (121.84 - 163.76) | 127  (109.81 - 146.7) | 2757.54  (2309.93 - 3272.99) | 6123.46  (5165.77 - 7200.88) | -0.59  (-0.94 - -0.24) |
| **Ethiopia** | 147.65  (122.6 - 175.39) | 89.19  (73.9 - 106.97) | 50458.23  (40887.84 - 61491.16) | 67853.41  (54432.04 - 83926.35) | -2.14  (-2.52 - -1.75) |
| **Kenya** | 114.54  (95.82 - 137.04) | 99.26  (82.39 - 118.63) | 16183.4  (13133.8 - 19730.81) | 37486.52  (30235.32 - 45847.25) | -0.8  (-1.2 - -0.39) |
| **Madagascar** | 99.76  (85.71 - 115.42) | 103.62  (88.62 - 120.91) | 8590.62  (7223.51 - 10172.54) | 20256.6  (16741.02 - 24194.02) | 0.01  (-0.41 - 0.43) |
| **Malawi** | 114.11  (97.91 - 131.72) | 125.4  (107.74 - 145.13) | 7135.47  (6020.78 - 8482.39) | 15603.29  (13103.81 - 18604.22) | 0.2  (-0.18 - 0.58) |
| **Mozambique** | 115.9  (99.21 - 134.31) | 130.58  (111.5 - 152.61) | 10218.99  (8621.97 - 12062.48) | 25389.99  (21186.91 - 30299.4) | 0.43  (0.05 - 0.82) |
| **Rwanda** | 220.31  (189.36 - 254.46) | 153.22  (132.46 - 175.89) | 10330.51  (8748.85 - 12162.22) | 13654.25  (11578.12 - 15961.25) | -1.6  (-1.9 - -1.3) |
| **Somalia** | 130.33  (110.71 - 150.96) | 133.49  (113.31 - 157.53) | 5823.7  (4893.42 - 6848.08) | 17439.34  (14427.97 - 21096.16) | 0.02  (-0.34 - 0.38) |
| **South Sudan** | 98.06  (83.79 - 114.12) | 92.99  (79.29 - 109.18) | 3898.99  (3246.05 - 4629.44) | 5852.79  (4861.57 - 7086.76) | -0.24  (-0.67 - 0.19) |
| **Uganda** | 121.39  (103.8 - 142.44) | 115.72  (99.46 - 134.36) | 12803.91  (10631.75 - 15328.32) | 29623.31  (24596.3 - 35780.12) | -0.4  (-0.78 - -0.02) |
| **United Republic of Tanzania** | 81.26  (69.07 - 95.25) | 83.34  (70.47 - 99) | 14098.18  (11655.1 - 17198.39) | 34053.25  (27891.45 - 41537.41) | -0.05  (-0.52 - 0.42) |
| **Zambia** | 126.33  (108.77 - 145.26) | 92.05  (78.93 - 108.01) | 6562.48  (5492.58 - 7768.78) | 12005.54  (9883.21 - 14596.15) | -1.36  (-1.75 - -0.97) |
| **Southern Sub-Saharan Africa** | 84.6  (71.07 - 99.98) | 82.94  (69.24 - 98.05) | 32909.76  (27125.8 - 39911.73) | 57826.8  (47764.23 - 69640.81) | -0.1  (-0.55 - 0.35) |
| **Botswana** | 142.59  (121.83 - 166.65) | 100.93  (86.32 - 118.52) | 1166.49  (985 - 1384.6) | 1922.36  (1613.3 - 2299.45) | -1.25  (-1.6 - -0.9) |
| **Eswatini** | 97.54  (83.52 - 113.75) | 97.25  (83.91 - 112.47) | 469.85  (387.78 - 570.1) | 838.1  (696.18 - 1000.44) | -0.04  (-0.45 - 0.36) |
| **Lesotho** | 137.94  (118.46 - 160.22) | 152.94  (132.35 - 176.37) | 1707.15  (1462.18 - 1992.9) | 2660.08  (2269.81 - 3094.21) | 0.35  (0.01 - 0.68) |
| **Namibia** | 118.69  (101.52 - 137.4) | 103.49  (88.68 - 120.65) | 1115.5  (946.19 - 1323.76) | 1893.57  (1589.45 - 2261.84) | -0.63  (-1.02 - -0.24) |
| **South Africa** | 75.1  (61.79 - 90.25) | 74.13  (60.75 - 88.96) | 21635.11  (17646.38 - 26456.92) | 38693.23  (31362.63 - 47046.88) | -0.04  (-0.51 - 0.44) |
| **Zimbabwe** | 102.63  (88.36 - 119.81) | 106.81  (92.16 - 124.36) | 6815.66  (5690.12 - 8203.17) | 11819.46  (9916.77 - 13960.11) | 0.04  (-0.37 - 0.46) |
| **Western Sub-Saharan Africa** | 140.81  (120.52 - 164.14) | 132.26  (112.98 - 154.28) | 204168.04  (170637.3 - 243160.24) | 461063.06  (379645.7 - 552950.64) | -0.63  (-0.98 - -0.28) |
| **Benin** | 133.86  (115.44 - 154.71) | 120.06  (103.03 - 139.19) | 4529.96  (3799.99 - 5396.35) | 10730.48  (8829.7 - 12821.03) | -0.67  (-1.04 - -0.3) |
| **Burkina Faso** | 154.82  (133.34 - 179.48) | 168.44  (145.07 - 194.78) | 10692.74  (8996.36 - 12654.69) | 27468.63  (23105.36 - 32610.98) | 0.27  (-0.05 - 0.6) |
| **Cabo Verde** | 130.37  (112.71 - 150.01) | 77.41  (63.2 - 94.91) | 375.06  (315.03 - 443.44) | 438.09  (348.01 - 547.04) | -2.32  (-2.76 - -1.88) |
| **Cameroon** | 145.51  (126.23 - 166.52) | 118.33  (102.61 - 137.9) | 10866  (9209.54 - 12783.62) | 25990.23  (21428.41 - 31344.04) | -1.03  (-1.38 - -0.67) |
| **Chad** | 181.14  (155.89 - 208.5) | 159.82  (138.17 - 184.82) | 7881.79  (6648.66 - 9271.32) | 18375.27  (15282.55 - 22137.57) | -0.65  (-0.95 - -0.35) |
| **Cote d'Ivoire** | 148.21  (128.85 - 170.05) | 135.62  (117.33 - 156.57) | 12790.08  (10722.44 - 15107.73) | 26768.5  (22491.55 - 31597.82) | -0.63  (-0.97 - -0.29) |
| **Gambia** | 159.44  (137.57 - 183.97) | 121.81  (104.77 - 140.42) | 1063.72  (890.39 - 1254.02) | 2057.07  (1701.88 - 2441.29) | -1.18  (-1.52 - -0.84) |
| **Ghana** | 94.5  (81.1 - 109.62) | 115.93  (98.74 - 135.99) | 10768.82  (8870.92 - 12894.64) | 31613.93  (26235.5 - 38340.72) | 0.84  (0.43 - 1.26) |
| **Guinea** | 155.28  (134.44 - 179.09) | 131.49  (112.57 - 152.61) | 7456.69  (6343.05 - 8842.52) | 12444.51  (10357.33 - 14843.19) | -0.87  (-1.21 - -0.52) |
| **Guinea-Bissau** | 207.03  (179.45 - 238.34) | 158.04  (137.49 - 181.41) | 1514.97  (1279.05 - 1783.1) | 2321.35  (1964.16 - 2730.97) | -1.16  (-1.46 - -0.86) |
| **Liberia** | 132.08  (113.05 - 153.82) | 113.9  (96.11 - 134.14) | 2186.08  (1789.45 - 2658.77) | 4405.48  (3586.43 - 5336.02) | -0.9  (-1.27 - -0.53) |
| **Mali** | 154.17  (131.76 - 178.45) | 148.83  (129.21 - 172.43) | 10273.22  (8594.42 - 12208.1) | 23192.47  (19364.07 - 27524.5) | -2.24  (-2.54 - -1.93) |
| **Mauritania** | 135.56  (117.32 - 156.16) | 101.94  (85.7 - 120.16) | 2091.35  (1767.39 - 2462.24) | 3320.72  (2693.05 - 4016.89) | -1.32  (-1.69 - -0.94) |
| **Niger** | 189.91  (163.44 - 220.15) | 165.49  (143.45 - 190.74) | 10842.31  (9046.21 - 13043.65) | 24645.1  (20606.37 - 29401.06) | -2.74  (-3.02 - -2.46) |
| **Nigeria** | 135.85  (113.21 - 160.62) | 128.38  (107.05 - 152.14) | 94822.81  (77530.02 - 114395.04) | 215283.64  (173813.91 - 263217.69) | -0.33  (-0.69 - 0.02) |
| **Sao Tome and Principe** | 101.53  (86.97 - 117.78) | 79.74  (66.35 - 96.21) | 99.39  (81.58 - 119) | 142.7  (113.78 - 175.82) | -1.16  (-1.59 - -0.74) |
| **Senegal** | 151.27  (131.52 - 174.13) | 120.56  (103.89 - 140.27) | 8230.64  (6905.89 - 9787.88) | 14254.97  (12042.62 - 16821.46) | -1.08  (-1.43 - -0.72) |
| **Sierra Leone** | 137.09  (117.22 - 159.51) | 145.96  (126.35 - 169.2) | 4055.34  (3403.66 - 4838.79) | 9520.2  (7967.31 - 11252.08) | 0.22  (-0.13 - 0.57) |
| **Togo** | 144.06  (125.36 - 166.14) | 135.89  (117.06 - 157.58) | 3620.22  (3019.15 - 4315.47) | 8083.27  (6808.42 - 9520.91) | -0.39  (-0.74 - -0.04) |
| **High SDI^§^** | 102.24  (86.55 - 120.43) | 80.98  (68.19 - 95.81) | 1009058.38  (858166.74 - 1187537.39) | 1210511.54  (1034476.18 - 1425082.19) | -0.7  (-1.14 - -0.26) |
| **High-middle SDI** | 119.5  (99.85 - 143.43) | 84.04  (69.88 - 100.29) | 1354205.11  (1126370.6 - 1623342.52) | 1550525.63  (1285170.95 - 1857861.05) | -1.28  (-1.7 - -0.86) |
| **Middle SDI** | 119.12  (98.74 - 143.46) | 78.19  (65.27 - 92.95) | 1516929.93  (1252830.09 - 1823350.97) | 2001993.04  (1652545.26 - 2392497.12) | -1.69  (-2.12 - -1.26) |
| **Low-middle SDI** | 240.98  (202.56 - 285) | 140.5  (117.9 - 166.25) | 1860537.39  (1569452.97 - 2193244.43) | 2195504.43  (1832149.87 - 2613421.94) | -2.2  (-2.51 - -1.9) |
| **Low SDI** | 201.41  (171.14 - 236.01) | 145.35  (123.83 - 169.9) | 691233.66  (585194.43 - 810240.43) | 1128657.58  (952310.77 - 1334547.17) | -1.41  (-1.72 - -1.1) |

*Age-standardized rate (per 100,000 population)

¶ Estimated annual percentage change

§ Socio-demographic index

**Additional file 1:**

**Table S2.** **PUD incidence in 1990 and 2019 for both sexes and estimated annual percentage change in age-standardized rates by location**

| **Characteristic** | **Rate^*^** | | **Number** | | **EAPC^¶^** |
| --- | --- | --- | --- | --- | --- |
|  | **No (95% UI)** | | **No (95% UI)** | | **No (95% CI)** |
|  | **1990** | **2019** | **1990** | **2019** | **1990-2019** |
| **Global** | 63.84  (54.09 - 75.54) | 44.26  (37.32 - 51.87) | 2815018.54  (2362079.56 - 3301982.98) | 3591469.27  (3031287.79 - 4217644.86) | -1.45  (-2.02 - -0.87) |
| **Central Asia** | 53  (45.66 - 61.01) | 40.27  (35.14 - 45.05) | 29105.66  (24990.84 - 33424.03) | 35398.33  (30667.16 - 40017.32) | -1.18  (-1.77 - -0.57) |
| **Armenia** | 64.37  (54.98 - 75.99) | 47.57  (41.55 - 53.71) | 1984.27  (1686.32 - 2347.25) | 1819.76  (1576.53 - 2068.05) | -1.29  (-1.87 - -0.72) |
| **Azerbaijan** | 49.05  (41.42 - 56.76) | 32.5  (27.53 - 37.5) | 3003.3  (2505.18 - 3483.56) | 3559.51  (2999.99 - 4153.44) | -1.88  (-2.52 - -1.23) |
| **Georgia** | 42.44  (36.64 - 48.68) | 46.24  (39.98 - 53.03) | 2552.2  (2192.88 - 2959.77) | 2200.14  (1913.75 - 2502.89) | 0.42  (-0.24 - 1.08) |
| **Kazakhstan** | 58.45  (50.05 - 67.73) | 39.26  (34.16 - 44.48) | 8384.48  (7173.65 - 9710.17) | 7406.49  (6404.56 - 8411.51) | -1.65  (-2.24 - -1.06) |
| **Kyrgyzstan** | 53.21  (45.67 - 61.48) | 29.42  (25.25 - 33.83) | 1833.07  (1566.8 - 2127.33) | 1730.17  (1463.27 - 2020.7) | -2.38  (-3.05 - -1.71) |
| **Mongolia** | 64.58  (55.75 - 74.7) | 61.89  (53.55 - 71.13) | 845.21  (730.3 - 973.94) | 1750.2  (1501.05 - 2011.65) | -0.07  (-0.58 - 0.45) |
| **Tajikistan** | 57.91  (49.69 - 68.05) | 40.45  (34.93 - 45.95) | 2096.66  (1773.24 - 2449.47) | 2946.82  (2490.73 - 3374.77) | -1.58  (-2.15 - -1) |
| **Turkmenistan** | 43.56  (37.08 - 50.29) | 41.09  (35.11 - 47.3) | 1144.89  (959.41 - 1325.06) | 2039.85  (1723.2 - 2360.18) | -0.24  (-0.87 - 0.4) |
| **Uzbekistan** | 50.54  (43.54 - 57.88) | 41.14  (35.78 - 46.3) | 7261.58  (6140.59 - 8310.54) | 11945.38  (10164.12 - 13641.47) | -0.99  (-1.57 - -0.41) |
| **Central Europe** | 49.16  (42.58 - 55.78) | 44.12  (37.94 - 50.44) | 69494.77  (60087.82 - 79144.58) | 75532.21  (65172.31 - 86820.87) | -0.27  (-0.88 - 0.34) |
| **Albania** | 33.27  (28.04 - 38.53) | 30.78  (24.56 - 37.54) | 858.56  (710.31 - 1014.73) | 1033.45  (836.09 - 1245.23) | -0.43  (-1.19 - 0.33) |
| **Bosnia and Herzegovina** | 49.44  (42.04 - 57.96) | 33.95  (28.4 - 39.35) | 2177  (1844.83 - 2549.35) | 1618.84  (1372.49 - 1868.7) | -1.46  (-2.13 - -0.79) |
| **Bulgaria** | 44.66  (38.97 - 50.72) | 35.22  (30.29 - 40.73) | 5070.15  (4406.03 - 5761.94) | 3815.45  (3290.09 - 4380.68) | -0.93  (-1.6 - -0.26) |
| **Croatia** | 49.96  (43.7 - 56.6) | 37.27  (31.67 - 42.73) | 3045.79  (2651.78 - 3461.02) | 2541.62  (2171.25 - 2926.12) | -1.15  (-1.76 - -0.54) |
| **Czechia** | 45.22  (39.19 - 51) | 35.01  (29.8 - 40.16) | 5836.95  (5020.25 - 6607.49) | 5761.2  (4942.63 - 6581.79) | -1.03  (-1.67 - -0.38) |
| **Hungary** | 61.41  (53.21 - 70.03) | 46.23  (39.6 - 52.32) | 8267.01  (7094.66 - 9429.53) | 7093.64  (6060.54 - 8079.53) | -1.16  (-1.72 - -0.6) |
| **Montenegro** | 40.84  (35.34 - 47.12) | 34.51  (29.08 - 40.1) | 262.18  (225.67 - 302.72) | 284.54  (242.67 - 329.32) | -0.65  (-1.32 - 0.03) |
| **North Macedonia** | 40.84  (35.34 - 46.72) | 32.09  (26.58 - 37.73) | 805.14  (691 - 923.33) | 896.13  (748.13 - 1054.22) | -1.06  (-1.75 - -0.37) |
| **Poland** | 54.92  (45.5 - 66.37) | 58.89  (50.49 - 68.74) | 23195.56  (19171.1 - 28272.2) | 33795.07  (28744.68 - 39995.72) | 0.55  (-0.01 - 1.12) |
| **Romania** | 38.61  (33.1 - 44.27) | 30.48  (25.53 - 35.51) | 10323.74  (8835.96 - 11852.24) | 8229.98  (6968.66 - 9526.2) | -0.47  (-1.16 - 0.23) |
| **Serbia** | 48.64  (42.02 - 55.72) | 44.49  (38.29 - 50.61) | 5446.23  (4685.11 - 6322.46) | 6046.71  (5115.82 - 6922.89) | -0.09  (-0.69 - 0.51) |
| **Slovakia** | 52.42  (45.39 - 59.55) | 44.21  (37.67 - 50.69) | 3017.72  (2604.96 - 3422.74) | 3407.41  (2938.94 - 3906.46) | -0.8  (-1.38 - -0.22) |
| **Slovenia** | 50.33  (43.33 - 57.6) | 31.57  (25.93 - 37.03) | 1188.73  (1021.38 - 1361.93) | 1008.17  (848.17 - 1168.59) | -2  (-2.65 - -1.36) |
| **Eastern Europe** | 69.71  (57.47 - 83.01) | 65.19  (53.67 - 77.32) | 180891.94  (148493.57 - 217045.68) | 181578.25  (150357.39 - 219895.96) | -0.17  (-0.67 - 0.35) |
| **Belarus** | 59.76  (51.17 - 69.27) | 50.79  (42.59 - 58.69) | 7125.36  (6098.55 - 8267.98) | 6173.74  (5281.33 - 7135.2) | -0.61  (-1.18 - -0.04) |
| **Estonia** | 58.63  (50.78 - 68.36) | 48.35  (40.81 - 56.04) | 1088.59  (935.97 - 1270.24) | 859.58  (737.05 - 980.15) | -0.33  (-0.88 - 0.22) |
| **Latvia** | 61.91  (52.92 - 70.75) | 54.11  (46 - 62.49) | 1969.72  (1683.54 - 2271.46) | 1467.52  (1262.68 - 1676.57) | -0.04  (-0.57 - 0.5) |
| **Lithuania** | 62.81  (53.77 - 71.62) | 72.08  (61 - 82.02) | 2591.1  (2227.3 - 2951.23) | 2928.02  (2454.08 - 3334.68) | 0.64  (0.14 - 1.14) |
| **Republic of Moldova** | 70.56  (60.26 - 82.41) | 61.19  (52.39 - 69.84) | 3214.16  (2733.33 - 3752.73) | 2921.82  (2501.89 - 3380.69) | -0.46  (-0.98 - 0.06) |
| **Russian Federation** | 67.31  (55.01 - 80.67) | 66.22  (54.68 - 78.9) | 115186.14  (93609.93 - 138677.65) | 129491.07  (106835.16 - 157963.42) | 0.04  (-0.47 - 0.55) |
| **Ukraine** | 79.06  (64.65 - 95.82) | 66.3  (53.36 - 80.21) | 49716.86  (40642.2 - 60672.55) | 37736.51  (30569.13 - 46069.44) | -0.67  (-1.17 - -0.17) |
| **Australasia** | 32.47  (27.46 - 37.35) | 17.81  (14.79 - 21.15) | 7461.51  (6274.92 - 8610.17) | 7316.79  (6154.4 - 8705.39) | -2.5  (-3.36 - -1.64) |
| **Australia** | 31.93  (26.83 - 36.64) | 17.36  (14.31 - 20.72) | 6108.51  (5095.16 - 7024.18) | 5988.45  (5029.05 - 7137.56) | -2.57  (-3.43 - -1.69) |
| **New Zealand** | 35.12  (28.58 - 43.2) | 20.19  (17.11 - 23.67) | 1353  (1088.5 - 1670.44) | 1328.33  (1121.01 - 1579.18) | -2.21  (-3 - -1.41) |
| **High-income Asia Pacific** | 52.72  (44.33 - 62.17) | 46.71  (38.54 - 55.48) | 102381.76  (85730.18 - 122252.63) | 136723.59  (113849.69 - 164400.32) | -0.19  (-0.79 - 0.41) |
| **Brunei Darussalam** | 86.34  (74.01 - 102.77) | 41.26  (34.9 - 47.86) | 113.02  (96.2 - 132.03) | 163.99  (132.58 - 196.55) | -3.19  (-3.73 - -2.65) |
| **Japan** | 52.67  (43.97 - 62.91) | 49.89  (41.48 - 59.03) | 81275.15  (67707.06 - 97851.49) | 105459.74  (87931.63 - 127477.9) | 0.17  (-0.42 - 0.77) |
| **Republic of Korea** | 55.3  (47.76 - 64.02) | 39.53  (31.86 - 47.54) | 19524.71  (16589.79 - 22977.76) | 28352.03  (23010.84 - 34534.21) | -1.52  (-2.13 - -0.9) |
| **Singapore** | 55  (46.83 - 62.9) | 37.97  (30.59 - 46.22) | 1468.87  (1246.76 - 1698.94) | 2747.83  (2160.18 - 3409.86) | -1.46  (-2.07 - -0.85) |
| **High-income North America** | 54.91  (45.57 - 66.21) | 42.54  (36.19 - 49.77) | 186171.05  (153781.45 - 224413.25) | 229019.38  (195793.99 - 270045.13) | -0.66  (-1.27 - -0.05) |
| **Canada** | 47.11  (39.94 - 53.87) | 33.34  (27.64 - 39.39) | 14921.82  (12655.74 - 17143.64) | 17873.39  (15049.81 - 20980) | -1.44  (-2.11 - -0.77) |
| **Greenland** | 130.9  (110.11 - 155.76) | 81.85  (69.81 - 96.27) | 50.42  (43.45 - 59.51) | 54.44  (45.91 - 65.63) | -1.93  (-2.34 - -1.53) |
| **United States of America** | 55.75  (46.03 - 67.57) | 43.67  (37.12 - 51.09) | 171194.56  (140499.77 - 208354.64) | 211087.91  (178891.23 - 249756.82) | -0.58  (-1.19 - 0.03) |
| **Southern Latin America** | 19.48  (16.8 - 22.08) | 16.23  (13.67 - 18.73) | 9104.39  (7839.56 - 10356.95) | 12595.84  (10682.37 - 14492.94) | -0.8  (-1.77 - 0.18) |
| **Argentina** | 18.58  (15.94 - 21.23) | 16.78  (14.21 - 19.23) | 5954.61  (5107.34 - 6814.59) | 8540.88  (7262.12 - 9743.51) | -0.44  (-1.44 - 0.56) |
| **Chile** | 22.1  (18.95 - 25.1) | 15.18  (12.66 - 17.84) | 2407.94  (2049.82 - 2756.49) | 3362.35  (2819.51 - 3938.69) | -1.57  (-2.48 - -0.66) |
| **Uruguay** | 20.41  (17.49 - 23.48) | 15.35  (12.93 - 18.05) | 741.48  (634.36 - 851.73) | 691.97  (588.45 - 798.17) | -1.17  (-2.15 - -0.18) |
| **Western Europe** | 26.83  (23.17 - 30.9) | 17.94  (14.86 - 21.17) | 141598.76  (122409.76 - 163120.15) | 124178.06  (104988.04 - 146343.45) | -1.49  (-2.35 - -0.62) |
| **Andorra** | 22.76  (19.41 - 26.58) | 17.19  (14.21 - 20.38) | 12.63  (10.65 - 14.94) | 21.88  (18.06 - 26.3) | -1.24  (-2.17 - -0.31) |
| **Austria** | 40.33  (34.55 - 46.36) | 23.73  (19.29 - 28.31) | 4127.89  (3535.31 - 4730.57) | 3030.83  (2496.64 - 3590.82) | -2.25  (-2.98 - -1.52) |
| **Belgium** | 26.41  (22.77 - 29.99) | 19.14  (15.63 - 23.06) | 3644.84  (3152.13 - 4139.64) | 3252.3  (2720.1 - 3822.2) | -0.88  (-1.7 - -0.04) |
| **Cyprus** | 18.36  (16.04 - 20.83) | 12.18  (10.18 - 14.41) | 128.43  (110.68 - 148.12) | 210.8  (175.89 - 251.3) | -1.63  (-2.69 - -0.57) |
| **Denmark** | 36.88  (31.69 - 42.02) | 23.97  (20.39 - 27.44) | 2789.21  (2388.25 - 3182.37) | 2302.66  (1979.93 - 2641.39) | -1.58  (-2.29 - -0.87) |
| **Finland** | 31.26  (26.74 - 35.7) | 19  (15.96 - 22.31) | 2074.47  (1764.23 - 2371.01) | 1654.97  (1417.94 - 1911.37) | -2.05  (-2.86 - -1.23) |
| **France** | 22.93  (19.84 - 26.15) | 14.72  (11.84 - 17.75) | 17558.66  (15275.4 - 19938.42) | 14365.28  (11976.82 - 17049.8) | -2.04  (-3.02 - -1.05) |
| **Germany** | 25.82  (22.33 - 29.48) | 19.77  (16.4 - 23.24) | 29430.07  (25518.03 - 33649.44) | 28102.22  (23832.91 - 32474.38) | -0.53  (-1.37 - 0.3) |
| **Greece** | 17.87  (15.19 - 20.67) | 20.01  (17.01 - 23) | 2433.51  (2073.55 - 2792.21) | 3886.6  (3355.28 - 4430.09) | 0.38  (-0.57 - 1.34) |
| **Iceland** | 19.79  (16.9 - 22.87) | 13.46  (11.02 - 16.15) | 54.09  (46.53 - 62.52) | 62.03  (51.49 - 73.51) | -1.18  (-2.17 - -0.18) |
| **Ireland** | 31.49  (27.14 - 36.02) | 16.91  (14.02 - 20.04) | 1243.34  (1067.61 - 1435.4) | 1101.53  (926.25 - 1297.36) | -2.67  (-3.49 - -1.85) |
| **Israel** | 14.45  (12.51 - 16.4) | 7.56  (6.1 - 9.07) | 683.97  (588.98 - 781.41) | 788.66  (642.58 - 947.25) | -2.93  (-4.18 - -1.66) |
| **Italy** | 24.7  (20.44 - 29.86) | 17.73  (14.7 - 21.17) | 19629.78  (16196.58 - 23877.86) | 19234.01  (16010.02 - 23294.68) | -1.17  (-2.04 - -0.29) |
| **Luxembourg** | 24.6  (20.87 - 28.13) | 18.67  (15.14 - 22.66) | 123.32  (104.82 - 140.96) | 158.77  (129.15 - 192.57) | -1.32  (-2.16 - -0.47) |
| **Malta** | 20.14  (17.18 - 22.94) | 12.09  (10.14 - 14.25) | 84.5  (71.91 - 96.59) | 89.96  (76.35 - 104.36) | -2.01  (-3.04 - -0.98) |
| **Monaco** | 13.88  (11.22 - 16.82) | 14.44  (11.52 - 17.88) | 6.94  (5.75 - 8.27) | 8.77  (7.13 - 10.71) | 0.07  (-1.05 - 1.2) |
| **Netherlands** | 24.95  (21.47 - 28.28) | 15.41  (12.41 - 18.7) | 4765.2  (4081.93 - 5394.37) | 3937.62  (3249.51 - 4701.01) | -2.08  (-3 - -1.14) |
| **Norway** | 40.2  (33.4 - 48.39) | 34.95  (28.3 - 42.22) | 2371.14  (1963.76 - 2880.66) | 2581.12  (2131.25 - 3137.84) | 1.08  (0.45 - 1.71) |
| **Portugal** | 21.66  (18.81 - 24.52) | 13.07  (10.91 - 15.47) | 2791.29  (2411.7 - 3166.24) | 2384.58  (2036.95 - 2786.54) | -2.35  (-3.35 - -1.35) |
| **San Marino** | 16.43  (13.69 - 19.37) | 15.29  (12.39 - 18.58) | 4.86  (4.1 - 5.7) | 7.51  (6.23 - 9.06) | -0.37  (-1.42 - 0.7) |
| **Spain** | 23.2  (20.18 - 26.16) | 14.66  (11.79 - 18.06) | 11542.27  (10077.66 - 12995.5) | 10345.04  (8485.22 - 12526.32) | -2.12  (-3.09 - -1.13) |
| **Sweden** | 31.21  (25.65 - 37.82) | 22.64  (18.52 - 27.35) | 4062.75  (3333.21 - 4983.46) | 3509.37  (2886.58 - 4262.97) | -1.32  (-2.1 - -0.53) |
| **Switzerland** | 18.71  (15.89 - 21.6) | 16.05  (13.02 - 19.48) | 1760.43  (1505.1 - 2019.59) | 2160.25  (1790.55 - 2563.64) | -0.67  (-1.62 - 0.29) |
| **United Kingdom** | 36.14  (30.05 - 43.61) | 20.33  (17.09 - 24.12) | 30157.59  (25003.12 - 36730.66) | 20873.02  (17666.05 - 24851.93) | -2.35  (-3.13 - -1.56) |
| **Andean Latin America** | 32.56  (28.65 - 36.8) | 20.96  (18.21 - 23.71) | 8048.09  (6941.59 - 9134.24) | 12367.29  (10675.4 - 14070.13) | -1.71  (-2.5 - -0.9) |
| **Bolivia (Plurinational State of)** | 43.15  (37.52 - 49.51) | 26.21  (22.87 - 29.75) | 1620.61  (1396.2 - 1855.99) | 2479.48  (2150.11 - 2835.23) | -2.11  (-2.82 - -1.41) |
| **Ecuador** | 35.16  (31.26 - 39.49) | 22.11  (19.71 - 24.65) | 2215.87  (1936.26 - 2507.16) | 3420.82  (3034.62 - 3829.3) | -1.66  (-2.42 - -0.9) |
| **Peru** | 28.82  (25.13 - 32.78) | 19.28  (16.43 - 22.42) | 4211.61  (3593.2 - 4804.76) | 6467  (5464.91 - 7556.79) | -1.56  (-2.41 - -0.71) |
| **Caribbean** | 33.99  (29.82 - 38.2) | 26.19  (22.62 - 29.91) | 9766.51  (8511.4 - 11022.57) | 13241.57  (11452.38 - 15113.5) | -1.14  (-1.9 - -0.38) |
| **Antigua and Barbuda** | 21.93  (18.99 - 25) | 18.63  (15.51 - 22.06) | 12.13  (10.43 - 13.94) | 18.75  (15.48 - 22.2) | -0.79  (-1.72 - 0.15) |
| **Bahamas** | 25.83  (22.5 - 29.2) | 21.64  (18.6 - 24.6) | 49.4  (42.45 - 56.4) | 88.45  (75.51 - 101.09) | -0.87  (-1.71 - -0.02) |
| **Barbados** | 28.4  (24.96 - 32.06) | 20.8  (17.91 - 23.65) | 78.76  (69.3 - 89.16) | 85.65  (75.43 - 96.44) | -1.37  (-2.2 - -0.53) |
| **Belize** | 23.8  (20.66 - 26.98) | 20.09  (17 - 23.39) | 28.02  (23.75 - 32.48) | 71.67  (58.91 - 85.26) | -0.83  (-1.71 - 0.06) |
| **Bermuda** | 27.02  (23.33 - 30.62) | 20.23  (16.91 - 23.56) | 17.33  (14.97 - 19.69) | 19.8  (16.91 - 22.73) | -1.33  (-2.19 - -0.47) |
| **Cuba** | 30.43  (26.37 - 34.16) | 22.07  (18.71 - 25.74) | 3224.46  (2786.43 - 3628.43) | 3499.1  (3015.43 - 4010.76) | -1.39  (-2.21 - -0.56) |
| **Dominica** | 26.26  (23.05 - 29.86) | 20.62  (17.57 - 23.66) | 17.96  (15.69 - 20.43) | 16.75  (14.53 - 19.12) | -1.07  (-1.93 - -0.21) |
| **Dominican Republic** | 27.2  (23.84 - 30.86) | 24.61  (21.28 - 28.23) | 1321.3  (1129.75 - 1525.25) | 2500.02  (2133.77 - 2882.96) | -0.46  (-1.28 - 0.36) |
| **Grenada** | 31.22  (27.4 - 35.53) | 23.51  (20.33 - 26.91) | 22.29  (19.58 - 25.24) | 26.21  (22.66 - 29.93) | -1.27  (-2.06 - -0.46) |
| **Guyana** | 53.56  (47 - 60.69) | 36  (31.74 - 40.83) | 258.39  (223.56 - 293.52) | 249.15  (217.92 - 282.3) | -1.52  (-2.15 - -0.88) |
| **Haiti** | 61.68  (53.39 - 71.58) | 47.99  (41.65 - 55.44) | 2471.02  (2117.19 - 2873.79) | 4018.67  (3458.95 - 4656.09) | -1.07  (-1.62 - -0.52) |
| **Jamaica** | 31.76  (27.65 - 35.89) | 22.84  (19.63 - 26.33) | 588.98  (510.76 - 665.15) | 689.43  (592.42 - 794.28) | -1.52  (-2.31 - -0.73) |
| **Puerto Rico** | 22.64  (19.6 - 25.64) | 16.69  (13.23 - 20.46) | 818.14  (707.45 - 927.63) | 774.95  (638.3 - 929.46) | -1.4  (-2.37 - -0.43) |
| **Saint Kitts and Nevis** | 28.94  (25.3 - 32.62) | 20.73  (17.57 - 23.93) | 10.5  (9.12 - 11.95) | 13.84  (11.63 - 16.18) | -1.53  (-2.37 - -0.68) |
| **Saint Lucia** | 26.28  (23.19 - 29.54) | 19.84  (16.92 - 22.96) | 25.68  (22.37 - 29.18) | 40.85  (34.84 - 47.07) | -1.3  (-2.17 - -0.43) |
| **Saint Vincent and the Grenadines** | 36.32  (31.28 - 41.43) | 25.97  (22.58 - 29.55) | 28.6  (24.55 - 32.69) | 33.47  (29.03 - 38.07) | -1.52  (-2.26 - -0.77) |
| **Suriname** | 38.82  (33.94 - 44.7) | 28.07  (24.5 - 31.94) | 114.43  (99.27 - 131.05) | 168.29  (146.46 - 191.4) | -1.44  (-2.14 - -0.73) |
| **Trinidad and Tobago** | 36.46  (32.13 - 41.48) | 26.13  (22.92 - 29.79) | 334.09  (290.74 - 380.47) | 451.76  (396.14 - 515.73) | -1.31  (-2.03 - -0.58) |
| **United States Virgin Islands** | 20.96  (18.17 - 23.88) | 18.63  (15.63 - 21.84) | 19.67  (16.74 - 22.57) | 26.2  (22.71 - 30.22) | -0.55  (-1.5 - 0.4) |
| **Central Latin America** | 28.9  (25.21 - 33.49) | 17.01  (14.7 - 19.65) | 26915.84  (23235.01 - 30771.61) | 40821.38  (35234.64 - 47182.89) | -2.06  (-2.91 - -1.2) |
| **Colombia** | 26.52  (23.64 - 29.76) | 15.25  (12.93 - 17.71) | 5203.32  (4612.65 - 5894.42) | 7930.66  (6735.33 - 9208) | -2.06  (-2.96 - -1.16) |
| **Costa Rica** | 14.48  (12.74 - 16.33) | 9.45  (8.2 - 10.78) | 262.85  (229.65 - 296.34) | 484.16  (418.92 - 552.72) | -1.81  (-2.99 - -0.61) |
| **El Salvador** | 39.04  (34.65 - 44.19) | 18.29  (16.12 - 20.52) | 1239.94  (1102.09 - 1408.06) | 1116.74  (984.86 - 1251.35) | -3.15  (-3.94 - -2.34) |
| **Guatemala** | 47.82  (42.55 - 53.96) | 31.81  (28.65 - 35.32) | 2045.64  (1784.77 - 2321.48) | 3916.27  (3494.88 - 4365.97) | -1.66  (-2.25 - -1.06) |
| **Honduras** | 38.67  (33.92 - 44.88) | 29.28  (25.63 - 33.75) | 969.93  (845.21 - 1112.76) | 1951.28  (1707.12 - 2248.58) | -1.3  (-1.99 - -0.61) |
| **Mexico** | 31.37  (26.33 - 37.79) | 17.95  (15.21 - 21.47) | 14692.73  (12292.89 - 17483.66) | 20999.79  (17752.57 - 25053.23) | -2.18  (-3 - -1.34) |
| **Nicaragua** | 17.39  (15.29 - 19.62) | 11.82  (10.08 - 13.65) | 330.88  (286.78 - 379.04) | 604.67  (507.23 - 706.91) | -1.6  (-2.67 - -0.51) |
| **Panama** | 14.35  (12.53 - 16.34) | 10.01  (8.35 - 11.74) | 234.44  (204.41 - 267.69) | 420.08  (350.11 - 493.34) | -1.59  (-2.78 - -0.38) |
| **Venezuela (Bolivarian Republic of)** | 17.63  (15.46 - 19.78) | 11.66  (10.05 - 13.4) | 1936.12  (1686.43 - 2179.27) | 3397.72  (2913.95 - 3936.11) | -1.71  (-2.78 - -0.64) |
| **Tropical Latin America** | 66.97  (56.01 - 80.3) | 21.61  (18.09 - 25.98) | 72775.46  (60374.22 - 85984.4) | 52581.37  (43926.92 - 63390.01) | -4.43  (-5.05 - -3.8) |
| **Brazil** | 67.56  (56.48 - 81.07) | 21.54  (18 - 25.92) | 71655.53  (59389.45 - 84831.53) | 51103.25  (42673.68 - 61688.7) | -4.47  (-5.09 - -3.85) |
| **Paraguay** | 43.19  (37.52 - 49.38) | 23.9  (20.2 - 27.54) | 1119.92  (962.3 - 1285.71) | 1478.12  (1229.6 - 1721.08) | -2.22  (-2.96 - -1.48) |
| **North Africa and Middle East** | 44.95  (38.15 - 51.83) | 38.59  (32.06 - 44.99) | 106886.97  (88691.53 - 124469.38) | 215511.16  (175085.59 - 256242.9) | -0.51  (-1.14 - 0.14) |
| **Afghanistan** | 91.11  (77.26 - 106.27) | 80.09  (68.49 - 92.89) | 7381.16  (6239.39 - 8644.51) | 17543.77  (14554.18 - 20565.96) | -0.66  (-1.09 - -0.23) |
| **Algeria** | 47.64  (41.3 - 54.56) | 36.31  (30.62 - 42.25) | 7391.37  (6210.65 - 8561.2) | 13992.53  (11542.09 - 16564.55) | -1.29  (-1.93 - -0.64) |
| **Bahrain** | 68.83  (59.76 - 78.75) | 47.14  (39.83 - 54.82) | 165.46  (137.05 - 197.32) | 599.97  (463.57 - 759.48) | -1.62  (-2.16 - -1.08) |
| **Egypt** | 37.95  (32.25 - 43.92) | 34.11  (28.79 - 39.69) | 15562.22  (12978.8 - 18117.83) | 29277.24  (24206.87 - 34392.48) | -0.53  (-1.23 - 0.18) |
| **Iran (Islamic Republic of)** | 47.06  (39.18 - 55.76) | 35.42  (29.06 - 41.88) | 17661.11  (14295.68 - 20894.28) | 29853.78  (23916.85 - 35749.1) | -1.19  (-1.83 - -0.55) |
| **Iraq** | 32.81  (27.65 - 38.27) | 29.48  (23.72 - 35.33) | 3772.39  (3108.7 - 4531.7) | 10865.02  (8490.36 - 13251.26) | -0.45  (-1.21 - 0.33) |
| **Jordan** | 36.05  (31.11 - 41.33) | 32.01  (26.12 - 38.39) | 721.12  (596.04 - 852.81) | 3180.08  (2503.41 - 3893.52) | -0.61  (-1.33 - 0.11) |
| **Kuwait** | 32.06  (26.96 - 37.26) | 32.12  (25.41 - 39.19) | 425.91  (322.62 - 528.41) | 1578.46  (1188.68 - 2051.07) | -0.05  (-0.8 - 0.7) |
| **Lebanon** | 38.44  (32.65 - 44.56) | 37.69  (31 - 44.23) | 976.07  (818.79 - 1138.02) | 2004.03  (1645.76 - 2374.88) | -0.17  (-0.84 - 0.51) |
| **Libya** | 39.22  (33.74 - 44.82) | 33.34  (27.54 - 39.19) | 1026.2  (853.88 - 1197.79) | 2246.51  (1792.43 - 2724.05) | -0.78  (-1.47 - -0.07) |
| **Morocco** | 53.74  (46.67 - 61.75) | 39.96  (34.03 - 45.76) | 9491.92  (8066.22 - 10906.8) | 13670.43  (11500.86 - 15773.29) | -1.28  (-1.89 - -0.67) |
| **Oman** | 44.34  (37.94 - 50.86) | 36.83  (30.04 - 43.91) | 520.16  (422.02 - 615.65) | 1534.15  (1129.9 - 1965.58) | -0.91  (-1.57 - -0.25) |
| **Palestine** | 34.46  (28.51 - 40.8) | 32.81  (26.42 - 39.58) | 452.79  (363.75 - 549.78) | 1287.81  (1003.44 - 1608.54) | 0.13  (-0.6 - 0.86) |
| **Qatar** | 43.47  (36.79 - 50.41) | 39.85  (32 - 48.09) | 130.81  (96.98 - 167.69) | 1171.21  (830.83 - 1542.95) | -0.3  (-0.95 - 0.36) |
| **Saudi Arabia** | 35.76  (30.53 - 41.19) | 31.82  (25.6 - 38.02) | 3660.34  (2969.93 - 4391.03) | 11463.27  (8572.82 - 14489.45) | -0.42  (-1.15 - 0.32) |
| **Sudan** | 48.19  (41.37 - 55.34) | 41.67  (35.38 - 47.98) | 5959.93  (4970.25 - 6949.13) | 12050.69  (9817.73 - 14231.17) | -0.75  (-1.37 - -0.13) |
| **Syrian Arab Republic** | 18.79  (15.81 - 21.99) | 21.11  (16.64 - 25.85) | 1462.98  (1191.09 - 1755.33) | 3009.5  (2340.5 - 3716.72) | 0.04  (-0.89 - 0.97) |
| **Tunisia** | 41.32  (35.59 - 47.27) | 36.07  (29.77 - 42.7) | 2533.84  (2133.23 - 2938.89) | 4501.57  (3695.18 - 5372.43) | -0.66  (-1.33 - 0.02) |
| **Turkey** | 44.14  (36.49 - 52.06) | 44.58  (35.63 - 54.72) | 21928.38  (17728.39 - 26280) | 40837.2  (32312.03 - 50588.4) | 1  (0.39 - 1.61) |
| **United Arab Emirates** | 42.23  (36.37 - 49.1) | 35.86  (29.72 - 42.17) | 478.44  (366.44 - 594.09) | 3489.66  (2526.57 - 4644.52) | -0.74  (-1.4 - -0.07) |
| **Yemen** | 72.81  (62.55 - 84.86) | 56.62  (48.9 - 64.64) | 5112.48  (4313.77 - 5938.34) | 11135.35  (9240.2 - 12961.81) | -1.12  (-1.63 - -0.61) |
| **South Asia** | 135.89  (115.2 - 161.6) | 77.46  (65.31 - 91.62) | 928339.3  (778471.71 - 1097470.62) | 1193531.26  (999121.67 - 1404392.76) | -2.24  (-2.64 - -1.83) |
| **Bangladesh** | 213.79  (183.19 - 259.04) | 53.32  (45.46 - 61.64) | 123565.4  (105645.88 - 146203.66) | 78728.46  (66331.41 - 92022.52) | -6.19  (-6.59 - -5.79) |
| **Bhutan** | 141.61  (123.19 - 164.02) | 56.69  (49.53 - 64.43) | 496.88  (424.76 - 570.56) | 362.84  (312.32 - 417.36) | -3.75  (-4.19 - -3.31) |
| **India** | 127.64  (107.2 - 152.2) | 80.81  (67.86 - 96.09) | 698978.96  (580065.51 - 832147.13) | 994281.77  (829817.99 - 1180293.96) | -1.76  (-2.17 - -1.35) |
| **Nepal** | 139.31  (120.61 - 162.15) | 70.57  (62.08 - 80.25) | 15978.67  (13675.55 - 18392.8) | 16971.16  (14861.41 - 19438.83) | -2.86  (-3.29 - -2.44) |
| **Pakistan** | 131.74  (110.94 - 157.43) | 75.34  (63.84 - 89.34) | 89319.38  (75120.41 - 105272.75) | 103187.03  (85836.93 - 121124.74) | -2.16  (-2.57 - -1.75) |
| **East Asia** | 61.76  (51.26 - 75.44) | 32.79  (27.24 - 39.11) | 611101.28  (505733.7 - 742231.67) | 650289.53  (534947.98 - 789784.71) | -2.48  (-3.11 - -1.86) |
| **China** | 61.7  (51.06 - 75.59) | 32.74  (27.19 - 39.12) | 589876.94  (485691.58 - 718016.71) | 627511.19  (515362.03 - 764100.17) | -2.48  (-3.11 - -1.85) |
| **Democratic People's Republic of Korea** | 63.02  (53.53 - 74.7) | 44.57  (38.46 - 51.63) | 11312.56  (9544.99 - 13458.95) | 14107.89  (12084.23 - 16472.44) | -1.39  (-1.94 - -0.83) |
| **Taiwan (Province of China)** | 61.55  (52.7 - 70.38) | 24.9  (20.99 - 28.93) | 9911.78  (8512.18 - 11360.99) | 8670.45  (7333.12 - 10018.91) | -3.62  (-4.25 - -2.98) |
| **Oceania** | 68.55  (59.38 - 78.22) | 56.59  (49 - 65.05) | 3137.87  (2623.64 - 3605.64) | 5681.96  (4785 - 6570.79) | -0.86  (-1.37 - -0.33) |
| **American Samoa** | 52.88  (46.05 - 60.38) | 41.91  (36.21 - 48.09) | 17.13  (14.45 - 19.92) | 21.64  (18.57 - 24.85) | -1.01  (-1.62 - -0.4) |
| **Cook Islands** | 42.28  (36.35 - 48.46) | 42.15  (35.95 - 48.76) | 6.44  (5.44 - 7.48) | 8.97  (7.68 - 10.42) | -0.06  (-0.71 - 0.59) |
| **Fiji** | 59.98  (52.29 - 67.93) | 45.65  (39.57 - 52.04) | 319.08  (268.81 - 367.05) | 379.73  (322.77 - 438.1) | -1.23  (-1.8 - -0.65) |
| **Guam** | 37.39  (31.73 - 42.97) | 31.85  (26.14 - 37.85) | 40.91  (33.54 - 48.24) | 55.94  (46.33 - 65.57) | -0.79  (-1.52 - -0.06) |
| **Kiribati** | 156.83  (135.25 - 180.62) | 129.77  (112.65 - 150.67) | 88.61  (75.1 - 102.69) | 118.39  (100.62 - 137.83) | -0.85  (-1.19 - -0.52) |
| **Marshall Islands** | 90.95  (79.06 - 105.39) | 70.08  (60.85 - 80.7) | 25.03  (21.1 - 29.42) | 30.28  (25.75 - 35.02) | -1.09  (-1.54 - -0.64) |
| **Micronesia (Federated States of)** | 103.22  (89.32 - 118.81) | 68.6  (59.06 - 79.02) | 70.96  (59.72 - 82.91) | 57.77  (48.81 - 67.03) | -1.78  (-2.22 - -1.33) |
| **Nauru** | 67.67  (58.15 - 77.73) | 60.68  (52.75 - 69.33) | 4.15  (3.48 - 4.83) | 4.28  (3.56 - 4.96) | -0.51  (-1.01 - -0.01) |
| **Niue** | 50.25  (43.11 - 57.47) | 43.58  (36.98 - 49.86) | 1.09  (0.94 - 1.25) | 0.84  (0.72 - 0.96) | -0.73  (-1.33 - -0.12) |
| **Northern Mariana Islands** | 46.04  (40.09 - 52.47) | 43.55  (37.57 - 49.9) | 14.37  (11.79 - 17.2) | 20.75  (17.9 - 23.79) | -0.33  (-0.95 - 0.29) |
| **Palau** | 45.36  (39.06 - 52.02) | 43.49  (36.94 - 50.24) | 5.64  (4.75 - 6.6) | 8.65  (7.35 - 9.97) | -0.25  (-0.87 - 0.38) |
| **Papua New Guinea** | 64.37  (55.45 - 73.91) | 54.89  (47.28 - 63.47) | 1887.81  (1571.65 - 2190.05) | 3964.98  (3313.59 - 4636.39) | -0.72  (-1.25 - -0.19) |
| **Samoa** | 72.94  (62.67 - 84.91) | 63.01  (54.59 - 72.36) | 80.99  (69.2 - 94.04) | 106.34  (91.18 - 122.44) | -0.75  (-1.24 - -0.25) |
| **Solomon Islands** | 96.22  (82.62 - 111.61) | 75.82  (65.73 - 86.88) | 212.2  (178.34 - 247.3) | 349.31  (294.69 - 403.83) | -1  (-1.44 - -0.56) |
| **Tokelau** | 53.6  (46.21 - 61.35) | 43.92  (37.84 - 50.71) | 0.75  (0.64 - 0.86) | 0.58  (0.5 - 0.67) | -0.92  (-1.52 - -0.32) |
| **Tonga** | 82.16  (70.55 - 95.14) | 63.94  (55.38 - 73.32) | 55.08  (46.57 - 64.07) | 55.08  (47.68 - 63.23) | -1.14  (-1.62 - -0.66) |
| **Tuvalu** | 61.75  (53.73 - 70.95) | 51.28  (44.51 - 58.47) | 4.74  (4.09 - 5.47) | 5.6  (4.84 - 6.42) | -0.83  (-1.38 - -0.28) |
| **Vanuatu** | 129.07  (110.22 - 152.42) | 103.29  (89.42 - 120.51) | 129.25  (109.26 - 153.83) | 224.46  (192.87 - 261.32) | -0.99  (-1.36 - -0.62) |
| **Southeast Asia** | 42.09  (36.02 - 49.07) | 33.27  (28.01 - 38.68) | 137049.33  (114750.6 - 159402.97) | 218153.31  (181117.76 - 256823.02) | -1.03  (-1.71 - -0.35) |
| **Cambodia** | 118.03  (102.12 - 140.7) | 72.17  (61.76 - 84.85) | 6548.5  (5628.46 - 7667.33) | 9240.06  (7896.57 - 10924.34) | -2.07  (-2.5 - -1.65) |
| **Indonesia** | 26.28  (21.98 - 31.42) | 22.02  (18.05 - 26.16) | 32506.42  (26603.87 - 38568.63) | 53613.25  (43029.34 - 64453.47) | -0.89  (-1.75 - -0.02) |
| **Lao People's Democratic Republic** | 117.52  (101.67 - 138.53) | 72.62  (62.81 - 84.83) | 3062.27  (2623.88 - 3574.09) | 3716.65  (3213.94 - 4300.62) | -2.04  (-2.46 - -1.63) |
| **Malaysia** | 45.63  (39.48 - 52.02) | 37.15  (32.02 - 42.62) | 4774.05  (4138.22 - 5436.36) | 10489.84  (8980.55 - 12090.43) | -1.09  (-1.71 - -0.46) |
| **Maldives** | 31.39  (27.02 - 36.26) | 27.87  (22.59 - 33.43) | 36.87  (30.74 - 43.41) | 139  (104.29 - 174.6) | -0.61  (-1.4 - 0.2) |
| **Mauritius** | 44.95  (39.14 - 51.28) | 24.34  (20.85 - 28.09) | 392.53  (338.33 - 449.53) | 389.47  (334.15 - 445.6) | -2.66  (-3.38 - -1.94) |
| **Myanmar** | 87.61  (75.76 - 104.62) | 42.3  (36.6 - 49.02) | 24992.72  (21486.93 - 29572.84) | 21171.94  (18204.59 - 24564.25) | -3  (-3.51 - -2.48) |
| **Philippines** | 96.8  (81.02 - 116.25) | 72.4  (60.6 - 87.03) | 38657.92  (31767.99 - 45691.54) | 64639.42  (53503.32 - 77492.54) | -1.09  (-1.54 - -0.64) |
| **Seychelles** | 101.39  (87.22 - 121.6) | 65.86  (56.06 - 77.59) | 60.59  (51.86 - 72.32) | 73.96  (63.02 - 87.23) | -1.79  (-2.23 - -1.34) |
| **Sri Lanka** | 19.73  (16.09 - 23.51) | 21.72  (17.16 - 26.74) | 2918.97  (2310.41 - 3543.4) | 5271.49  (4151.83 - 6493.98) | 0.31  (-0.64 - 1.27) |
| **Thailand** | 22.54  (18.82 - 26.35) | 26.54  (22 - 31) | 10855.97  (8832.43 - 12990.17) | 23890.76  (19909.21 - 27900.76) | 0.73  (-0.13 - 1.6) |
| **Timor-Leste** | 99.88  (86.48 - 118.38) | 63.6  (54.78 - 73.39) | 418.29  (356.92 - 489.98) | 583.77  (500.72 - 675.38) | -1.91  (-2.36 - -1.46) |
| **Viet Nam** | 23.8  (20.19 - 27.57) | 24.31  (19.77 - 29.15) | 11642.05  (9644.44 - 13840.27) | 24647.89  (19680.51 - 30072.53) | -0.02  (-0.89 - 0.85) |
| **Central Sub-Saharan Africa** | 58.32  (50.84 - 66.15) | 56.53  (49.48 - 64.28) | 18307.48  (15491 - 20963.75) | 43585.85  (36674.51 - 50391.93) | -0.13  (-0.68 - 0.41) |
| **Angola** | 65.12  (56.28 - 74.14) | 57.72  (50.58 - 66.37) | 3684.26  (3103.77 - 4251.8) | 9436.11  (7975.44 - 10961.75) | -0.49  (-1.01 - 0.04) |
| **Central African Republic** | 83.26  (72.65 - 95.01) | 82.07  (71.42 - 93.76) | 1362.69  (1152.4 - 1569.84) | 2597.85  (2202.74 - 2990.57) | -0.05  (-0.5 - 0.4) |
| **Congo** | 52.75  (45.77 - 59.75) | 45.02  (38.99 - 51) | 781  (657.83 - 902.52) | 1592.4  (1336.4 - 1837.76) | -0.64  (-1.23 - -0.05) |
| **Democratic Republic of the Congo** | 55.28  (48.06 - 62.96) | 56.22  (48.95 - 63.79) | 11982.32  (10133.85 - 13731.31) | 29083.2  (24447.07 - 33725.89) | 0.05  (-0.5 - 0.6) |
| **Equatorial Guinea** | 72.42  (62.93 - 83.5) | 41.47  (35.83 - 47.2) | 180.92  (154.12 - 209.14) | 361.12  (291.8 - 432.81) | -2.29  (-2.86 - -1.73) |
| **Gabon** | 47.83  (41.6 - 54.24) | 39.12  (33.6 - 44.59) | 316.29  (272.27 - 361.88) | 515.17  (430.27 - 598.44) | -0.81  (-1.44 - -0.18) |
| **Eastern Sub-Saharan Africa** | 55.73  (47.18 - 64.29) | 46.4  (39.36 - 53.33) | 66718.73  (54998.71 - 78848.47) | 128415.78  (104537.43 - 151985.74) | -0.85  (-1.43 - -0.26) |
| **Burundi** | 67.28  (57.4 - 77.92) | 61.91  (53.4 - 70.98) | 2423.46  (1996.04 - 2875.12) | 4600.57  (3821.33 - 5406.66) | -0.38  (-0.91 - 0.14) |
| **Comoros** | 57.02  (48.98 - 65.43) | 45.98  (39.26 - 52.52) | 180.24  (150.95 - 209.79) | 280.53  (238.1 - 323.11) | -0.91  (-1.48 - -0.33) |
| **Djibouti** | 52.1  (44.57 - 59.5) | 40.05  (34.18 - 45.76) | 152.42  (124.53 - 181.56) | 375.54  (310.24 - 441.44) | -1.13  (-1.74 - -0.52) |
| **Eritrea** | 61.58  (52.67 - 70.8) | 56.35  (48.61 - 64.5) | 1131.4  (933.43 - 1335.63) | 2585.68  (2144.24 - 3024.82) | -0.45  (-0.99 - 0.08) |
| **Ethiopia** | 66.28  (55.06 - 78.1) | 40.35  (33.51 - 47.61) | 21715  (17686.64 - 26179.79) | 29697.55  (23767.3 - 35676.32) | -2.07  (-2.63 - -1.5) |
| **Kenya** | 52.45  (43.73 - 61.76) | 44.55  (36.95 - 52.3) | 7168.72  (5822.51 - 8557.11) | 16298.02  (13154.07 - 19516.48) | -0.85  (-1.45 - -0.25) |
| **Madagascar** | 43.55  (37.46 - 49.79) | 46.19  (39.67 - 52.6) | 3621.13  (2995.61 - 4243.85) | 8617.27  (7037.72 - 10086.92) | 0.11  (-0.52 - 0.75) |
| **Malawi** | 51.77  (44.74 - 58.62) | 55.66  (47.93 - 63.63) | 3081.45  (2593.52 - 3558.63) | 6697.43  (5590.83 - 7854.27) | 0.12  (-0.45 - 0.7) |
| **Mozambique** | 52.38  (45.19 - 60.39) | 56.24  (48.01 - 64.89) | 4466.12  (3747.09 - 5228.74) | 10571.21  (8703.34 - 12498.59) | 0.25  (-0.33 - 0.83) |
| **Rwanda** | 91.85  (79.07 - 105.84) | 67.44  (58.44 - 77.3) | 4031.61  (3360.12 - 4738.69) | 5772.78  (4894.54 - 6724.23) | -1.37  (-1.83 - -0.91) |
| **Somalia** | 57.97  (49.28 - 66.92) | 59.49  (50.12 - 68.79) | 2460.61  (2021.43 - 2917.77) | 7353.91  (6041.59 - 8772.58) | 0.03  (-0.51 - 0.56) |
| **South Sudan** | 44.86  (38.55 - 50.98) | 42.59  (36.52 - 48.62) | 1710.54  (1408.74 - 2000.15) | 2600.46  (2141.08 - 3062.15) | -0.24  (-0.87 - 0.39) |
| **Uganda** | 54.82  (46.42 - 63.63) | 51.93  (44.36 - 59.12) | 5525.53  (4541.96 - 6510.18) | 12802.71  (10507.76 - 15204.46) | -0.41  (-0.97 - 0.16) |
| **United Republic of Tanzania** | 37.31  (31.99 - 42.39) | 37.61  (32 - 43.06) | 6264.39  (5187.05 - 7369.42) | 14909.89  (12091.85 - 17748.36) | -0.11  (-0.79 - 0.59) |
| **Zambia** | 55.05  (47.47 - 62.84) | 41.19  (35.22 - 46.88) | 2737.14  (2264.02 - 3234.69) | 5149.58  (4232.12 - 6137.84) | -1.26  (-1.84 - -0.67) |
| **Southern Sub-Saharan Africa** | 38.97  (32.94 - 45.33) | 38.12  (32.21 - 44.34) | 14646.17  (12122.52 - 17114.12) | 25658.01  (21394.25 - 29845.64) | -0.09  (-0.75 - 0.58) |
| **Botswana** | 62.15  (53.73 - 71.46) | 46.23  (40.2 - 52.81) | 491.6  (415.79 - 567.4) | 837.55  (706.34 - 967.76) | -1.08  (-1.62 - -0.54) |
| **Eswatini** | 45.59  (39.76 - 51.82) | 45.08  (39.44 - 50.81) | 207.73  (173.85 - 242.38) | 366.81  (308.16 - 426.32) | -0.06  (-0.66 - 0.54) |
| **Lesotho** | 62.58  (54.67 - 71.94) | 66.46  (57.33 - 76.07) | 748.05  (640.9 - 856.1) | 1102.29  (931.04 - 1275.81) | 0.2  (-0.31 - 0.71) |
| **Namibia** | 55.2  (48.36 - 62.81) | 48.1  (41.5 - 54.57) | 497.58  (427.68 - 572.11) | 845.94  (719.32 - 973.86) | -0.61  (-1.17 - -0.04) |
| **South Africa** | 35.09  (29.33 - 41.45) | 34.51  (28.81 - 40.79) | 9779.89  (8004.73 - 11523.08) | 17457.74  (14430.51 - 20515.89) | -0.04  (-0.73 - 0.66) |
| **Zimbabwe** | 45.82  (39.74 - 52.28) | 48.04  (41.19 - 54.87) | 2921.32  (2435.39 - 3411.06) | 5047.69  (4246.19 - 5884.26) | 0.12  (-0.5 - 0.74) |
| **Western Sub-Saharan Africa** | 62.88  (54.01 - 72.41) | 58.17  (49.88 - 66.88) | 85115.68  (70574.83 - 99889.16) | 189288.34  (154409.97 - 223298.15) | -0.68  (-1.2 - -0.16) |
| **Benin** | 59.88  (52 - 68.01) | 54.13  (46.72 - 62.18) | 1905.9  (1584.2 - 2209.95) | 4505.19  (3680.31 - 5355.73) | -0.57  (-1.12 - -0.02) |
| **Burkina Faso** | 67.19  (58.01 - 76.69) | 71.38  (61.58 - 81.78) | 4367.82  (3636.43 - 5128.73) | 10872.7  (9001.81 - 12641.12) | 0.22  (-0.28 - 0.72) |
| **Cabo Verde** | 55.46  (47.5 - 63.31) | 33.13  (27.29 - 39.21) | 157.48  (131.04 - 182.88) | 181.79  (145.85 - 219.16) | -2.22  (-2.89 - -1.55) |
| **Cameroon** | 64.19  (55.85 - 72.56) | 52.72  (45.48 - 60.33) | 4464.88  (3725.95 - 5134.42) | 10759.38  (8793.35 - 12722.15) | -0.93  (-1.46 - -0.39) |
| **Chad** | 77.76  (67.97 - 88.71) | 68.95  (59.97 - 77.61) | 3195.79  (2725.03 - 3691.65) | 7311.23  (6012.87 - 8564.4) | -0.58  (-1.05 - -0.12) |
| **Cote d'Ivoire** | 64.49  (56.05 - 73.88) | 60.11  (51.81 - 68.43) | 5066.4  (4152.91 - 5987.35) | 10900.56  (8939.18 - 12702.98) | -0.5  (-1.01 - 0.02) |
| **Gambia** | 69.43  (59.93 - 79.26) | 54.55  (47.39 - 62.23) | 429.09  (356.41 - 499.47) | 863.22  (718.03 - 1013.84) | -1.03  (-1.55 - -0.52) |
| **Ghana** | 42.12  (36.13 - 48.13) | 49.35  (42.13 - 56.58) | 4544.41  (3730.05 - 5384.96) | 12811.79  (10578.74 - 14996.65) | 0.68  (0.05 - 1.31) |
| **Guinea** | 67.66  (58.52 - 76.79) | 58.05  (50.23 - 66.39) | 3083.14  (2601.8 - 3548.7) | 5141.53  (4270.94 - 6037.55) | -0.76  (-1.27 - -0.24) |
| **Guinea-Bissau** | 86.52  (74.96 - 99.59) | 67.61  (58.17 - 77.02) | 583.63  (484.82 - 682.13) | 901.92  (735.16 - 1058.81) | -1.04  (-1.49 - -0.58) |
| **Liberia** | 58.09  (50.33 - 66.34) | 50.24  (43.09 - 57.33) | 912.96  (760.8 - 1080.43) | 1793.4  (1451.08 - 2124.6) | -0.85  (-1.4 - -0.29) |
| **Mali** | 66.35  (57.3 - 75.48) | 64.4  (55.67 - 72.97) | 4156.94  (3451.23 - 4871.93) | 9388.23  (7733.68 - 10975.63) | -2.42  (-2.88 - -1.96) |
| **Mauritania** | 59.39  (51.38 - 67.32) | 44.95  (38.45 - 51.6) | 868.99  (727.39 - 999.93) | 1405.33  (1154.63 - 1666.17) | -1.24  (-1.81 - -0.67) |
| **Niger** | 81.19  (70.36 - 92.72) | 71.82  (62.19 - 82.07) | 4266.28  (3528.52 - 5040.47) | 9954.31  (8190.53 - 11720.37) | -2.85  (-3.27 - -2.43) |
| **Nigeria** | 62.28  (52.18 - 72.53) | 57.19  (47.91 - 66.7) | 40582.72  (33052.17 - 48304.21) | 89236.8  (71127.26 - 107851.93) | -0.42  (-0.95 - 0.11) |
| **Sao Tome and Principe** | 45.69  (39.19 - 52.19) | 35.69  (30.09 - 41.53) | 42.12  (34.67 - 50.01) | 60.3  (48.31 - 72.91) | -1.11  (-1.74 - -0.47) |
| **Senegal** | 65.28  (56.83 - 73.54) | 53.7  (46.16 - 61.34) | 3341.76  (2796.72 - 3852.93) | 6020.5  (5034.4 - 6995.77) | -0.9  (-1.44 - -0.37) |
| **Sierra Leone** | 60.06  (52.01 - 68.74) | 63.12  (54.81 - 71.44) | 1675.05  (1400.01 - 1956.34) | 3806.1  (3172.06 - 4484.58) | 0.22  (-0.31 - 0.75) |
| **Togo** | 62.91  (54.45 - 71.64) | 60.27  (52.39 - 68.45) | 1467.49  (1211.98 - 1729.38) | 3371.42  (2847.34 - 3899.94) | -0.29  (-0.83 - 0.24) |
| **High SDI^§^** | 44.44  (37.74 - 52.09) | 34.21  (28.81 - 39.97) | 439002.19  (371561.95 - 516852.66) | 516386.23  (436242.14 - 610537.82) | -0.81  (-1.48 - -0.14) |
| **High-middle SDI** | 51.92  (43.78 - 61.66) | 36.15  (30.21 - 42.67) | 583181.06  (486082.84 - 691573.5) | 665892.95  (557315.21 - 794748.49) | -1.35  (-1.98 - -0.71) |
| **Middle SDI** | 54.8  (45.96 - 65.47) | 33.6  (28.17 - 39.45) | 676760.48  (561208.07 - 801379.38) | 846507.45  (707929.23 - 996724.61) | -1.65  (-2.3 - -0.99) |
| **Low-middle SDI** | 111.7  (95.08 - 133.17) | 66.28  (56.11 - 78.09) | 813789.53  (686845.17 - 959581.75) | 999590.12  (841807.25 - 1172915.58) | -2.09  (-2.53 - -1.64) |
| **Low SDI** | 95.41  (81.6 - 111.26) | 66.37  (56.78 - 76.26) | 301358.23  (252750.35 - 350720.33) | 479004.6  (399694.42 - 557056.57) | -1.36  (-1.83 - -0.9) |

*Age-standardized rate (per 100,000 population)

¶ Estimated annual percentage change

§ Socio-demographic index

**Additional file 1:**

**Table S3. DALYs of PUD in 1990 and 2019 for both sexes and estimated annual percentage change in age-standardized rates by location**

| **Characteristic** | **Rate^*^** | | **Number** | | **EAPC^¶^** |
| --- | --- | --- | --- | --- | --- |
|  | **No (95% UI)** | | **No (95% UI)** | | **No (95% CI)** |
|  | **1990** | **2019** | **1990** | **2019** | **1990-2019** |
| **Global** | 189.03  (175.52 - 205.63) | 74.4  (68.96 - 81.95) | 8196063.99  (7581034.84 - 8965447.06) | 6029509.85  (5586597.71 - 6641773.45) | -3.44  (-3.81 - -3.08) |
| **Central Asia** | 124.65  (117.61 - 130.83) | 93.57  (83.57 - 104.97) | 66639.83  (63021.72 - 70056.22) | 78504.54  (69725.74 - 88632.08) | -1.58  (-1.94 - -1.23) |
| **Armenia** | 134.33  (122.41 - 146.7) | 108.64  (90.49 - 128.77) | 3990.48  (3624.66 - 4352.15) | 4360.6  (3625.46 - 5177.55) | -0.73  (-1.1 - -0.36) |
| **Azerbaijan** | 141.35  (116 - 156.66) | 66.45  (52.12 - 87.63) | 8257.22  (6676.06 - 9200.13) | 6796.71  (5218.26 - 8829.13) | -3.69  (-4.07 - -3.31) |
| **Georgia** | 127.25  (113.47 - 144.16) | 83.89  (66.6 - 103.8) | 7793.93  (6945.98 - 8823.65) | 4424.95  (3493.89 - 5461.07) | -1.07  (-1.49 - -0.64) |
| **Kazakhstan** | 91.36  (81.12 - 103.05) | 75.53  (62.9 - 91.21) | 12940.32  (11471.12 - 14582.09) | 14120.77  (11695.37 - 17128.12) | -1.23  (-1.61 - -0.84) |
| **Kyrgyzstan** | 134.45  (120.62 - 148.29) | 57.47  (47.92 - 68.62) | 4503.15  (4038.52 - 4976.75) | 3164.65  (2614.8 - 3819.48) | -3.73  (-4.12 - -3.33) |
| **Mongolia** | 338.11  (275.84 - 411.27) | 213.32  (161.84 - 289.88) | 4616.14  (3704.01 - 5634.64) | 5437.15  (4106.52 - 7312.69) | -1.7  (-1.93 - -1.46) |
| **Tajikistan** | 159.91  (139.45 - 177.7) | 91.66  (72.6 - 118.53) | 5515.93  (4837.63 - 6119.04) | 6161.47  (4860.09 - 8250.3) | -2.88  (-3.2 - -2.55) |
| **Turkmenistan** | 108.79  (98.18 - 119.65) | 95.68  (73.53 - 121.5) | 2696.51  (2439.69 - 2967.11) | 4627.86  (3543.85 - 5882.68) | -0.8  (-1.18 - -0.42) |
| **Uzbekistan** | 119.14  (107.67 - 129.79) | 107.62  (88.46 - 128.4) | 16326.16  (14727.31 - 17879.68) | 29410.38  (23896.35 - 35621.71) | -1.21  (-1.53 - -0.88) |
| **Central Europe** | 125.04  (120.37 - 129.48) | 62.06  (54.36 - 70.2) | 178161  (171916.08 - 184597.82) | 120051.69  (105467.45 - 136072.52) | -2.5  (-2.93 - -2.07) |
| **Albania** | 68.93  (61.86 - 79) | 19.73  (14.65 - 25.59) | 1533.59  (1364.25 - 1837.32) | 752.12  (548.73 - 984.43) | -4.99  (-5.7 - -4.27) |
| **Bosnia and Herzegovina** | 124.19  (113.71 - 136.84) | 40.22  (31.95 - 50.33) | 5154.22  (4709.93 - 5701.35) | 2250.29  (1779.98 - 2836.23) | -4.91  (-5.4 - -4.43) |
| **Bulgaria** | 114.4  (105.05 - 124.17) | 59.61  (45.86 - 75.66) | 13327.1  (12237.7 - 14500.52) | 7604.62  (5823.83 - 9624.79) | -2.61  (-3.06 - -2.16) |
| **Croatia** | 103.27  (93.98 - 112.54) | 45.31  (35.81 - 57.83) | 6382.63  (5794.84 - 6984.32) | 3725.46  (2927.43 - 4762.87) | -2.92  (-3.4 - -2.45) |
| **Czechia** | 120.11  (111.68 - 129.12) | 51.56  (41 - 62.98) | 15847.22  (14752.74 - 16996.03) | 9773.38  (7772.27 - 12013.53) | -2.81  (-3.26 - -2.35) |
| **Hungary** | 166.73  (156.93 - 177.47) | 79.25  (64.43 - 96.38) | 22798.52  (21498.18 - 24169.36) | 13757.06  (11167.52 - 16720.77) | -2.9  (-3.26 - -2.53) |
| **Montenegro** | 62.4  (52.16 - 76.64) | 46.14  (37.89 - 56.2) | 394.55  (328.92 - 484.78) | 418  (339.14 - 512.18) | -1.04  (-1.56 - -0.52) |
| **North Macedonia** | 94.83  (78.29 - 108.98) | 41.38  (33.14 - 51.35) | 1790.22  (1467.44 - 2055.46) | 1239.27  (981.91 - 1558.91) | -3.43  (-3.93 - -2.94) |
| **Poland** | 137.37  (130.99 - 143.22) | 75.36  (62.9 - 88.73) | 58561.2  (55854.35 - 61046.47) | 47443.27  (39851.88 - 55588.66) | -1.88  (-2.29 - -1.46) |
| **Romania** | 105.34  (97.76 - 112.72) | 43.35  (34.95 - 52.7) | 28298.57  (26200.54 - 30300.16) | 13421.93  (10812.97 - 16301.65) | -3.63  (-4.12 - -3.14) |
| **Serbia** | 126.03  (106.49 - 146.58) | 83  (66.58 - 102.41) | 13785.19  (11625.44 - 16314.33) | 12587.97  (9959.77 - 15551.23) | -1.13  (-1.51 - -0.74) |
| **Slovakia** | 131.57  (116.04 - 152.05) | 67.15  (50.91 - 88.38) | 7622.8  (6742.61 - 8769.79) | 5768.62  (4379.09 - 7520.09) | -1.85  (-2.27 - -1.43) |
| **Slovenia** | 111.86  (86.57 - 142.89) | 31.34  (23.8 - 42.59) | 2665.18  (2063.04 - 3388.52) | 1309.72  (980.81 - 1813.65) | -4.97  (-5.46 - -4.48) |
| **Eastern Europe** | 109.29  (101.16 - 116.04) | 102.49  (90.96 - 115.64) | 294332.14  (273255.39 - 312026.51) | 312621.81  (277408.91 - 352292.53) | -0.87  (-1.23 - -0.5) |
| **Belarus** | 80.79  (71.37 - 91.01) | 49.1  (37.13 - 64.41) | 10105.81  (8973 - 11333.11) | 6863.24  (5148.09 - 9131.59) | -2.11  (-2.59 - -1.63) |
| **Estonia** | 111.07  (99.61 - 123.74) | 56.25  (42.9 - 73.15) | 2160.09  (1930.26 - 2407.45) | 1218.28  (926.4 - 1568.64) | -2.99  (-3.41 - -2.58) |
| **Latvia** | 100.08  (91.06 - 110.23) | 70.36  (56.51 - 88.19) | 3405.97  (3107.34 - 3736.25) | 2340.63  (1899.59 - 2928.36) | -1.77  (-2.16 - -1.37) |
| **Lithuania** | 82.33  (74.4 - 91.43) | 107.64  (84.79 - 137.12) | 3563.53  (3223.12 - 3968.86) | 5217.15  (4115.89 - 6610.02) | 1.16  (0.77 - 1.55) |
| **Republic of Moldova** | 166.22  (151.54 - 182.82) | 101.32  (83.36 - 121.02) | 7583.09  (6914.29 - 8356.11) | 5282.41  (4347.08 - 6310.18) | -1.91  (-2.24 - -1.57) |
| **Russian Federation** | 117.09  (105.84 - 124.1) | 106.1  (91.55 - 122.43) | 206868.71  (187472.32 - 219026.91) | 226613.53  (196180 - 260527.46) | -0.99  (-1.34 - -0.65) |
| **Ukraine** | 91.83  (83.88 - 100.5) | 105.56  (85.76 - 130.06) | 60644.94  (55696.87 - 66136.52) | 65086.57  (52718.03 - 80148.44) | -0.19  (-0.6 - 0.22) |
| **Australasia** | 76.7  (70.29 - 82.05) | 14.68  (12.74 - 16.63) | 17673.92  (16229.8 - 18914.15) | 7291.16  (6271.58 - 8342.4) | -6.58  (-7.33 - -5.82) |
| **Australia** | 74.36  (67.49 - 80.57) | 14  (11.95 - 16.19) | 14234.53  (12929.32 - 15422.54) | 5853.17  (4954.82 - 6828.04) | -6.66  (-7.43 - -5.88) |
| **New Zealand** | 88.33  (81.27 - 95.36) | 18.32  (15.73 - 21.05) | 3439.4  (3173.74 - 3719.52) | 1437.99  (1222.19 - 1666.04) | -6.2  (-6.88 - -5.51) |
| **High-income Asia Pacific** | 76.54  (71.41 - 86.67) | 21.39  (18.59 - 24.1) | 146317.2  (137174.2 - 165580.58) | 87700  (75518.1 - 97866.98) | -4.68  (-5.33 - -4.01) |
| **Brunei Darussalam** | 227.74  (194.3 - 275.63) | 51.25  (43.5 - 60.24) | 246.62  (207.5 - 303.99) | 145.96  (119.73 - 175.17) | -6.06  (-6.46 - -5.65) |
| **Japan** | 63.07  (58.98 - 66.49) | 22.71  (19.88 - 25.75) | 100167.8  (94370.73 - 105406.74) | 69644.88  (59607.69 - 78669.78) | -3.66  (-4.33 - -2.99) |
| **Republic of Korea** | 149.21  (132.59 - 204.47) | 20.7  (17.17 - 24.21) | 43867.43  (39236.8 - 61846.56) | 16623.43  (13887.21 - 19387.27) | -7.8  (-8.38 - -7.22) |
| **Singapore** | 92.54  (83.77 - 101.17) | 17.4  (14.36 - 20.42) | 2035.34  (1834.34 - 2237.94) | 1285.74  (1062.68 - 1525.48) | -6.38  (-7 - -5.75) |
| **High-income North America** | 54.28  (51 - 57.26) | 20.65  (18.7 - 22.84) | 188126.71  (176552.08 - 198541.12) | 119405.73  (108526.34 - 131585.37) | -3.99  (-4.73 - -3.25) |
| **Canada** | 56.74  (52.45 - 61.09) | 19.12  (16.42 - 22.04) | 18059.83  (16717.04 - 19456.08) | 12287.04  (10544.5 - 14175.55) | -4.44  (-5.18 - -3.69) |
| **Greenland** | 495.2  (413.56 - 595.47) | 189.82  (148.55 - 242.69) | 197.65  (161.54 - 240.88) | 130.58  (100.88 - 167.01) | -3.89  (-4.12 - -3.65) |
| **United States of America** | 54.03  (50.7 - 57.12) | 20.8  (18.77 - 23.03) | 169864.93  (158882.48 - 179657.47) | 106986.22  (96924.03 - 117762.21) | -3.95  (-4.68 - -3.21) |
| **Southern Latin America** | 69.64  (64.69 - 74.64) | 31.18  (27.76 - 35.25) | 32070.36  (29826.38 - 34418.07) | 25362.59  (22556.41 - 28729.39) | -2.87  (-3.49 - -2.25) |
| **Argentina** | 66.21  (60.09 - 72.59) | 35.36  (30.44 - 41.26) | 21172.71  (19220.55 - 23267.53) | 18663.15  (16043.92 - 21828.44) | -2.16  (-2.77 - -1.54) |
| **Chile** | 81.26  (74.72 - 88.94) | 22.73  (19.78 - 26.19) | 8394.75  (7701.03 - 9188.95) | 5304.45  (4629.2 - 6150.69) | -4.6  (-5.23 - -3.97) |
| **Uruguay** | 66.36  (59.83 - 73.52) | 26.99  (22.57 - 31.84) | 2501.6  (2251.4 - 2777.31) | 1393.71  (1160.92 - 1640.98) | -3.49  (-4.12 - -2.87) |
| **Western Europe** | 72.98  (68.8 - 75.67) | 22.44  (20.72 - 24.33) | 408583.84  (383915.96 - 423901.96) | 197466.03  (179438.79 - 215617.45) | -4.65  (-5.28 - -4.01) |
| **Andorra** | 24.9  (18.19 - 34.74) | 11.93  (8.97 - 15.25) | 12.94  (9.39 - 18.26) | 16.67  (12.49 - 21.38) | -2.73  (-3.73 - -1.72) |
| **Austria** | 80.58  (74.66 - 86.95) | 18.1  (15.59 - 20.67) | 9117.68  (8470.63 - 9778.66) | 3020.26  (2610.85 - 3443.38) | -5.78  (-6.45 - -5.1) |
| **Belgium** | 65.31  (60.19 - 70.32) | 21.43  (18.52 - 24.65) | 9678.81  (8904.3 - 10415.24) | 4832.33  (4064.39 - 5610.12) | -4.3  (-4.95 - -3.65) |
| **Cyprus** | 73.59  (52.04 - 101.2) | 19.79  (16.34 - 25.81) | 488.57  (357.78 - 642.9) | 348.85  (288.03 - 450.07) | -5.23  (-5.87 - -4.59) |
| **Denmark** | 98.03  (90.97 - 105.66) | 47.94  (41.09 - 55.6) | 7834.14  (7262.99 - 8435.14) | 5546.65  (4715.34 - 6488.42) | -3.19  (-3.63 - -2.76) |
| **Finland** | 95.54  (87.3 - 104.11) | 29.88  (25.65 - 35.24) | 6539.24  (5986.53 - 7109.4) | 3246.84  (2762.77 - 3813.17) | -4.58  (-5.11 - -4.04) |
| **France** | 67.14  (62.27 - 72.39) | 14.23  (12.33 - 16.27) | 54722.45  (50522.2 - 59004.42) | 18853.12  (16052.08 - 21805.06) | -6.31  (-7.07 - -5.54) |
| **Germany** | 72.78  (67.27 - 77.53) | 28.98  (25.56 - 33.26) | 88109.51  (80901.16 - 93969.72) | 52756.58  (45889.56 - 61144.2) | -3.54  (-4.14 - -2.93) |
| **Greece** | 37.79  (34.87 - 40.63) | 36.64  (31.55 - 42.03) | 5466.99  (5043.53 - 5892.1) | 8845.47  (7562.64 - 10191.38) | 0.13  (-0.55 - 0.81) |
| **Iceland** | 53.84  (47.38 - 60.12) | 13.52  (11.4 - 15.88) | 150.55  (132.22 - 167.84) | 71.41  (59.86 - 84.07) | -5.48  (-6.26 - -4.7) |
| **Ireland** | 105.42  (97.5 - 113.77) | 21.59  (18.19 - 25.49) | 4263.14  (3953.05 - 4614.99) | 1589.4  (1335.58 - 1870.96) | -5.97  (-6.52 - -5.42) |
| **Israel** | 42.33  (37.64 - 47.02) | 9.88  (8.43 - 11.84) | 1995.66  (1772.69 - 2214.72) | 1147.76  (970.35 - 1380.76) | -6.45  (-7.31 - -5.58) |
| **Italy** | 57.15  (53.61 - 59.53) | 11.06  (9.8 - 12.15) | 49103.68  (45995.6 - 51112.59) | 15549.69  (13749.36 - 17017.3) | -6.65  (-7.46 - -5.83) |
| **Luxembourg** | 62.2  (55.22 - 69.63) | 16.77  (13.96 - 19.82) | 325.01  (288.17 - 365.3) | 166.41  (137.44 - 197.03) | -5.03  (-5.73 - -4.32) |
| **Malta** | 71.37  (64.34 - 79.46) | 19.48  (16.28 - 23.12) | 298.42  (268.82 - 332.86) | 169.12  (140.45 - 200.98) | -4.87  (-5.52 - -4.21) |
| **Monaco** | 33.14  (25.58 - 41.19) | 14.03  (10.8 - 17.31) | 22.46  (17.19 - 28.05) | 12.72  (9.65 - 15.86) | -3.21  (-4.11 - -2.3) |
| **Netherlands** | 69.44  (63.54 - 75.25) | 15.15  (13.03 - 17.49) | 13812.85  (12636.69 - 15002.29) | 5077.16  (4339.8 - 5932.17) | -6  (-6.69 - -5.29) |
| **Norway** | 69.34  (64.93 - 75.64) | 30.39  (26.96 - 35.47) | 4662.07  (4347.35 - 5100.19) | 2906.36  (2555.39 - 3409.49) | -2.81  (-3.38 - -2.23) |
| **Portugal** | 83.01  (75.38 - 90.92) | 19.11  (16.4 - 22.19) | 10771.76  (9755.59 - 11814.53) | 4447.82  (3798.53 - 5130.86) | -6.02  (-6.66 - -5.37) |
| **San Marino** | 25.42  (20.93 - 30.33) | 12.61  (8.83 - 17.46) | 8.19  (6.7 - 9.8) | 8.43  (5.86 - 11.71) | -2.57  (-3.56 - -1.56) |
| **Spain** | 71.49  (66.15 - 76.46) | 12.9  (11.18 - 14.83) | 37081.98  (34296.39 - 39675.34) | 12016.41  (10203.1 - 13858.53) | -6.91  (-7.68 - -6.14) |
| **Sweden** | 70.51  (65.21 - 75.12) | 27.71  (24.46 - 31.04) | 10215.56  (9376.85 - 10926.83) | 5597.51  (4893.14 - 6324.53) | -3.46  (-4.07 - -2.85) |
| **Switzerland** | 36.4  (32.25 - 41.42) | 15.44  (12.61 - 18.82) | 3736.75  (3329.28 - 4229.55) | 2783.86  (2239.11 - 3473.53) | -3.29  (-4.09 - -2.48) |
| **United Kingdom** | 101.57  (89.93 - 106.29) | 40.31  (37.36 - 43.58) | 89826.18  (78829.06 - 94091.76) | 48283.06  (44311.22 - 52181.41) | -3.69  (-4.19 - -3.19) |
| **Andean Latin America** | 192.94  (165.98 - 222.43) | 59.1  (47.42 - 73.36) | 50156.24  (42562.88 - 58745.49) | 33763.13  (27057.54 - 41817.42) | -4.24  (-4.64 - -3.85) |
| **Bolivia (Plurinational State of)** | 334.24  (236.14 - 443.25) | 119.39  (86.62 - 158.29) | 13399.55  (9004.01 - 18203.49) | 10513.45  (7528.59 - 14043.69) | -3.72  (-4.01 - -3.43) |
| **Ecuador** | 207.82  (191.37 - 224.3) | 59.82  (46.46 - 78.04) | 12899.32  (11889.61 - 13962.09) | 8900.63  (6891.22 - 11612.84) | -4.35  (-4.74 - -3.96) |
| **Peru** | 149.13  (123 - 177.13) | 43.65  (31.6 - 59.3) | 23857.37  (19586.63 - 28794.82) | 14349.05  (10430.73 - 19502.19) | -4.49  (-4.93 - -4.04) |
| **Caribbean** | 176.19  (149.55 - 209.02) | 87.4  (71.26 - 106.63) | 50889.65  (42048.86 - 61736.27) | 44111.44  (36238.39 - 53542.6) | -2.66  (-3.03 - -2.29) |
| **Antigua and Barbuda** | 75.11  (62.86 - 88.26) | 33.99  (27.75 - 40.86) | 39.99  (33.81 - 46.85) | 33.84  (27.6 - 40.62) | -3.33  (-3.9 - -2.75) |
| **Bahamas** | 125.24  (105.58 - 147.48) | 65.88  (50.71 - 84.79) | 224.19  (188.35 - 263.06) | 268.24  (204.61 - 345.47) | -2.63  (-3.06 - -2.19) |
| **Barbados** | 105.8  (92.28 - 120.43) | 57.2  (45.87 - 71.38) | 292.59  (255.53 - 331.18) | 260.85  (207.41 - 324.77) | -2.71  (-3.17 - -2.25) |
| **Belize** | 74.68  (64.44 - 86.35) | 50.38  (40.85 - 60.84) | 82.3  (71.73 - 95.08) | 157.93  (129.03 - 191.07) | -1.93  (-2.43 - -1.43) |
| **Bermuda** | 120.76  (104.47 - 138.32) | 31.53  (24.78 - 39.97) | 75.54  (65.2 - 86.81) | 37.29  (29.18 - 48.01) | -5.22  (-5.75 - -4.68) |
| **Cuba** | 89.5  (81.41 - 99.07) | 43.23  (34.06 - 55.16) | 9283.42  (8447.45 - 10302.23) | 7909.02  (6210.2 - 10058.84) | -3.32  (-3.82 - -2.81) |
| **Dominica** | 107.67  (91.41 - 124.45) | 59.4  (45.12 - 76.59) | 73.57  (62.51 - 84.86) | 50.93  (38.62 - 65.48) | -2.49  (-2.95 - -2.02) |
| **Dominican Republic** | 153.35  (134.15 - 178.7) | 84.12  (55.85 - 113.34) | 7762.38  (6801.34 - 9130.64) | 8321.49  (5463.57 - 11327.62) | -1.89  (-2.29 - -1.5) |
| **Grenada** | 160.15  (135.29 - 186.77) | 76.21  (63.61 - 90.67) | 111.49  (95.26 - 129.52) | 85.56  (70.91 - 103.31) | -2.79  (-3.19 - -2.39) |
| **Guyana** | 339.43  (289.54 - 393.26) | 168.26  (126.37 - 220.76) | 1577.68  (1344.3 - 1828.51) | 1143.41  (847.07 - 1513.01) | -2.22  (-2.49 - -1.95) |
| **Haiti** | 518.7  (366.15 - 678.11) | 226.71  (154.76 - 315.36) | 22346.49  (14047.8 - 31913.26) | 18664.96  (12398.13 - 26070.28) | -2.82  (-3.04 - -2.6) |
| **Jamaica** | 125  (111.36 - 138.29) | 74.96  (57.34 - 95.35) | 2233.25  (1996.53 - 2464.47) | 2251.77  (1726.87 - 2865.97) | -2.19  (-2.6 - -1.78) |
| **Puerto Rico** | 72.22  (64.45 - 80.7) | 14.51  (11.06 - 18.83) | 2583.91  (2307.88 - 2893.69) | 897.09  (680.34 - 1166.67) | -6.84  (-7.6 - -6.09) |
| **Saint Kitts and Nevis** | 124.86  (103.78 - 146.64) | 41.39  (30.97 - 53.33) | 44.28  (36.9 - 51.96) | 27.61  (20.17 - 36.15) | -4.51  (-5.01 - -4.01) |
| **Saint Lucia** | 121.14  (105.45 - 137.12) | 51.7  (41.91 - 63.82) | 111.25  (97.23 - 126.17) | 109.3  (87.89 - 135.34) | -3.65  (-4.12 - -3.18) |
| **Saint Vincent and the Grenadines** | 186.61  (158.59 - 215.52) | 94.34  (75.65 - 115.37) | 141.33  (120.08 - 163.13) | 125.24  (99.81 - 153.49) | -2.67  (-3.03 - -2.31) |
| **Suriname** | 204.23  (174.98 - 230.04) | 107.23  (85.93 - 131.43) | 587.82  (502.07 - 662.24) | 646.03  (515.09 - 793.45) | -2.71  (-3.04 - -2.39) |
| **Trinidad and Tobago** | 177.37  (159.29 - 199.77) | 87.49  (62.4 - 118.97) | 1558.44  (1394.74 - 1768.15) | 1561.29  (1105.29 - 2136.19) | -2.92  (-3.27 - -2.57) |
| **United States Virgin Islands** | 71.46  (58.75 - 86.64) | 38.96  (30.08 - 48.98) | 64.45  (52.94 - 78.69) | 65.33  (50.3 - 82.04) | -2.27  (-2.85 - -1.68) |
| **Central Latin America** | 193.4  (183.71 - 199.77) | 67.99  (58.6 - 79.33) | 186333.54  (178571.09 - 193006.14) | 161366.17  (139617.45 - 188761.69) | -4.13  (-4.52 - -3.75) |
| **Colombia** | 172.05  (160.66 - 182.65) | 36.45  (27.87 - 47.89) | 33802.17  (31337.93 - 36005.17) | 19222.03  (14676.51 - 25249.92) | -6  (-6.47 - -5.54) |
| **Costa Rica** | 79.42  (69.53 - 91.39) | 40.2  (29.91 - 53.45) | 1483.48  (1309.51 - 1689.41) | 2064.47  (1529.87 - 2744.25) | -3.44  (-3.97 - -2.91) |
| **El Salvador** | 325.18  (299.44 - 351.59) | 85.07  (64.08 - 110.6) | 11035.16  (10184.98 - 12011.03) | 5165.57  (3900.63 - 6717.19) | -5.73  (-6.06 - -5.41) |
| **Guatemala** | 483.55  (423.42 - 544.44) | 204.32  (160 - 258.92) | 23111.82  (20388.52 - 26241.36) | 25602.55  (19913.99 - 32799.98) | -3.44  (-3.65 - -3.23) |
| **Honduras** | 362.43  (300.14 - 425.6) | 229.8  (166.69 - 301.77) | 11124.49  (9122.57 - 13249.57) | 14431.02  (10424.76 - 19274.02) | -1.53  (-1.77 - -1.29) |
| **Mexico** | 189.73  (179.89 - 196.07) | 66.07  (56.15 - 76.29) | 89530.57  (85636.44 - 92621.87) | 76504.84  (64761.42 - 88422.21) | -4.19  (-4.59 - -3.79) |
| **Nicaragua** | 139.59  (121.88 - 155.75) | 57.75  (46.52 - 74.74) | 2680.94  (2385.55 - 3009.02) | 2576.3  (2048.2 - 3406.25) | -3.44  (-3.85 - -3.03) |
| **Panama** | 81.33  (71.68 - 91.2) | 24.37  (18.27 - 32.66) | 1331.1  (1176.76 - 1482.39) | 1017.8  (764.93 - 1364.56) | -4.29  (-4.9 - -3.68) |
| **Venezuela (Bolivarian Republic of)** | 109.98  (99.95 - 119.27) | 51.22  (38.23 - 69.42) | 12233.8  (11163.07 - 13281.7) | 14781.59  (10973.18 - 20142.83) | -3.39  (-3.86 - -2.91) |
| **Tropical Latin America** | 145.88  (137.96 - 153.14) | 50.54  (46.8 - 54.61) | 151075.41  (143296.17 - 158696.39) | 122912.49  (114129.29 - 132707.65) | -3.91  (-4.35 - -3.46) |
| **Brazil** | 147.17  (138.87 - 154.64) | 50.64  (46.87 - 54.72) | 148696.95  (141039.34 - 156152.67) | 120215.65  (111401.69 - 129758.04) | -3.93  (-4.38 - -3.49) |
| **Paraguay** | 93.55  (82.48 - 106.13) | 46.82  (35.63 - 62.3) | 2378.46  (2096.03 - 2694.92) | 2696.84  (2032.8 - 3594.53) | -2.51  (-3 - -2.01) |
| **North Africa and Middle East** | 135  (115.35 - 166.24) | 56.38  (46.8 - 67.78) | 285342.29  (245962.74 - 340929.61) | 266066.86  (217521.32 - 323932.93) | -2.95  (-3.39 - -2.52) |
| **Afghanistan** | 459.79  (304.25 - 636.81) | 255.08  (173.23 - 351.83) | 37204.92  (23248.53 - 53423.88) | 45557.67  (30764.24 - 63725.06) | -2.02  (-2.23 - -1.81) |
| **Algeria** | 157.05  (121.19 - 202.21) | 53.26  (40.99 - 68.01) | 20493.01  (15676.22 - 26087.92) | 17026.53  (12817.97 - 21668.88) | -3.8  (-4.23 - -3.37) |
| **Bahrain** | 232.85  (197.26 - 275.58) | 76.88  (62.41 - 93.54) | 332.44  (283.61 - 392.99) | 441.74  (354.57 - 545.51) | -4.71  (-5.02 - -4.39) |
| **Egypt** | 135.67  (120.16 - 154.83) | 62.27  (35.42 - 94.15) | 50928.93  (43853.85 - 58083.51) | 46679.13  (26115.21 - 71626.98) | -2.26  (-2.69 - -1.83) |
| **Iran (Islamic Republic of)** | 136.54  (116.2 - 162.62) | 42.48  (36.91 - 47.09) | 43917.13  (38526.78 - 52215.76) | 31249.32  (27033.37 - 34680.19) | -3.9  (-4.37 - -3.43) |
| **Iraq** | 76.13  (58.31 - 101.67) | 25.12  (19.29 - 31.17) | 7341.67  (5548.79 - 9858.8) | 7008.61  (5386.08 - 8834.44) | -3.9  (-4.5 - -3.29) |
| **Jordan** | 114.07  (87.69 - 142.32) | 31.07  (25.22 - 37.28) | 1581.18  (1252.49 - 1987.29) | 1996.61  (1610.85 - 2421) | -4.86  (-5.36 - -4.35) |
| **Kuwait** | 56.17  (48.17 - 66.16) | 21.84  (17.33 - 27.74) | 401.69  (347.49 - 464.71) | 629.1  (491.12 - 799.96) | -2.83  (-3.54 - -2.11) |
| **Lebanon** | 103.55  (71.18 - 140.76) | 42.35  (25.19 - 66.39) | 2329.69  (1610.59 - 3162.54) | 2199.9  (1303.1 - 3443.45) | -2.99  (-3.49 - -2.48) |
| **Libya** | 123.49  (81.66 - 176.35) | 53.49  (35.49 - 76.15) | 2739.58  (1935.15 - 3796.26) | 2886.72  (1909.17 - 4167.08) | -3.07  (-3.54 - -2.6) |
| **Morocco** | 176.61  (130.4 - 237.29) | 76.22  (57.95 - 94.52) | 27030.13  (20766.25 - 34656.77) | 22923.26  (17421.45 - 29133.57) | -2.94  (-3.31 - -2.56) |
| **Oman** | 119.94  (85.24 - 168.05) | 39.72  (32.46 - 48.96) | 909.28  (653.52 - 1243.9) | 724.79  (561.29 - 897.28) | -3.32  (-3.82 - -2.82) |
| **Palestine** | 63.24  (41.6 - 91.23) | 30.16  (25.18 - 35.69) | 626.4  (407.4 - 949.03) | 770.75  (642.09 - 915.01) | -2.76  (-3.39 - -2.12) |
| **Qatar** | 60.62  (47.43 - 79.06) | 28.54  (22.48 - 36.12) | 77.08  (58.84 - 99.89) | 277.28  (203.07 - 363.21) | -2.06  (-2.66 - -1.45) |
| **Saudi Arabia** | 103.29  (71.87 - 156.85) | 34.19  (26.57 - 44.8) | 6646.52  (4726.48 - 9686.51) | 6897.84  (5179.59 - 8878.14) | -3.6  (-4.13 - -3.08) |
| **Sudan** | 188.17  (109.19 - 306.69) | 91.51  (52.72 - 143.97) | 25010.93  (14454.64 - 39658.69) | 21112.15  (12942.26 - 33370.89) | -2.32  (-2.67 - -1.97) |
| **Syrian Arab Republic** | 55.94  (43.18 - 70.27) | 20.77  (15.42 - 27.32) | 4367.84  (3349.98 - 5647.97) | 2515.18  (1808.43 - 3406.08) | -3.98  (-4.69 - -3.26) |
| **Tunisia** | 100.09  (70.73 - 133.98) | 42.36  (30.51 - 58.64) | 5377.61  (3811.89 - 7345.89) | 5059.16  (3635.48 - 7025.15) | -3.04  (-3.54 - -2.54) |
| **Turkey** | 63.74  (50.97 - 78.13) | 25.87  (21.12 - 31.33) | 27573.96  (21647.96 - 34765.56) | 22443.11  (18187.97 - 27254.13) | -2.87  (-3.52 - -2.22) |
| **United Arab Emirates** | 128.82  (92.71 - 188.33) | 46.69  (32.83 - 64.96) | 592.78  (421.6 - 885.61) | 2024.03  (1378.71 - 2861.4) | -3.33  (-3.75 - -2.91) |
| **Yemen** | 305.26  (191.35 - 510.57) | 159.2  (109.32 - 235.82) | 19667.59  (12724.13 - 30814.21) | 25373.65  (17862.02 - 36262.54) | -2.62  (-2.89 - -2.35) |
| **South Asia** | 460.21  (398.02 - 519.11) | 139.95  (119.9 - 165.02) | 3186950.01  (2781023.76 - 3620032.83) | 2098565.71  (1792973.51 - 2479691.08) | -4.47  (-4.72 - -4.23) |
| **Bangladesh** | 472.23  (360.78 - 590.21) | 36.49  (25.73 - 47.42) | 263228.06  (199148.47 - 329289.06) | 52980.69  (36626.01 - 69150.57) | -10.18  (-10.53 - -9.83) |
| **Bhutan** | 536.76  (337.82 - 862.8) | 150.32  (77.12 - 280.11) | 1725.08  (1034.46 - 2752.48) | 891.21  (453.34 - 1691.09) | -4.79  (-5.03 - -4.55) |
| **India** | 508.13  (439.82 - 583.84) | 158.99  (134.9 - 189.45) | 2808756.01  (2445055.7 - 3226227.07) | 1919785.57  (1628168.81 - 2296551.63) | -4.36  (-4.59 - -4.13) |
| **Nepal** | 76.21  (56.87 - 100.88) | 27.97  (21.27 - 39.19) | 7323.94  (5598.4 - 9420.11) | 5698.96  (4362.75 - 7794.92) | -3.89  (-4.51 - -3.27) |
| **Pakistan** | 171.37  (124.53 - 255.24) | 98.1  (74.53 - 132.19) | 105916.91  (78942.96 - 152014.49) | 119209.28  (90946.82 - 160663.81) | -2.09  (-2.43 - -1.75) |
| **East Asia** | 175.64  (147.15 - 215.67) | 46.8  (40.41 - 54.42) | 1644626.03  (1367815.39 - 2031345.01) | 918055.23  (788013.68 - 1073455.91) | -4.36  (-4.77 - -3.95) |
| **China** | 174.3  (144.87 - 215.92) | 45.34  (38.74 - 53.74) | 1578589.08  (1302694.77 - 1969516.78) | 857470.92  (729915.01 - 1016719.16) | -4.42  (-4.83 - -4) |
| **Democratic People's Republic of Korea** | 223.15  (161.1 - 304.05) | 144.72  (87.86 - 202.76) | 37935.94  (26379 - 52393.36) | 45887.2  (27247.23 - 64791.13) | -1.4  (-1.69 - -1.11) |
| **Taiwan (Province of China)** | 190.87  (176.67 - 205.09) | 38.31  (29.52 - 49.08) | 28101  (26148.61 - 30199.86) | 14697.1  (11269.46 - 18942.13) | -6.27  (-6.7 - -5.85) |
| **Oceania** | 294.91  (227.21 - 362.57) | 185.47  (149.66 - 231.48) | 11993.12  (9410.99 - 14874.13) | 17040.62  (13522.83 - 21769.86) | -1.61  (-1.87 - -1.34) |
| **American Samoa** | 192.62  (160.82 - 230.81) | 99.41  (80.21 - 119.73) | 51.61  (42.98 - 61.49) | 48.06  (38.64 - 58.22) | -2.66  (-3.02 - -2.31) |
| **Cook Islands** | 77.73  (61.15 - 98.92) | 27.37  (22.24 - 33.95) | 10.29  (7.98 - 13.11) | 6.19  (5.03 - 7.7) | -3.97  (-4.57 - -3.36) |
| **Fiji** | 236.33  (182.65 - 297.69) | 132.89  (101.69 - 172.69) | 1067.33  (813.93 - 1345.84) | 1022.1  (772.14 - 1349.3) | -2.12  (-2.43 - -1.81) |
| **Guam** | 86  (73.07 - 101.33) | 33.82  (26.68 - 41.75) | 68.43  (57.67 - 81.81) | 62.02  (48.82 - 76.75) | -3.94  (-4.53 - -3.35) |
| **Kiribati** | 909.52  (643.04 - 1187.74) | 512.71  (362.22 - 701.02) | 417.23  (286.55 - 572.01) | 409.72  (269.91 - 584.57) | -2.11  (-2.26 - -1.96) |
| **Marshall Islands** | 494.24  (356.22 - 653.09) | 246.77  (162.32 - 345.24) | 109.91  (79.45 - 145.15) | 98.26  (60.66 - 141.52) | -2.46  (-2.67 - -2.25) |
| **Micronesia (Federated States of)** | 544.63  (403.65 - 726.93) | 237.53  (148.65 - 337.8) | 315.97  (229.27 - 431.68) | 182.92  (109.19 - 265.79) | -3.19  (-3.41 - -2.97) |
| **Nauru** | 403.03  (273.17 - 598.96) | 229.91  (161.9 - 314.87) | 21.28  (14.01 - 33.64) | 13.37  (8.03 - 19.32) | -1.81  (-2.02 - -1.59) |
| **Niue** | 231.16  (171.99 - 311.38) | 109.12  (80.34 - 141.81) | 5.09  (3.84 - 6.85) | 2.22  (1.64 - 2.88) | -2.91  (-3.23 - -2.59) |
| **Northern Mariana Islands** | 150.39  (120.96 - 187.2) | 72.98  (59.45 - 87.05) | 33.3  (25.62 - 43.16) | 35.25  (28.14 - 43.13) | -2.5  (-2.9 - -2.11) |
| **Palau** | 164.6  (109.34 - 237.72) | 101.99  (64 - 133.54) | 17.78  (11.64 - 25.9) | 19.94  (12 - 26.58) | -1.58  (-1.94 - -1.21) |
| **Papua New Guinea** | 274.94  (201.24 - 352.18) | 185.91  (142.86 - 243.91) | 7408.85  (5613.67 - 9544.29) | 12265.75  (9231.48 - 16336.84) | -1.3  (-1.57 - -1.03) |
| **Samoa** | 327.28  (215.06 - 474.47) | 173.41  (130.22 - 225.62) | 320.54  (209.49 - 462.62) | 265.65  (197.84 - 350.92) | -2.2  (-2.47 - -1.94) |
| **Solomon Islands** | 453.21  (289.24 - 644) | 254.69  (194.32 - 332.14) | 856.35  (539.87 - 1226.05) | 1055.42  (791.11 - 1405.79) | -1.91  (-2.13 - -1.69) |
| **Tokelau** | 283.57  (186.19 - 399.79) | 116.3  (87.01 - 148.81) | 3.93  (2.58 - 5.58) | 1.48  (1.11 - 1.92) | -3.2  (-3.49 - -2.9) |
| **Tonga** | 339.44  (269.43 - 413.08) | 182.04  (136.79 - 233.31) | 200.95  (157.39 - 243.72) | 148.56  (112.14 - 189.61) | -2.26  (-2.51 - -2) |
| **Tuvalu** | 434.18  (281.74 - 623.58) | 185.03  (128.29 - 255.98) | 32.28  (20.66 - 47.36) | 19.13  (13.11 - 26.57) | -2.94  (-3.18 - -2.69) |
| **Vanuatu** | 478.25  (289.62 - 725.72) | 293.42  (209.17 - 406.35) | 388.32  (238.17 - 573.98) | 579.72  (413.98 - 808.4) | -2.12  (-2.32 - -1.92) |
| **Southeast Asia** | 203.9  (181.47 - 226.74) | 78.62  (69.74 - 91.38) | 617601.18  (545291.92 - 707941.37) | 480443.88  (424498.86 - 562739.39) | -3.53  (-3.88 - -3.17) |
| **Cambodia** | 1048.88  (829.26 - 1261.36) | 427.74  (330.42 - 562.93) | 59992.64  (45572.6 - 74940.59) | 50504.8  (38166.14 - 67974.68) | -3.28  (-3.43 - -3.13) |
| **Indonesia** | 60.55  (51.86 - 70.47) | 23.92  (19.6 - 27.45) | 70981.89  (60196.91 - 87638.97) | 44366.48  (37326.47 - 50982.81) | -3.3  (-3.94 - -2.66) |
| **Lao People's Democratic Republic** | 1075.82  (828.4 - 1367.07) | 397.46  (282.9 - 545.23) | 26330.71  (19991.69 - 33315.97) | 18545.64  (12996.93 - 25604.64) | -3.65  (-3.81 - -3.5) |
| **Malaysia** | 151.35  (130.99 - 177.68) | 104.8  (78.93 - 131.77) | 14058.48  (12334.06 - 16299) | 26096.7  (19749.1 - 33059.86) | -2.28  (-2.61 - -1.95) |
| **Maldives** | 128.43  (74.71 - 184.19) | 26.33  (21.55 - 31.76) | 126.53  (68.65 - 188.53) | 83.78  (68.24 - 99.75) | -6.47  (-7.01 - -5.92) |
| **Mauritius** | 189.17  (171.69 - 208.48) | 49.55  (38.91 - 61.97) | 1572.68  (1421.7 - 1744.93) | 819.05  (635.1 - 1027.31) | -4.77  (-5.19 - -4.34) |
| **Myanmar** | 665.52  (499.13 - 863.16) | 165.5  (129.51 - 226.29) | 192526.65  (138493.64 - 261488.73) | 81668.01  (62464.35 - 113674.24) | -4.83  (-5.04 - -4.62) |
| **Philippines** | 603.89  (525.92 - 719.68) | 245.46  (203.15 - 295.16) | 206647.15  (182802.79 - 238468.85) | 204392.48  (168163.73 - 247466.74) | -3.37  (-3.58 - -3.17) |
| **Seychelles** | 355.45  (305.01 - 416.18) | 155.87  (126.2 - 186.97) | 203.37  (175.49 - 236.29) | 171.21  (137.77 - 206.85) | -3.21  (-3.47 - -2.94) |
| **Sri Lanka** | 37.12  (31.02 - 43.24) | 11.17  (8.5 - 14.98) | 4622.61  (3707.81 - 5406.62) | 2652.51  (1996.98 - 3570.33) | -3.64  (-4.58 - -2.7) |
| **Thailand** | 48.54  (39.93 - 58.07) | 34.11  (25.72 - 46.45) | 18863.64  (15254.12 - 22603.02) | 32984.71  (24609.18 - 44925.64) | -0.94  (-1.61 - -0.26) |
| **Timor-Leste** | 696.51  (493.31 - 1046.61) | 348.16  (228.91 - 521.47) | 2684.72  (1860.92 - 3974.42) | 2877.1  (1871.48 - 4327.06) | -2.82  (-3.01 - -2.64) |
| **Viet Nam** | 46.49  (35.64 - 59.37) | 17.11  (13.05 - 22.23) | 18169.17  (13981.1 - 22888.09) | 14651.97  (11080.9 - 18974.99) | -3.89  (-4.66 - -3.1) |
| **Central Sub-Saharan Africa** | 232.86  (173.08 - 295.55) | 145.85  (105.7 - 193.24) | 81149.25  (62056.79 - 101722.81) | 111625.08  (82299.62 - 149231.82) | -1.41  (-1.71 - -1.12) |
| **Angola** | 282.93  (177.19 - 389.44) | 147.89  (98.47 - 208.04) | 17770.62  (11993.58 - 24061.86) | 23653.97  (16894.09 - 32835.02) | -2.16  (-2.44 - -1.88) |
| **Central African Republic** | 449.22  (299.29 - 668.87) | 377.18  (228.76 - 573.09) | 7772.38  (5353.39 - 11300.43) | 12107.85  (7201.4 - 18538.65) | -0.59  (-0.79 - -0.39) |
| **Congo** | 246.99  (180.26 - 323.7) | 121.71  (82.76 - 173.44) | 3622.31  (2599.76 - 4961.52) | 4002.71  (2555.62 - 5867.47) | -2.33  (-2.63 - -2.03) |
| **Democratic Republic of the Congo** | 202.89  (146.61 - 263.61) | 135.74  (92.5 - 188.39) | 49872.25  (35804 - 68922.28) | 70253.23  (48156.3 - 97932.16) | -1.11  (-1.42 - -0.81) |
| **Equatorial Guinea** | 337.64  (206.41 - 515.23) | 78.41  (45.49 - 122.23) | 914.56  (541.48 - 1360.18) | 547.64  (310.82 - 873.99) | -5.88  (-6.21 - -5.56) |
| **Gabon** | 179.36  (133.52 - 276.74) | 86.42  (58.24 - 124.35) | 1197.13  (879.97 - 1777.94) | 1059.69  (708.2 - 1587.06) | -2.28  (-2.63 - -1.93) |
| **Eastern Sub-Saharan Africa** | 213.81  (159.32 - 297.21) | 114.53  (78.28 - 153.03) | 242610.05  (176934.71 - 334377.58) | 282484.72  (206782.05 - 367084.63) | -2.26  (-2.58 - -1.94) |
| **Burundi** | 303.14  (199.98 - 505.46) | 209.35  (118.82 - 354.13) | 10403.49  (6741.51 - 17553.47) | 14618.07  (8866.75 - 24313.69) | -1.56  (-1.82 - -1.3) |
| **Comoros** | 208.93  (84.32 - 347.19) | 119.54  (63.78 - 181.32) | 591.27  (220.28 - 992.53) | 679.81  (371.47 - 1009.14) | -2.22  (-2.54 - -1.91) |
| **Djibouti** | 179.21  (85.33 - 311.6) | 108.87  (48.18 - 187.15) | 452.94  (227.58 - 796.17) | 918.68  (426.06 - 1574.57) | -1.81  (-2.13 - -1.48) |
| **Eritrea** | 252.74  (159.02 - 361.25) | 186.56  (133.57 - 258.88) | 4318.7  (2659.26 - 6176.81) | 7802.52  (5281.06 - 10952.68) | -0.95  (-1.22 - -0.68) |
| **Ethiopia** | 321.07  (206.4 - 507.8) | 89.3  (49.96 - 131.13) | 99559.22  (60001.49 - 154216.26) | 56151.63  (34082.79 - 81668.62) | -4.66  (-4.96 - -4.36) |
| **Kenya** | 177.37  (112.43 - 308.46) | 128.65  (75.73 - 188.91) | 21100.46  (14174.16 - 35717.4) | 40629.16  (24861.09 - 59434.54) | -0.82  (-1.14 - -0.5) |
| **Madagascar** | 151.67  (92.48 - 216.8) | 114.8  (80.67 - 153.01) | 12517.11  (8075.23 - 17401.71) | 19490.82  (13867.5 - 25713.61) | -0.75  (-1.11 - -0.38) |
| **Malawi** | 168.96  (117.84 - 224.69) | 114.71  (71.3 - 165.25) | 10235.3  (7239.55 - 13869) | 12856.76  (8336.58 - 18211.74) | -1.5  (-1.83 - -1.17) |
| **Mozambique** | 143.69  (95.99 - 195.02) | 144.33  (97.72 - 208.87) | 12059.71  (8273.43 - 16296.32) | 24508.24  (16727.95 - 34712.9) | 0.74  (0.39 - 1.09) |
| **Rwanda** | 402.91  (296.58 - 573.33) | 161.08  (105.23 - 256.27) | 16850.04  (12220.07 - 24547.84) | 13078.28  (8748.06 - 20869.52) | -4.22  (-4.46 - -3.98) |
| **Somalia** | 248.06  (114.99 - 410.83) | 208.97  (106.88 - 354.41) | 9925.43  (4747.05 - 16419.27) | 23048.35  (12170.11 - 39036.57) | -0.62  (-0.89 - -0.36) |
| **South Sudan** | 155.91  (75.82 - 288.04) | 114.26  (61.82 - 211) | 5467.62  (2789.81 - 10198.83) | 6256.25  (3633.02 - 11948.28) | -1.06  (-1.41 - -0.71) |
| **Uganda** | 163.27  (107.01 - 240.54) | 125.63  (89.1 - 201.97) | 15230.85  (10155.88 - 22255.07) | 27787.47  (19418.12 - 42148.47) | -1.4  (-1.72 - -1.08) |
| **United Republic of Tanzania** | 90.09  (66.01 - 123.12) | 64.99  (37.55 - 97.62) | 14871.39  (11379.47 - 20081.5) | 23539.03  (14713.42 - 34343.48) | -0.9  (-1.36 - -0.44) |
| **Zambia** | 188.44  (143.81 - 248.36) | 97.22  (73.75 - 135.26) | 8848.49  (6761.97 - 11682.33) | 10893.81  (8052.91 - 14757.07) | -2.51  (-2.83 - -2.19) |
| **Southern Sub-Saharan Africa** | 138.18  (100.54 - 176.94) | 112.16  (98.32 - 128.5) | 46734.22  (34666.82 - 58808.99) | 69784.37  (59573.87 - 81797.97) | -0.27  (-0.61 - 0.07) |
| **Botswana** | 260.67  (169.2 - 395.02) | 151.98  (105.9 - 220.64) | 1732.58  (1090.84 - 2657.24) | 2444.83  (1636.54 - 3740.7) | -2.12  (-2.38 - -1.86) |
| **Eswatini** | 175.52  (108.57 - 274.77) | 162.89  (108.63 - 233.76) | 634.88  (395.1 - 995.33) | 1103.5  (705.06 - 1644.58) | 0.34  (0.05 - 0.63) |
| **Lesotho** | 257.21  (150.08 - 392.38) | 315.04  (224.71 - 427.66) | 2772.38  (1581.49 - 4269.66) | 4645.74  (3220.58 - 6401.73) | 1.45  (1.21 - 1.68) |
| **Namibia** | 224.38  (162.78 - 287.24) | 136.52  (99.07 - 190.35) | 1747.08  (1242.68 - 2235.66) | 2079.74  (1471.21 - 2952.62) | -1.63  (-1.92 - -1.33) |
| **South Africa** | 116.96  (83.95 - 148.95) | 83.84  (74.18 - 93.63) | 30382.2  (22237.48 - 37982.48) | 39400.37  (34578.32 - 44130.18) | -0.57  (-0.94 - -0.19) |
| **Zimbabwe** | 181.4  (127.1 - 246.88) | 219.55  (121.51 - 309.66) | 9465.1  (6605.24 - 12968.98) | 20110.19  (10987.49 - 28989.1) | 1.07  (0.78 - 1.35) |
| **Western Sub-Saharan Africa** | 242.82  (185.24 - 331.41) | 166.09  (122.34 - 220.96) | 308698  (234714.4 - 434061.78) | 474886.59  (341306.12 - 667446.39) | -1.1  (-1.38 - -0.81) |
| **Benin** | 231.37  (161.32 - 329.53) | 173.31  (128.16 - 230.9) | 6945.42  (4821.91 - 9502.85) | 12708.93  (8941.98 - 17488.42) | -0.82  (-1.11 - -0.53) |
| **Burkina Faso** | 136.67  (107.62 - 169.15) | 127.84  (80.38 - 198.82) | 7994.29  (6111.23 - 10253.43) | 17295.31  (11216.02 - 26519.33) | 0.09  (-0.28 - 0.45) |
| **Cabo Verde** | 227.33  (170.5 - 284.47) | 56.13  (45.9 - 67.76) | 588.23  (454.55 - 731.92) | 266  (218.71 - 322.72) | -5.48  (-5.89 - -5.06) |
| **Cameroon** | 230.43  (183.99 - 280.41) | 144.77  (92.03 - 203.6) | 13990.21  (11256.36 - 17267.39) | 25052.86  (15036.19 - 36162.16) | -1.53  (-1.81 - -1.25) |
| **Chad** | 302.38  (204.81 - 405.32) | 254.67  (191.04 - 332.12) | 11712.92  (8235.37 - 15585.61) | 23199.73  (17221.71 - 30217.45) | -0.48  (-0.71 - -0.24) |
| **Cote d'Ivoire** | 221.58  (155.44 - 300.56) | 161.4  (118.67 - 211.83) | 14921.88  (10466.28 - 20518.7) | 25858.76  (18002.33 - 34566.97) | -1.2  (-1.47 - -0.92) |
| **Gambia** | 279.2  (192.35 - 382.37) | 165.29  (118.95 - 220.24) | 1494.4  (1005.27 - 2102.37) | 2130.97  (1537.63 - 2829.91) | -1.97  (-2.24 - -1.7) |
| **Ghana** | 91.89  (64.28 - 132.26) | 58.7  (44.1 - 76.23) | 8419.04  (6057.97 - 11616.62) | 12993.27  (9572.43 - 16854.11) | -1.15  (-1.61 - -0.69) |
| **Guinea** | 248.43  (170.23 - 353.78) | 193.97  (130.1 - 263.74) | 11276.77  (7579.85 - 15662.87) | 15630.01  (10082.49 - 21851.15) | -0.49  (-0.77 - -0.22) |
| **Guinea-Bissau** | 444.81  (326.08 - 584.45) | 292.77  (213.77 - 391.48) | 2699.03  (1891.59 - 3591.6) | 3193.98  (2294.66 - 4349.03) | -1.08  (-1.29 - -0.87) |
| **Liberia** | 243.56  (169.37 - 342.75) | 152.8  (100.32 - 217.63) | 3650.15  (2508.95 - 5449.43) | 4484.12  (2727.38 - 6746.81) | -1.65  (-1.95 - -1.36) |
| **Mali** | 274.67  (171.37 - 401.71) | 211.39  (125.03 - 321.76) | 16801.06  (10353.1 - 24059.82) | 27918.42  (16424.8 - 43260.95) | -0.69  (-0.96 - -0.42) |
| **Mauritania** | 258.13  (180.12 - 324.27) | 102.66  (69.96 - 137.14) | 3293.73  (2361.3 - 4143.23) | 2622.46  (1783.84 - 3635.72) | -2.98  (-3.3 - -2.67) |
| **Niger** | 317.67  (203.88 - 453.02) | 257.77  (150.3 - 384.56) | 16437.61  (10119.41 - 24437.66) | 33079.69  (19652.29 - 50863.51) | -0.67  (-0.92 - -0.43) |
| **Nigeria** | 266  (168.5 - 434.68) | 171.11  (97.14 - 301.72) | 166096.89  (104326.63 - 288823.33) | 233156.35  (127368.13 - 429944.36) | -1.24  (-1.52 - -0.97) |
| **Sao Tome and Principe** | 135.41  (96.89 - 182.11) | 68.41  (48.06 - 111.11) | 112.65  (78.37 - 156.22) | 89.43  (62.51 - 141.98) | -2.36  (-2.74 - -1.99) |
| **Senegal** | 251.28  (156.81 - 352.67) | 160.48  (96.4 - 234.31) | 11711.55  (7536.09 - 16455.96) | 15538.79  (9278.67 - 22968.58) | -1.42  (-1.71 - -1.13) |
| **Sierra Leone** | 222.86  (146.83 - 320.94) | 199.65  (131.59 - 282.76) | 5973.13  (3984.39 - 8565.3) | 10637.05  (6987.99 - 15333.18) | 0.25  (-0.04 - 0.53) |
| **Togo** | 231.86  (167.96 - 305.72) | 185.76  (128.38 - 249.64) | 4568.67  (3343.48 - 6106.68) | 9023.79  (6079.66 - 12310.03) | -0.37  (-0.66 - -0.09) |
| **High SDI^§^** | 72.83  (69.05 - 75.79) | 24.19  (22.08 - 26.18) | 742858.56  (704263.95 - 772001.78) | 431321.1  (391452.25 - 465696.68) | -4.27  (-4.9 - -3.63) |
| **High-middle SDI** | 110.95  (103.07 - 119.79) | 45.09  (41.8 - 48.56) | 1204522.81  (1118917.82 - 1303365.98) | 876014.35  (810891.51 - 942390.29) | -3.36  (-3.82 - -2.89) |
| **Middle SDI** | 167.55  (151.89 - 186.28) | 58.49  (53.83 - 65.11) | 1936100.9  (1750648.42 - 2177436.41) | 1426040.7  (1313180.14 - 1583778.12) | -3.69  (-4.09 - -3.29) |
| **Low-middle SDI** | 441.55  (395.53 - 486.86) | 152.4  (136.73 - 175.15) | 3215761.81  (2881445.85 - 3578487.77) | 2216873.97  (1988317.05 - 2550605.82) | -3.92  (-4.16 - -3.67) |
| **Low SDI** | 342.26  (296.9 - 389.11) | 155.08  (137.15 - 175.21) | 1092914.92  (922628.83 - 1263157.47) | 1075653.08  (948157.26 - 1219387.17) | -2.89  (-3.15 - -2.63) |

*Age-standardized rate (per 100,000 population)

¶ Estimated annual percentage change

§ Socio-demographic index

**Additional file 1:**

**Table S4. PUD death in 1990 and 2019 for both sexes and estimated annual percentage change in age-standardized rates by location**

| **Characteristic** | **Rate^*^** | | **Number** | | **EAPC^¶^** |
| --- | --- | --- | --- | --- | --- |
|  | **No (95% UI)** | | **No (95% UI)** | | **No (95% CI)** |
|  | **1990** | **2019** | **1990** | **2019** | **1990-2019** |
| **Global** | 7.39  (6.87 - 7.93) | 3  (2.74 - 3.32) | 278978.51  (259455.39 - 301112.1) | 236139.49  (216761.51 - 261412.92) | -3.33  (-5.15 - -1.48) |
| **Central Asia** | 4.05  (3.79 - 4.28) | 3.44  (3.07 - 3.9) | 1959.62  (1839.35 - 2062.78) | 2445.42  (2181.62 - 2768.18) | -0.91  (-2.83 - 1.04) |
| **Armenia** | 4.83  (4.38 - 5.27) | 5.3  (4.5 - 6.3) | 129.68  (117.98 - 141.32) | 212.95  (179.96 - 253.27) | 0.82  (-0.99 - 2.67) |
| **Azerbaijan** | 4.53  (4 - 5.03) | 2.62  (1.91 - 3.79) | 240.25  (205.69 - 266.78) | 216.26  (165.26 - 293.94) | -2.7  (-4.68 - -0.69) |
| **Georgia** | 4.42  (3.93 - 4.96) | 3.24  (2.51 - 4.01) | 263.21  (233.95 - 295.09) | 191.95  (149.16 - 235.73) | -0.46  (-2.68 - 1.82) |
| **Kazakhstan** | 2.93  (2.58 - 3.34) | 2.62  (2.19 - 3.14) | 379.45  (337.13 - 432.77) | 449.14  (371.34 - 542.12) | -0.75  (-2.89 - 1.43) |
| **Kyrgyzstan** | 4.24  (3.82 - 4.68) | 1.9  (1.59 - 2.26) | 132.69  (119.67 - 146.51) | 91.19  (75.63 - 109.28) | -3.44  (-5.63 - -1.2) |
| **Mongolia** | 13.67  (11.19 - 16.48) | 9.88  (7.36 - 15.11) | 140.01  (113.66 - 169.12) | 182.09  (135.99 - 250.16) | -1.07  (-2.19 - 0.06) |
| **Tajikistan** | 4.85  (4.22 - 5.42) | 3.31  (2.56 - 4.32) | 149.71  (130.87 - 166.55) | 168.07  (130.52 - 219.14) | -1.97  (-3.74 - -0.17) |
| **Turkmenistan** | 3.41  (3.07 - 3.75) | 2.76  (2.12 - 3.53) | 71.44  (64.46 - 78.53) | 120.73  (91.82 - 155.38) | -1.03  (-3.21 - 1.19) |
| **Uzbekistan** | 3.73  (3.35 - 4.06) | 3.77  (3.11 - 4.46) | 453.19  (407.18 - 495.42) | 813.04  (657.41 - 986.08) | -0.59  (-2.37 - 1.23) |
| **Central Europe** | 5.23  (4.97 - 5.42) | 2.78  (2.43 - 3.14) | 7173.51  (6863.55 - 7420.11) | 5922.74  (5181.13 - 6695.11) | -2.23  (-4.28 - -0.15) |
| **Albania** | 3.31  (2.91 - 3.91) | 0.91  (0.66 - 1.22) | 61.01  (54.06 - 70.05) | 37.13  (26.62 - 49.81) | -4.99  (-8.17 - -1.71) |
| **Bosnia and Herzegovina** | 5.54  (5.03 - 6.14) | 2.02  (1.59 - 2.57) | 198.92  (180.48 - 221.64) | 115.76  (90.7 - 147.56) | -4.34  (-6.55 - -2.09) |
| **Bulgaria** | 4.8  (4.31 - 5.21) | 2.58  (2.01 - 3.24) | 532  (482.8 - 576.93) | 369.22  (285.68 - 463.81) | -2.39  (-4.56 - -0.18) |
| **Croatia** | 4.7  (4.27 - 5.15) | 2.33  (1.8 - 3) | 275.01  (250.32 - 300.99) | 214.7  (164.75 - 277.93) | -2.32  (-4.46 - -0.14) |
| **Czechia** | 5.14  (4.76 - 5.55) | 2.38  (1.88 - 2.95) | 683.76  (634.91 - 738.14) | 500.2  (394.65 - 621.17) | -2.57  (-4.67 - -0.41) |
| **Hungary** | 6.92  (6.51 - 7.31) | 3.46  (2.81 - 4.22) | 948.79  (894.54 - 1005.14) | 669.56  (546.1 - 812.58) | -2.69  (-4.45 - -0.91) |
| **Montenegro** | 2.4  (1.96 - 3) | 1.93  (1.55 - 2.39) | 14.31  (11.62 - 17.82) | 17.8  (14.26 - 22.16) | -0.67  (-3.23 - 1.96) |
| **North Macedonia** | 4.26  (3.47 - 4.94) | 2.08  (1.66 - 2.57) | 72.12  (58.62 - 83.16) | 56.69  (44.47 - 71.37) | -2.88  (-5.12 - -0.59) |
| **Poland** | 5.86  (5.53 - 6.11) | 3.31  (2.78 - 3.87) | 2421.54  (2299.21 - 2517.73) | 2329.44  (1958.81 - 2720.42) | -1.89  (-3.85 - 0.1) |
| **Romania** | 3.51  (3.2 - 3.75) | 1.56  (1.25 - 1.9) | 927.4  (857.06 - 991.1) | 553.91  (445.78 - 674.3) | -3.28  (-5.86 - -0.62) |
| **Serbia** | 5.96  (4.93 - 6.95) | 4.65  (3.68 - 5.73) | 601.04  (497.13 - 708.64) | 699.21  (551.94 - 868.46) | -0.39  (-2.09 - 1.34) |
| **Slovakia** | 5.38  (4.81 - 6.05) | 3.06  (2.37 - 3.9) | 308.28  (274.95 - 349.88) | 272.94  (211.46 - 347.42) | -1.45  (-3.45 - 0.6) |
| **Slovenia** | 5.54  (4.32 - 7.07) | 1.72  (1.24 - 2.51) | 129.32  (100.83 - 164.17) | 86.19  (61.46 - 128.72) | -4.55  (-6.66 - -2.39) |
| **Eastern Europe** | 3.61  (3.41 - 3.78) | 3.6  (3.19 - 4.02) | 9662.21  (9130.82 - 10102.25) | 11873.82  (10503.17 - 13274.04) | -0.4  (-2.41 - 1.64) |
| **Belarus** | 2.93  (2.62 - 3.26) | 1.59  (1.19 - 2.13) | 367.1  (327 - 409.28) | 244.59  (182.62 - 328.95) | -2.37  (-4.95 - 0.27) |
| **Estonia** | 3.88  (3.48 - 4.32) | 2.14  (1.62 - 2.79) | 77.28  (69.42 - 86.5) | 55.68  (42.6 - 71.43) | -2.63  (-4.78 - -0.43) |
| **Latvia** | 3.54  (3.23 - 3.85) | 2.78  (2.23 - 3.52) | 124.12  (113.75 - 135.32) | 111.71  (89.47 - 138.58) | -1.27  (-3.31 - 0.82) |
| **Lithuania** | 2.98  (2.69 - 3.31) | 4.29  (3.37 - 5.46) | 131.36  (118.86 - 146.49) | 249.89  (197.74 - 318.17) | 1.64  (-0.36 - 3.69) |
| **Republic of Moldova** | 5.16  (4.7 - 5.65) | 3.1  (2.56 - 3.69) | 224.87  (204.69 - 247.01) | 170.63  (141.67 - 203.28) | -1.88  (-3.79 - 0.07) |
| **Russian Federation** | 3.85  (3.55 - 4.02) | 3.94  (3.43 - 4.5) | 6711.14  (6188.59 - 7012.33) | 8965.08  (7803.28 - 10195.76) | -0.32  (-2.21 - 1.61) |
| **Ukraine** | 3.03  (2.82 - 3.29) | 3.06  (2.48 - 3.77) | 2026.35  (1882.06 - 2191.36) | 2076.24  (1697.03 - 2553.57) | -0.47  (-2.8 - 1.92) |
| **Australasia** | 4.8  (4.26 - 5.19) | 0.89  (0.74 - 1.04) | 1060.34  (949.65 - 1143.8) | 505.03  (413.6 - 593.77) | -6.75  (-9.74 - -3.67) |
| **Australia** | 4.67  (4.1 - 5.11) | 0.85  (0.69 - 1.02) | 854.25  (755.23 - 933.27) | 406.03  (327.36 - 491.21) | -6.85  (-9.9 - -3.7) |
| **New Zealand** | 5.43  (4.88 - 5.88) | 1.13  (0.93 - 1.35) | 206.09  (186.07 - 223.49) | 99  (81.22 - 118.97) | -6.28  (-8.99 - -3.49) |
| **High-income Asia Pacific** | 4.19  (3.8 - 4.69) | 0.97  (0.8 - 1.1) | 7223.66  (6665.82 - 8055.71) | 5642.26  (4462.99 - 6651.78) | -5.45  (-8.35 - -2.45) |
| **Brunei Darussalam** | 11.78  (9.83 - 14.28) | 2.92  (2.47 - 3.44) | 8.45  (7.12 - 10.35) | 5.15  (4.24 - 6.14) | -5.58  (-7.27 - -3.87) |
| **Japan** | 3.62  (3.26 - 3.85) | 0.97  (0.8 - 1.11) | 5354.58  (4894.21 - 5637.95) | 4602.29  (3612.29 - 5544.78) | -4.81  (-7.8 - -1.71) |
| **Republic of Korea** | 8.07  (6.95 - 11.4) | 1.24  (1 - 1.49) | 1775.08  (1569.07 - 2424.93) | 977.48  (800.27 - 1163.01) | -7.34  (-9.61 - -5.02) |
| **Singapore** | 4.69  (4.25 - 5.16) | 0.81  (0.64 - 0.98) | 85.54  (77.51 - 93.94) | 57.33  (46.08 - 68.76) | -6.61  (-9.34 - -3.79) |
| **High-income North America** | 2.66  (2.44 - 2.79) | 0.85  (0.76 - 0.91) | 9721.08  (8901.81 - 10202.85) | 5698.21  (5040.03 - 6154.11) | -4.8  (-8.18 - -1.28) |
| **Canada** | 3.09  (2.75 - 3.43) | 0.93  (0.76 - 1.12) | 967.8  (868.18 - 1067.5) | 702.51  (563.44 - 857.29) | -4.99  (-8.21 - -1.66) |
| **Greenland** | 20.89  (17.67 - 25.99) | 8.88  (7.04 - 11.59) | 6.53  (5.41 - 7.9) | 5.33  (4.15 - 6.92) | -3.59  (-4.69 - -2.49) |
| **United States of America** | 2.63  (2.39 - 2.76) | 0.84  (0.76 - 0.9) | 8746.52  (7961.63 - 9204.75) | 4990.29  (4420.64 - 5382.61) | -4.79  (-8.2 - -1.26) |
| **Southern Latin America** | 3.26  (2.99 - 3.53) | 1.55  (1.35 - 1.77) | 1411.83  (1295.87 - 1525.45) | 1312.46  (1141.36 - 1498.85) | -2.7  (-5.48 - 0.15) |
| **Argentina** | 3.09  (2.76 - 3.41) | 1.74  (1.48 - 2.03) | 937.6  (843.42 - 1037.13) | 957.48  (806.37 - 1112.96) | -2  (-4.77 - 0.85) |
| **Chile** | 3.82  (3.5 - 4.2) | 1.15  (0.98 - 1.33) | 347.57  (319.12 - 380.58) | 272.7  (231.74 - 317.55) | -4.4  (-7.21 - -1.5) |
| **Uruguay** | 3.31  (2.94 - 3.66) | 1.36  (1.11 - 1.63) | 126.6  (112.22 - 140.67) | 82.22  (67.06 - 98.8) | -3.44  (-6.19 - -0.61) |
| **Western Europe** | 4.08  (3.73 - 4.28) | 1.24  (1.1 - 1.38) | 23817.94  (21802.25 - 24947.4) | 13344.63  (11614.79 - 14955.26) | -4.75  (-7.41 - -2.01) |
| **Andorra** | 1.38  (0.98 - 1.97) | 0.64  (0.46 - 0.85) | 0.58  (0.41 - 0.85) | 0.99  (0.72 - 1.31) | -2.84  (-7.08 - 1.58) |
| **Austria** | 4.2  (3.85 - 4.55) | 0.88  (0.75 - 1.03) | 507.17  (466.2 - 548.91) | 179.92  (151.05 - 210.77) | -5.99  (-8.97 - -2.92) |
| **Belgium** | 3.93  (3.51 - 4.27) | 1.24  (1.01 - 1.49) | 602.1  (543.05 - 654.99) | 347.16  (279.08 - 426.19) | -4.55  (-7.19 - -1.83) |
| **Cyprus** | 5.36  (3.52 - 8.07) | 1.46  (1.14 - 2.13) | 28.14  (19.52 - 39.35) | 23.37  (18.36 - 32.8) | -5.23  (-7.54 - -2.86) |
| **Denmark** | 5.61  (5.11 - 6.08) | 3.16  (2.64 - 3.75) | 478.5  (435.4 - 519.24) | 407.95  (336.41 - 484.7) | -2.78  (-4.51 - -1.01) |
| **Finland** | 5.02  (4.55 - 5.46) | 1.43  (1.21 - 1.71) | 350.75  (319.8 - 379.54) | 188.71  (156.52 - 225.69) | -5.1  (-7.47 - -2.67) |
| **France** | 3.93  (3.52 - 4.32) | 0.78  (0.65 - 0.92) | 3433.23  (3057.95 - 3780.13) | 1365.27  (1108.58 - 1651.03) | -6.67  (-9.86 - -3.36) |
| **Germany** | 3.68  (3.29 - 3.95) | 1.61  (1.38 - 1.88) | 4746.61  (4222.12 - 5095.57) | 3540.95  (2990.42 - 4177.15) | -3.21  (-5.78 - -0.58) |
| **Greece** | 2.21  (1.97 - 2.43) | 2.21  (1.86 - 2.59) | 314.22  (281.54 - 341.9) | 664.5  (551.1 - 800.03) | 0.12  (-2.62 - 2.94) |
| **Iceland** | 2.72  (2.33 - 3.08) | 0.69  (0.56 - 0.82) | 8.02  (6.83 - 9.09) | 4.26  (3.44 - 5.11) | -5.43  (-8.77 - -1.95) |
| **Ireland** | 6.26  (5.67 - 6.8) | 1.29  (1.06 - 1.54) | 245.84  (224.62 - 267.85) | 100.13  (82.23 - 120.29) | -6  (-8.23 - -3.71) |
| **Israel** | 2.63  (2.3 - 2.94) | 0.6  (0.48 - 0.74) | 115.1  (101.35 - 127.76) | 76.18  (60.95 - 94.95) | -6.6  (-9.99 - -3.09) |
| **Italy** | 3.23  (2.93 - 3.39) | 0.58  (0.5 - 0.64) | 2819.9  (2585.46 - 2949.41) | 1044.93  (886.64 - 1158.87) | -7  (-10.38 - -3.5) |
| **Luxembourg** | 3.44  (3.01 - 3.87) | 0.94  (0.75 - 1.12) | 17.94  (15.61 - 20.29) | 10.56  (8.34 - 12.76) | -4.97  (-7.9 - -1.95) |
| **Malta** | 3.93  (3.49 - 4.4) | 1.09  (0.88 - 1.3) | 15.64  (13.94 - 17.49) | 10.78  (8.71 - 13.01) | -4.86  (-7.57 - -2.07) |
| **Monaco** | 1.84  (1.4 - 2.31) | 0.79  (0.58 - 1) | 1.48  (1.11 - 1.86) | 0.92  (0.67 - 1.17) | -3.16  (-6.88 - 0.71) |
| **Netherlands** | 4.56  (4 - 5.05) | 0.96  (0.79 - 1.17) | 918.25  (813.01 - 1017.05) | 364.68  (298.34 - 443.85) | -6.14  (-8.85 - -3.35) |
| **Norway** | 3.94  (3.58 - 4.41) | 1.79  (1.52 - 2.18) | 290.31  (263.22 - 326.22) | 202.36  (168.67 - 249.19) | -2.87  (-5.21 - -0.48) |
| **Portugal** | 4.69  (4.17 - 5.15) | 1.09  (0.92 - 1.26) | 585.95  (523.17 - 639.5) | 310.8  (259.89 - 362.43) | -6.07  (-8.71 - -3.35) |
| **San Marino** | 1.61  (1.27 - 1.98) | 0.78  (0.53 - 1.1) | 0.51  (0.41 - 0.63) | 0.64  (0.44 - 0.89) | -2.62  (-6.53 - 1.44) |
| **Spain** | 4.05  (3.65 - 4.41) | 0.69  (0.57 - 0.81) | 2119.54  (1917 - 2300.36) | 811.58  (659.77 - 988.68) | -7.24  (-10.46 - -3.91) |
| **Sweden** | 3.84  (3.45 - 4.14) | 1.54  (1.32 - 1.76) | 622.65  (558.06 - 674.66) | 377.9  (317.19 - 433.82) | -3.47  (-6.02 - -0.84) |
| **Switzerland** | 2.04  (1.8 - 2.31) | 0.97  (0.74 - 1.27) | 226.13  (198.45 - 256.85) | 210.99  (158.5 - 282.52) | -2.9  (-6.12 - 0.43) |
| **United Kingdom** | 5.78  (4.94 - 6.13) | 2.23  (1.98 - 2.45) | 5349.6  (4554.46 - 5664.26) | 3087.47  (2720 - 3408.72) | -3.84  (-5.91 - -1.72) |
| **Andean Latin America** | 8.17  (7.03 - 9.27) | 3  (2.39 - 3.71) | 1659.39  (1427.21 - 1890.95) | 1620.06  (1296.91 - 2004.03) | -3.54  (-5.34 - -1.7) |
| **Bolivia (Plurinational State of)** | 14.75  (10.83 - 19.05) | 6.49  (4.76 - 8.44) | 440.83  (312.8 - 581.12) | 485.93  (348.24 - 645.15) | -2.96  (-4.27 - -1.63) |
| **Ecuador** | 10.49  (9.42 - 11.36) | 3.5  (2.73 - 4.6) | 505  (460.74 - 546.64) | 452.32  (349.38 - 594.72) | -3.69  (-5.37 - -1.99) |
| **Peru** | 5.67  (4.64 - 6.62) | 2.09  (1.52 - 2.84) | 713.57  (582.33 - 842.86) | 681.82  (498.25 - 925.47) | -3.61  (-5.72 - -1.45) |
| **Caribbean** | 6.7  (5.98 - 7.5) | 3.42  (2.88 - 4.07) | 1731.61  (1536.73 - 1966.76) | 1758.58  (1475.78 - 2092.47) | -2.58  (-4.44 - -0.67) |
| **Antigua and Barbuda** | 3.31  (2.76 - 3.91) | 1.65  (1.33 - 2.01) | 1.84  (1.54 - 2.15) | 1.5  (1.22 - 1.84) | -2.97  (-5.6 - -0.27) |
| **Bahamas** | 5.03  (4.22 - 5.92) | 2.66  (2.04 - 3.39) | 7.64  (6.46 - 8.98) | 9.83  (7.45 - 12.63) | -2.61  (-4.76 - -0.41) |
| **Barbados** | 4.71  (4.07 - 5.33) | 2.74  (2.18 - 3.4) | 13.97  (12.04 - 15.85) | 13.25  (10.48 - 16.45) | -2.39  (-4.51 - -0.21) |
| **Belize** | 3.35  (2.86 - 3.9) | 2.12  (1.71 - 2.58) | 3.16  (2.71 - 3.65) | 5.65  (4.56 - 6.85) | -2.05  (-4.42 - 0.38) |
| **Bermuda** | 5.8  (5.02 - 6.53) | 1.53  (1.18 - 1.98) | 3.38  (2.92 - 3.83) | 2.07  (1.57 - 2.69) | -5.14  (-7.53 - -2.7) |
| **Cuba** | 4.14  (3.74 - 4.62) | 2.16  (1.7 - 2.74) | 412.84  (375.26 - 461.74) | 426.75  (337.47 - 536.65) | -3.03  (-5.31 - -0.71) |
| **Dominica** | 4.71  (4.03 - 5.37) | 2.63  (2.03 - 3.32) | 3.39  (2.88 - 3.89) | 2.36  (1.82 - 2.99) | -2.35  (-4.54 - -0.11) |
| **Dominican Republic** | 5.92  (5.06 - 7.34) | 3.48  (2.55 - 4.67) | 223.98  (194.49 - 264.93) | 313.41  (224.46 - 423.85) | -1.45  (-3.4 - 0.55) |
| **Grenada** | 6.53  (5.6 - 7.54) | 3.37  (2.82 - 4) | 4.91  (4.21 - 5.68) | 3.49  (2.89 - 4.19) | -2.57  (-4.47 - -0.62) |
| **Guyana** | 13.99  (12.02 - 16.1) | 6.79  (5.16 - 8.68) | 51.81  (44.17 - 59.61) | 39.7  (29.47 - 51.81) | -2.37  (-3.71 - -1.01) |
| **Haiti** | 19.38  (14.13 - 24.87) | 9.65  (6.53 - 13.35) | 633.14  (449.6 - 817.68) | 616.7  (418.97 - 858.74) | -2.36  (-3.47 - -1.24) |
| **Jamaica** | 5.13  (4.51 - 5.76) | 3.17  (2.45 - 4.04) | 91.9  (80.92 - 102.94) | 98.62  (76.34 - 125.7) | -1.99  (-4 - 0.05) |
| **Puerto Rico** | 3.48  (3.09 - 3.91) | 0.64  (0.48 - 0.84) | 120.05  (106.52 - 134.76) | 50.88  (38.37 - 66.26) | -7.1  (-10.53 - -3.54) |
| **Saint Kitts and Nevis** | 5.53  (4.7 - 6.46) | 1.89  (1.47 - 2.42) | 1.95  (1.65 - 2.3) | 1.09  (0.82 - 1.41) | -4.15  (-6.48 - -1.76) |
| **Saint Lucia** | 5.42  (4.74 - 6.14) | 2.34  (1.9 - 2.84) | 4.43  (3.83 - 5.01) | 4.8  (3.88 - 5.85) | -3.74  (-5.96 - -1.46) |
| **Saint Vincent and the Grenadines** | 7.75  (6.63 - 8.91) | 4.08  (3.31 - 4.92) | 5.47  (4.67 - 6.29) | 5.23  (4.22 - 6.33) | -2.53  (-4.28 - -0.74) |
| **Suriname** | 8.45  (7.33 - 9.57) | 4.69  (3.75 - 5.76) | 21.65  (18.85 - 24.5) | 26.72  (21.36 - 32.98) | -2.46  (-4.02 - -0.87) |
| **Trinidad and Tobago** | 8.57  (7.76 - 9.56) | 4.19  (3.03 - 5.67) | 65.99  (59.48 - 73.61) | 73.94  (53.22 - 100.12) | -2.93  (-4.54 - -1.29) |
| **United States Virgin Islands** | 3.2  (2.61 - 3.89) | 1.77  (1.36 - 2.22) | 2.41  (1.96 - 2.93) | 3.02  (2.32 - 3.83) | -2.13  (-4.84 - 0.66) |
| **Central Latin America** | 9.36  (8.68 - 9.78) | 3.48  (3.02 - 4.05) | 7143.28  (6720.99 - 7415.57) | 7857.15  (6818.87 - 9138.87) | -3.96  (-5.67 - -2.22) |
| **Colombia** | 8.17  (7.45 - 8.72) | 1.91  (1.44 - 2.52) | 1306.65  (1208.82 - 1388.22) | 1047.77  (790.6 - 1384.15) | -5.68  (-7.72 - -3.59) |
| **Costa Rica** | 4.05  (3.46 - 4.68) | 2.04  (1.52 - 2.7) | 67.27  (58.24 - 77.59) | 104.76  (78.04 - 138.01) | -3.6  (-5.9 - -1.24) |
| **El Salvador** | 14.79  (13.37 - 16.09) | 4.39  (3.31 - 5.69) | 434.62  (395.35 - 471.87) | 279.84  (210.9 - 362.54) | -5.27  (-6.73 - -3.78) |
| **Guatemala** | 20.78  (17.96 - 23.36) | 9.82  (7.82 - 12.26) | 700.89  (612.11 - 786.28) | 1010.36  (798.31 - 1278.41) | -3.14  (-4.11 - -2.15) |
| **Honduras** | 14.06  (10.65 - 16.86) | 11.69  (8.6 - 15.61) | 312.86  (252.74 - 368.26) | 622.91  (454.04 - 820.63) | -0.62  (-1.73 - 0.51) |
| **Mexico** | 9.97  (9.14 - 10.4) | 3.68  (3.11 - 4.26) | 3709.72  (3473.17 - 3847.76) | 3930.92  (3318 - 4565.33) | -4.01  (-5.7 - -2.29) |
| **Nicaragua** | 6.32  (5.4 - 7.09) | 3.11  (2.51 - 3.92) | 93.83  (81.59 - 104.64) | 116.45  (93.69 - 149.37) | -2.85  (-4.61 - -1.04) |
| **Panama** | 3.82  (3.32 - 4.32) | 1.2  (0.9 - 1.61) | 55.41  (48.35 - 62.3) | 51.18  (38.5 - 68.22) | -4.19  (-6.89 - -1.4) |
| **Venezuela (Bolivarian Republic of)** | 5.08  (4.5 - 5.55) | 2.52  (1.9 - 3.35) | 462.04  (416.27 - 501.78) | 692.94  (520.98 - 926.74) | -3.21  (-5.38 - -0.99) |
| **Tropical Latin America** | 6.02  (5.58 - 6.35) | 2.26  (2.03 - 2.49) | 5073.39  (4768.67 - 5326.43) | 5285.7  (4772.4 - 5823.26) | -3.62  (-5.76 - -1.44) |
| **Brazil** | 6.07  (5.62 - 6.41) | 2.26  (2.03 - 2.5) | 4983.39  (4679.5 - 5237.35) | 5167.4  (4673 - 5683.21) | -3.66  (-5.79 - -1.48) |
| **Paraguay** | 4.2  (3.61 - 4.8) | 2.23  (1.66 - 3) | 90.01  (78.14 - 102.33) | 118.3  (88.43 - 158.48) | -2.29  (-4.52 - 0) |
| **North Africa and Middle East** | 5.84  (4.7 - 7.65) | 2.58  (2.14 - 3.09) | 9068.5  (7606.49 - 11382.69) | 9604.47  (7924.68 - 11651.28) | -2.76  (-4.8 - -0.67) |
| **Afghanistan** | 19.48  (13.12 - 27.86) | 11.52  (7.66 - 15.98) | 1221.67  (818.89 - 1709.48) | 1310.87  (877.84 - 1825.63) | -1.85  (-2.84 - -0.84) |
| **Algeria** | 8.59  (6.55 - 11.3) | 3.14  (2.34 - 4.01) | 716.6  (537.96 - 957.26) | 764.58  (567.65 - 996.17) | -3.48  (-5.27 - -1.64) |
| **Bahrain** | 15.55  (13.05 - 18.71) | 6.1  (4.93 - 7.51) | 14.02  (11.75 - 16.67) | 19.74  (15.75 - 24.46) | -4.02  (-5.17 - -2.85) |
| **Egypt** | 4.92  (4.3 - 6.13) | 2.38  (1.33 - 3.51) | 1454.18  (1286.78 - 1684.25) | 1435.9  (775.08 - 2193.22) | -2.05  (-4.25 - 0.21) |
| **Iran (Islamic Republic of)** | 6.53  (5.16 - 8.23) | 2.2  (1.89 - 2.49) | 1342.73  (1138.04 - 1611.27) | 1380.88  (1188.37 - 1550.66) | -3.65  (-5.75 - -1.51) |
| **Iraq** | 3.04  (2.34 - 3.99) | 1.05  (0.76 - 1.31) | 234.24  (180.8 - 309.99) | 208.44  (152.54 - 263.96) | -3.74  (-6.68 - -0.7) |
| **Jordan** | 6.68  (5 - 8.32) | 1.84  (1.47 - 2.25) | 62.89  (47.5 - 78.41) | 78.03  (61.86 - 96.02) | -4.74  (-6.78 - -2.65) |
| **Kuwait** | 2.98  (2.48 - 3.51) | 1.23  (0.94 - 1.59) | 13.56  (11.57 - 15.82) | 23.4  (18.08 - 30.48) | -2.53  (-5.54 - 0.57) |
| **Lebanon** | 5.38  (3.68 - 7.46) | 2.17  (1.2 - 3.73) | 97.94  (65.49 - 137.38) | 108.88  (60.06 - 185.31) | -3  (-5.17 - -0.77) |
| **Libya** | 6.07  (3.69 - 9.12) | 2.75  (1.75 - 3.96) | 104.39  (66.49 - 152.25) | 121.92  (78.2 - 174.35) | -2.84  (-4.92 - -0.72) |
| **Morocco** | 8.49  (5.63 - 12.42) | 4.04  (2.92 - 5.28) | 977.28  (695.17 - 1368.12) | 1002.33  (746.71 - 1275.8) | -2.64  (-4.32 - -0.93) |
| **Oman** | 5.91  (4.14 - 8.35) | 2.3  (1.82 - 2.88) | 30.38  (21.14 - 43.18) | 22.33  (17.85 - 27.78) | -2.74  (-4.91 - -0.51) |
| **Palestine** | 3.3  (2.09 - 4.7) | 1.65  (1.34 - 2.01) | 24.93  (15.78 - 36.6) | 27.85  (22.81 - 33.57) | -2.67  (-5.39 - 0.12) |
| **Qatar** | 3.45  (2.6 - 4.46) | 1.83  (1.4 - 2.4) | 2.09  (1.59 - 2.85) | 4.72  (3.49 - 6.41) | -1.52  (-3.95 - 0.98) |
| **Saudi Arabia** | 5.71  (3.89 - 8.95) | 1.9  (1.43 - 2.84) | 258.79  (178.09 - 400.46) | 207.49  (155.47 - 270.86) | -3.62  (-5.81 - -1.37) |
| **Sudan** | 8.36  (4.32 - 14.88) | 4.53  (2.43 - 7.4) | 725.32  (412.32 - 1212.68) | 734.37  (409.89 - 1183.17) | -2.01  (-3.62 - -0.37) |
| **Syrian Arab Republic** | 2.35  (1.68 - 3.05) | 0.96  (0.7 - 1.27) | 124.38  (94.57 - 157.02) | 87.29  (61.43 - 118.31) | -3.64  (-6.93 - -0.22) |
| **Tunisia** | 5.07  (3.55 - 6.69) | 2.26  (1.57 - 3.11) | 203.66  (141.08 - 273.23) | 244.6  (169.48 - 340.37) | -2.83  (-4.99 - -0.62) |
| **Turkey** | 2.45  (1.95 - 2.97) | 1.11  (0.88 - 1.38) | 841.75  (670.09 - 1032.19) | 903.96  (713.18 - 1125.16) | -2.47  (-5.71 - 0.9) |
| **United Arab Emirates** | 6.93  (4.96 - 9.6) | 2.65  (1.84 - 3.77) | 18.12  (12.5 - 27.82) | 49.16  (31.45 - 72.88) | -3.12  (-4.86 - -1.36) |
| **Yemen** | 13.82  (8.03 - 24.6) | 7.61  (5.16 - 11.67) | 593.46  (371.72 - 1006.85) | 857.95  (584.65 - 1291.4) | -2.44  (-3.7 - -1.16) |
| **South Asia** | 17.89  (15.36 - 20.23) | 5.92  (5.01 - 7.06) | 95676.57  (82757.86 - 108458.06) | 74928.7  (63469.58 - 89532.4) | -4.18  (-5.38 - -2.97) |
| **Bangladesh** | 17.54  (13.28 - 22.08) | 1.23  (0.89 - 1.63) | 8227.45  (6179.28 - 10385.4) | 1485.72  (1031.56 - 1980.77) | -10.08  (-11.83 - -8.29) |
| **Bhutan** | 21.15  (13.82 - 33.36) | 7.39  (3.8 - 13.4) | 50.28  (31.35 - 82.01) | 36.86  (18.53 - 69.15) | -3.96  (-5.09 - -2.8) |
| **India** | 20.17  (17.29 - 23.36) | 6.7  (5.6 - 8.12) | 83246.05  (71758.19 - 95626.37) | 69081.92  (57544.78 - 83349.17) | -4.16  (-5.29 - -3.02) |
| **Nepal** | 3.52  (2.38 - 4.97) | 1.51  (1.03 - 2.32) | 227.05  (158 - 317.73) | 229.2  (157.17 - 357.25) | -3.25  (-5.96 - -0.46) |
| **Pakistan** | 7.68  (5.17 - 12.32) | 4.66  (3.42 - 6.49) | 3925.73  (2677.8 - 6149.6) | 4095  (3041.04 - 5658.66) | -1.84  (-3.42 - -0.24) |
| **East Asia** | 7.65  (6.52 - 9.24) | 2.4  (2.05 - 2.77) | 58107.81  (48826.76 - 71277.06) | 42947.82  (36862.23 - 50139.21) | -3.72  (-5.59 - -1.81) |
| **China** | 7.53  (6.35 - 9.12) | 2.33  (1.97 - 2.73) | 55475.62  (46109.32 - 68801.52) | 40134.28  (33951.45 - 47318.02) | -3.71  (-5.6 - -1.78) |
| **Democratic People's Republic of Korea** | 9.61  (6.93 - 12.62) | 6.53  (4.24 - 8.89) | 1316.27  (945.28 - 1778.9) | 1936.56  (1215.11 - 2655.87) | -1.24  (-2.61 - 0.16) |
| **Taiwan (Province of China)** | 11.03  (10.04 - 12) | 2.17  (1.67 - 2.78) | 1315.92  (1215.8 - 1419.12) | 876.99  (676.24 - 1121.81) | -6.43  (-8.17 - -4.67) |
| **Oceania** | 11.48  (8.76 - 14.13) | 7.23  (5.8 - 8.96) | 327.97  (251.02 - 403.8) | 477.45  (382.93 - 601.47) | -1.63  (-2.96 - -0.27) |
| **American Samoa** | 8.96  (7.4 - 10.86) | 4.56  (3.68 - 5.45) | 1.71  (1.42 - 2.05) | 1.89  (1.52 - 2.25) | -2.69  (-4.32 - -1.04) |
| **Cook Islands** | 3.74  (2.94 - 4.72) | 1.28  (1.02 - 1.6) | 0.4  (0.31 - 0.52) | 0.29  (0.23 - 0.37) | -4.07  (-6.79 - -1.27) |
| **Fiji** | 9.96  (7.78 - 12.67) | 5.92  (4.55 - 7.55) | 32.17  (24.65 - 40.75) | 35.77  (26.74 - 46.63) | -1.95  (-3.41 - -0.46) |
| **Guam** | 4.67  (3.9 - 5.58) | 1.45  (1.09 - 1.82) | 2.39  (2 - 2.86) | 2.69  (2.03 - 3.39) | -4.95  (-7.62 - -2.19) |
| **Kiribati** | 36.02  (25.91 - 47.18) | 21.78  (16.38 - 27.96) | 12.22  (8.54 - 15.99) | 12.4  (8.62 - 17.08) | -1.87  (-2.62 - -1.11) |
| **Marshall Islands** | 21.04  (14.91 - 27.36) | 10.82  (7.22 - 14.78) | 3.26  (2.32 - 4.3) | 2.93  (1.87 - 4.16) | -2.35  (-3.38 - -1.31) |
| **Micronesia (Federated States of)** | 22.71  (17.11 - 29.98) | 10.48  (6.89 - 14.6) | 9.64  (7.15 - 12.86) | 5.9  (3.61 - 8.48) | -2.99  (-4.04 - -1.92) |
| **Nauru** | 17.3  (11.98 - 24.35) | 10.13  (7.53 - 13.39) | 0.57  (0.38 - 0.88) | 0.34  (0.22 - 0.48) | -1.73  (-2.76 - -0.69) |
| **Niue** | 10.4  (7.76 - 13.76) | 5.13  (3.86 - 6.47) | 0.24  (0.18 - 0.32) | 0.11  (0.08 - 0.13) | -2.72  (-4.18 - -1.22) |
| **Northern Mariana Islands** | 7.59  (6.14 - 9.41) | 3.61  (2.92 - 4.3) | 0.97  (0.76 - 1.25) | 1.35  (1.08 - 1.63) | -2.56  (-4.31 - -0.78) |
| **Palau** | 7.62  (5.11 - 10.85) | 4.76  (3.06 - 6.21) | 0.63  (0.42 - 0.93) | 0.75  (0.46 - 0.99) | -1.59  (-3.26 - 0.11) |
| **Papua New Guinea** | 10.03  (7.32 - 12.84) | 7.1  (5.28 - 9.2) | 189.99  (137.86 - 244.67) | 324.91  (245.37 - 431.27) | -1.16  (-2.54 - 0.24) |
| **Samoa** | 14.84  (10.04 - 21.82) | 8.16  (6.16 - 10.48) | 11.79  (7.82 - 17.26) | 10.64  (7.95 - 13.73) | -2.08  (-3.31 - -0.84) |
| **Solomon Islands** | 16.24  (10.6 - 22.67) | 9.51  (7.27 - 12.15) | 22.64  (14.15 - 32.68) | 28.25  (21.02 - 37.31) | -1.78  (-2.93 - -0.63) |
| **Tokelau** | 12.95  (8.76 - 18.04) | 5.72  (4.37 - 7.29) | 0.17  (0.11 - 0.24) | 0.07  (0.05 - 0.09) | -2.94  (-4.3 - -1.56) |
| **Tonga** | 16.37  (13.03 - 19.91) | 8.87  (6.67 - 11.42) | 7.91  (6.26 - 9.63) | 6.84  (5.13 - 8.81) | -2.23  (-3.39 - -1.06) |
| **Tuvalu** | 17.83  (11.77 - 24.91) | 8.43  (5.92 - 11.4) | 1.08  (0.7 - 1.54) | 0.74  (0.51 - 1.03) | -2.6  (-3.77 - -1.42) |
| **Vanuatu** | 20.92  (12.42 - 32.21) | 12.81  (8.82 - 17.51) | 12.04  (7.11 - 18.2) | 19.05  (13.25 - 26.61) | -2.06  (-3.04 - -1.07) |
| **Southeast Asia** | 8.68  (7.64 - 9.7) | 3.74  (3.32 - 4.23) | 20259.71  (17980 - 22454.11) | 19407.83  (17284.9 - 22253.12) | -3.19  (-4.84 - -1.51) |
| **Cambodia** | 46.22  (36.82 - 55.22) | 22.48  (17.42 - 28.98) | 1884.07  (1481.99 - 2262.09) | 2163.89  (1654.07 - 2839.64) | -2.69  (-3.38 - -2) |
| **Indonesia** | 2.82  (2.23 - 3.37) | 1.45  (1.11 - 1.7) | 2237.09  (1886.56 - 2614.97) | 1990.08  (1548.34 - 2293.85) | -2.29  (-5.02 - 0.52) |
| **Lao People's Democratic Republic** | 44.14  (33.67 - 57.63) | 19.24  (14.04 - 26.14) | 844.09  (646.5 - 1070.03) | 705.3  (498.02 - 978.01) | -3.14  (-3.87 - -2.4) |
| **Malaysia** | 7.7  (6.59 - 9.12) | 6.25  (4.76 - 8.02) | 603.09  (519.27 - 712.46) | 1315.71  (989.43 - 1681.86) | -1.98  (-3.33 - -0.61) |
| **Maldives** | 6.6  (4 - 9.2) | 1.59  (1.23 - 1.98) | 4.25  (2.35 - 6.18) | 3.76  (2.95 - 4.66) | -5.82  (-8.06 - -3.52) |
| **Mauritius** | 7.85  (7.11 - 8.62) | 2.12  (1.67 - 2.63) | 53.75  (48.57 - 59.33) | 34  (26.57 - 42.45) | -4.7  (-6.76 - -2.6) |
| **Myanmar** | 23.55  (18.24 - 29.56) | 6.85  (5.6 - 8.7) | 5578.46  (4261.95 - 7163.57) | 2945  (2366.86 - 3868.86) | -4.29  (-5.37 - -3.19) |
| **Philippines** | 29.54  (25.02 - 35.29) | 11.24  (9.36 - 13.32) | 7161.31  (6229.28 - 8605.96) | 7605.62  (6262.71 - 9126.05) | -3.65  (-4.59 - -2.71) |
| **Seychelles** | 15.1  (12.94 - 17.45) | 7.52  (6.16 - 8.9) | 8.44  (7.26 - 9.76) | 7.33  (5.97 - 8.7) | -2.67  (-3.9 - -1.43) |
| **Sri Lanka** | 1.65  (1.37 - 1.93) | 0.46  (0.32 - 0.64) | 151.9  (126.44 - 179.27) | 96.31  (67.2 - 138.85) | -3.74  (-8.09 - 0.82) |
| **Thailand** | 2.3  (1.84 - 2.85) | 1.71  (1.26 - 2.4) | 690.06  (561.7 - 841.57) | 1667.89  (1225.47 - 2326.24) | -0.53  (-3.58 - 2.61) |
| **Timor-Leste** | 30.34  (21.39 - 44.3) | 17.74  (12.11 - 26.72) | 78.99  (55.61 - 118.14) | 120.52  (80.65 - 184.57) | -2.22  (-3.07 - -1.37) |
| **Viet Nam** | 2.78  (2.02 - 3.71) | 1  (0.72 - 1.39) | 937.29  (690.39 - 1230.86) | 726.99  (523.46 - 1002.68) | -3.99  (-7.11 - -0.76) |
| **Central Sub-Saharan Africa** | 8.4  (5.41 - 11.35) | 5.84  (3.58 - 8.29) | 1962.73  (1491.23 - 2479.79) | 2998.08  (2139.49 - 3996.97) | -1.07  (-2.56 - 0.45) |
| **Angola** | 10.01  (5.59 - 14.76) | 6.17  (3.61 - 8.89) | 427.81  (270.79 - 592.41) | 636.32  (420.35 - 899.34) | -1.6  (-3.02 - -0.16) |
| **Central African Republic** | 15.01  (9.02 - 22.48) | 13.69  (7.95 - 20.84) | 192.55  (127.34 - 284.2) | 309.19  (183.61 - 475.09) | -0.27  (-1.34 - 0.81) |
| **Congo** | 9.16  (6.25 - 12.49) | 5.34  (3.59 - 7.85) | 95.55  (69.67 - 125.27) | 121.48  (80.82 - 176.59) | -1.71  (-3.19 - -0.2) |
| **Democratic Republic of the Congo** | 7.45  (4.63 - 10.18) | 5.43  (3.08 - 8.06) | 1186.76  (872.73 - 1544.22) | 1879.52  (1248.95 - 2624.68) | -0.84  (-2.4 - 0.74) |
| **Equatorial Guinea** | 11.76  (6.9 - 18.21) | 3.61  (1.97 - 5.7) | 24.07  (14.66 - 36.83) | 16.04  (8.97 - 25.58) | -4.78  (-6.37 - -3.16) |
| **Gabon** | 6.94  (4.45 - 11.5) | 3.89  (2.34 - 5.78) | 35.99  (25.75 - 57.92) | 35.54  (23.25 - 52.24) | -1.76  (-3.47 - -0.03) |
| **Eastern Sub-Saharan Africa** | 7.3  (5.31 - 10.2) | 4.12  (2.61 - 5.86) | 6111.93  (4515.18 - 8535.17) | 7324.29  (4936.06 - 9941.71) | -2.08  (-3.77 - -0.35) |
| **Burundi** | 10.2  (6.88 - 17.23) | 7.29  (3.78 - 12.66) | 268.66  (177 - 455.31) | 371.58  (204.81 - 635.65) | -1.44  (-2.84 - -0.02) |
| **Comoros** | 7.55  (3.44 - 12.3) | 4.53  (2.22 - 7.2) | 17.37  (7.29 - 28.77) | 21.83  (10.96 - 34.47) | -1.96  (-3.59 - -0.31) |
| **Djibouti** | 6.36  (2.99 - 10.56) | 4.05  (1.63 - 6.98) | 10.66  (4.96 - 18.81) | 24.66  (10.3 - 43.45) | -1.65  (-3.35 - 0.08) |
| **Eritrea** | 8.2  (5.09 - 11.76) | 6.6  (4.59 - 9.3) | 100.02  (61.61 - 144.96) | 194.45  (134.96 - 273.94) | -0.66  (-2.13 - 0.82) |
| **Ethiopia** | 10.43  (6.81 - 16.31) | 3.36  (1.78 - 4.98) | 2425.91  (1526.99 - 3839.07) | 1506.86  (829.31 - 2220.02) | -4.14  (-5.75 - -2.51) |
| **Kenya** | 6.81  (4.08 - 11.79) | 4.87  (2.68 - 7.3) | 592.63  (370.86 - 1025.92) | 1111.61  (631.83 - 1653.71) | -0.92  (-2.56 - 0.76) |
| **Madagascar** | 4.81  (2.64 - 7.33) | 4.06  (2.73 - 5.69) | 291.16  (172.62 - 420.04) | 478.12  (330.74 - 644.42) | -0.42  (-2.38 - 1.57) |
| **Malawi** | 5.83  (3.75 - 7.74) | 3.95  (2.26 - 5.93) | 249.34  (170.13 - 334.37) | 322.9  (193.47 - 470.64) | -1.5  (-3.29 - 0.32) |
| **Mozambique** | 4.99  (3.09 - 6.8) | 4.74  (3.06 - 7.14) | 315.97  (207.38 - 433.51) | 605.23  (399.39 - 885.55) | 0.4  (-1.46 - 2.3) |
| **Rwanda** | 14.46  (10.7 - 20.15) | 6.2  (3.8 - 10.29) | 446.36  (326.06 - 635.43) | 369.02  (233.19 - 596.38) | -3.9  (-5.15 - -2.64) |
| **Somalia** | 8.49  (3.66 - 14.08) | 7.3  (3.4 - 12.31) | 244.19  (109.92 - 411.07) | 556.04  (280.36 - 952.76) | -0.52  (-1.94 - 0.92) |
| **South Sudan** | 5.54  (2.65 - 9.5) | 4.24  (2.15 - 7.53) | 144.89  (69.5 - 260.49) | 168.34  (87.73 - 314.87) | -0.88  (-2.7 - 0.97) |
| **Uganda** | 6.16  (3.95 - 9.44) | 4.68  (3.17 - 7.9) | 416.84  (271.17 - 624.3) | 718.24  (505.61 - 1173.66) | -1.37  (-3.02 - 0.3) |
| **United Republic of Tanzania** | 3.19  (2.15 - 4.54) | 2.33  (1.18 - 3.69) | 370.18  (266.74 - 515.45) | 609.39  (328.56 - 938.73) | -0.87  (-3.27 - 1.59) |
| **Zambia** | 6.54  (4.79 - 8.86) | 3.34  (2.5 - 4.63) | 213.27  (161.88 - 283.55) | 260.18  (194.79 - 359.34) | -2.56  (-4.26 - -0.83) |
| **Southern Sub-Saharan Africa** | 5.54  (3.94 - 7.2) | 4.95  (4.44 - 5.54) | 1487.46  (1063.12 - 1911) | 2512.56  (2241.21 - 2852.41) | -0.02  (-1.67 - 1.65) |
| **Botswana** | 11.11  (7.61 - 16.19) | 6.82  (4.93 - 9.46) | 56.66  (36.74 - 85.43) | 80.62  (55.67 - 117.15) | -1.88  (-3.13 - -0.61) |
| **Eswatini** | 7.95  (5.09 - 11.87) | 7.4  (5.12 - 10.27) | 20.74  (12.79 - 31.9) | 36.32  (24.14 - 52.12) | 0.27  (-1.1 - 1.66) |
| **Lesotho** | 10.9  (6.58 - 15.73) | 12.72  (9.51 - 16.75) | 97.64  (56.82 - 144.84) | 147.89  (107.05 - 200.11) | 1.19  (0.04 - 2.36) |
| **Namibia** | 10.32  (7.67 - 12.92) | 6.67  (5.01 - 8.96) | 65.06  (47.16 - 83.16) | 83.26  (60.93 - 113.64) | -1.43  (-2.78 - -0.06) |
| **South Africa** | 4.59  (3.2 - 6.09) | 4.02  (3.55 - 4.5) | 956.02  (672.22 - 1246.74) | 1573.55  (1391.84 - 1762.07) | -0.05  (-1.84 - 1.76) |
| **Zimbabwe** | 7.63  (5.35 - 10.6) | 8.67  (5.12 - 11.95) | 291.33  (205.12 - 407.11) | 590.92  (326.42 - 838.48) | 0.86  (-0.54 - 2.28) |
| **Western Sub-Saharan Africa** | 9.38  (7.17 - 12.76) | 6.69  (5.06 - 8.6) | 8337.95  (6373.25 - 11429.88) | 12672.22  (9246.69 - 16900.72) | -0.87  (-2.29 - 0.57) |
| **Benin** | 9.04  (6.22 - 12.64) | 7.11  (5.47 - 9.11) | 194.5  (134.26 - 270.94) | 353.01  (260.5 - 468.39) | -0.61  (-2.05 - 0.85) |
| **Burkina Faso** | 5.4  (4.35 - 6.57) | 4.76  (2.82 - 7.5) | 220.5  (173.95 - 275.48) | 446.2  (269.93 - 706.6) | -0.12  (-1.97 - 1.75) |
| **Cabo Verde** | 8.6  (6.44 - 11.2) | 2.78  (2.13 - 3.57) | 21.01  (15.79 - 27.21) | 12.02  (9.24 - 15.36) | -4.57  (-6.59 - -2.5) |
| **Cameroon** | 9.32  (7.34 - 11.35) | 5.92  (3.82 - 8.05) | 400.52  (319.4 - 488.69) | 690.58  (429.69 - 970.55) | -1.46  (-2.88 - -0.03) |
| **Chad** | 11.5  (7.65 - 15.42) | 9.83  (7.38 - 12.67) | 336.85  (228.39 - 450.7) | 594.59  (447.42 - 777.25) | -0.41  (-1.61 - 0.8) |
| **Cote d'Ivoire** | 8.54  (5.94 - 11.36) | 6.43  (4.86 - 8.27) | 359.07  (249.84 - 494.75) | 677.14  (491.49 - 891.09) | -1  (-2.4 - 0.42) |
| **Gambia** | 10.98  (7.84 - 14.69) | 6.96  (4.91 - 9.18) | 40.06  (27.42 - 55.05) | 66.44  (46.94 - 87.85) | -1.7  (-3.05 - -0.32) |
| **Ghana** | 3.51  (2.39 - 5.34) | 2.15  (1.62 - 2.84) | 217.23  (149.56 - 315.92) | 339.06  (248.38 - 449.11) | -1.35  (-3.67 - 1.04) |
| **Guinea** | 9.23  (6.2 - 13.2) | 7.41  (5.01 - 9.91) | 312.35  (209.46 - 437.99) | 432.24  (287.02 - 585.27) | -0.41  (-1.8 - 1) |
| **Guinea-Bissau** | 16  (12.02 - 20.76) | 11.49  (8.46 - 15.2) | 69.43  (50.59 - 91.09) | 84.54  (61.15 - 113.14) | -0.8  (-1.89 - 0.3) |
| **Liberia** | 9.37  (6.29 - 13.35) | 6.56  (4.52 - 9.23) | 102.26  (70.08 - 145.49) | 128.54  (84.04 - 185.81) | -1.26  (-2.7 - 0.2) |
| **Mali** | 10.19  (6.4 - 14.86) | 8.12  (4.95 - 12.25) | 425.87  (264.72 - 619.75) | 732.22  (435.41 - 1126.59) | -0.43  (-1.81 - 0.97) |
| **Mauritania** | 10.31  (7.11 - 13) | 4.44  (3.13 - 5.78) | 101.38  (69.63 - 127.72) | 87.79  (59.6 - 116.54) | -2.77  (-4.32 - -1.2) |
| **Niger** | 11.68  (7.38 - 16.11) | 10.17  (5.89 - 14.9) | 365.4  (228.76 - 515.8) | 822.31  (471.76 - 1242.75) | -0.25  (-1.51 - 1.02) |
| **Nigeria** | 10.26  (6.57 - 16.1) | 7.1  (4.19 - 12.01) | 4562.68  (2883.36 - 7463.81) | 6175.68  (3460.7 - 10864.42) | -0.89  (-2.24 - 0.49) |
| **Sao Tome and Principe** | 5.93  (4.34 - 7.95) | 3.16  (2.13 - 5.25) | 3.52  (2.53 - 4.75) | 2.93  (2 - 4.89) | -2.11  (-3.9 - -0.29) |
| **Senegal** | 9.42  (5.86 - 13.13) | 6.53  (4.06 - 9.65) | 317.52  (199.49 - 444.81) | 477.82  (289.47 - 701.31) | -1.13  (-2.59 - 0.35) |
| **Sierra Leone** | 8.48  (5.55 - 12.09) | 7.69  (5.12 - 10.77) | 169.17  (112.56 - 241.23) | 289.46  (189.99 - 413.77) | 0.28  (-1.16 - 1.75) |
| **Togo** | 9.05  (6.46 - 11.84) | 7.53  (5.34 - 10) | 118.35  (85.49 - 156.3) | 259.5  (176.09 - 349.91) | -0.21  (-1.63 - 1.22) |
| **High SDI^§^** | 3.81  (3.51 - 3.97) | 1.18  (1.04 - 1.29) | 39550.24  (36627.02 - 41143.73) | 25352.27  (21967.38 - 28013.26) | -4.6  (-7.37 - -1.74) |
| **High-middle SDI** | 4.62  (4.31 - 4.96) | 1.98  (1.81 - 2.12) | 45424.84  (42411.02 - 48924.08) | 38952.34  (35625.27 - 41836.15) | -3.13  (-5.38 - -0.82) |
| **Middle SDI** | 7.29  (6.58 - 8.02) | 2.78  (2.51 - 3.07) | 65811.65  (59442.71 - 72958.41) | 59505.13  (54129.84 - 66053.55) | -3.38  (-5.23 - -1.49) |
| **Low-middle SDI** | 16.87  (15.01 - 18.61) | 6.52  (5.77 - 7.5) | 97950.63  (87257.27 - 108104.25) | 81310.92  (72345.72 - 93691.68) | -3.49  (-4.7 - -2.27) |
| **Low SDI** | 12.74  (10.96 - 14.57) | 6.15  (5.31 - 7.04) | 30107.46  (25922.55 - 34410.47) | 30883.7  (27174.12 - 35016.22) | -2.67  (-3.98 - -1.33) |

*Age-standardized rate (per 100,000 population)

¶ Estimated annual percentage change

§ Socio-demographic index

**Additional file 1:**

**Figure S1. Incident cases with age-standardized incidence rate (per 100,000 population) changes in all years from 1990 to 2019**





UI=uncertainty intervals.

**Additional file 1:**

**Figure S2. DALYs with age-standardized rate (per 100,000 population) changes in all years from 1990 to 2019**





DALYs=disability-adjusted life years, UI=uncertainty intervals.

**Additional file 1:**

**Figure S3. Deaths with age-standardized death rate (per 100,000 population) changes by age in 2019**





UI=uncertainty intervals.

**Additional file 1:**

**Figure S4. DALYs and age-standardized DALY rate (per 100,000 population) changes by age in 2019**





DALYs=disability-adjusted life years, UI=uncertainty intervals.

**Additional file 1:**

**Figure S5. Age-standardized prevalent rate changes in PUD in seven super-regions in all years from 1990 to 2019**


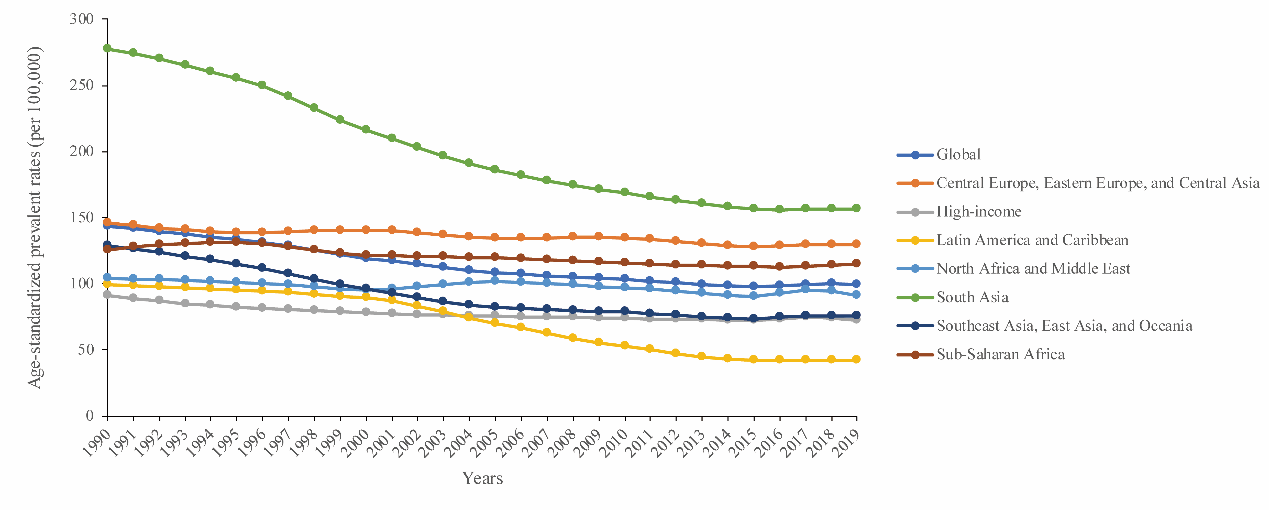


PUD=peptic ulcer disease.

**Additional file 1:**

**Figure S6. Trends of age-standardized incidence rates (per 100,000 population) in seven super-regions in all years from 1990 to 2019**


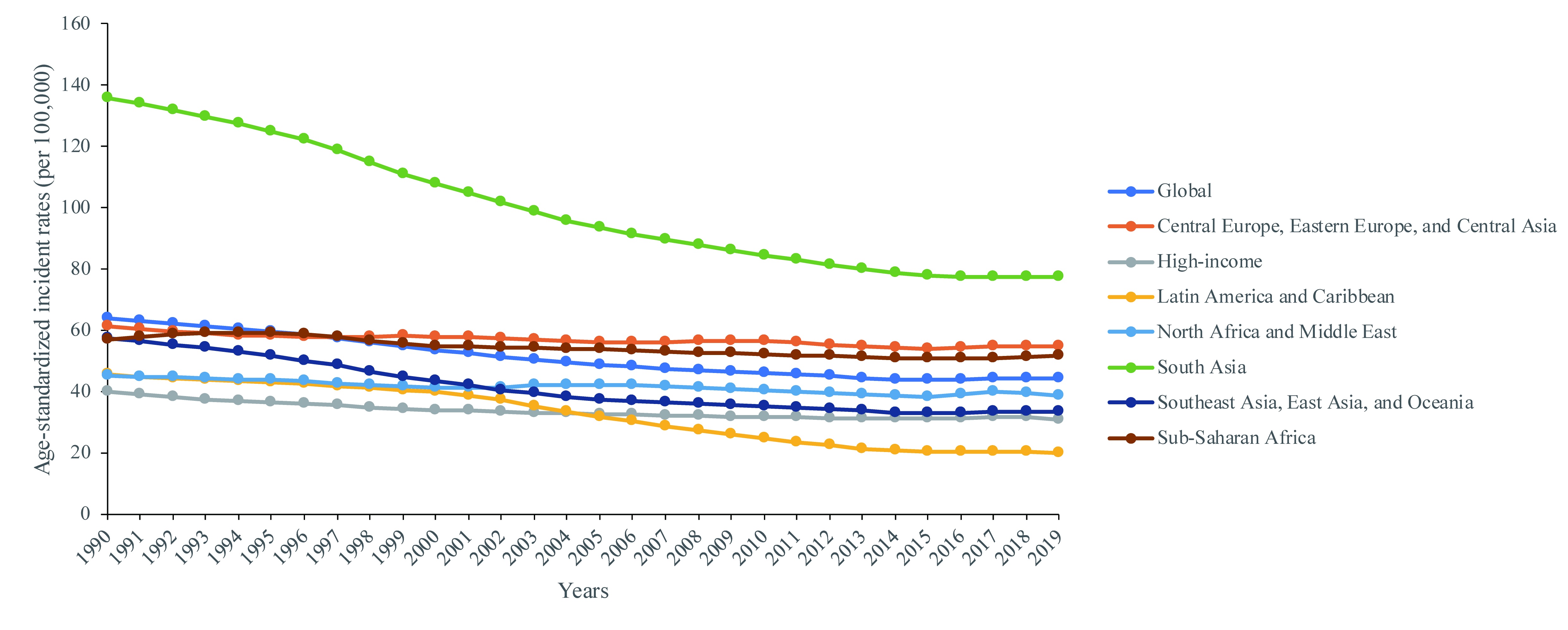


**Additional file 1:**

**Figure S7. Trends of age-standardized death rates (per 100,000 population) in seven super-regions in all years from 1990 to 2019**


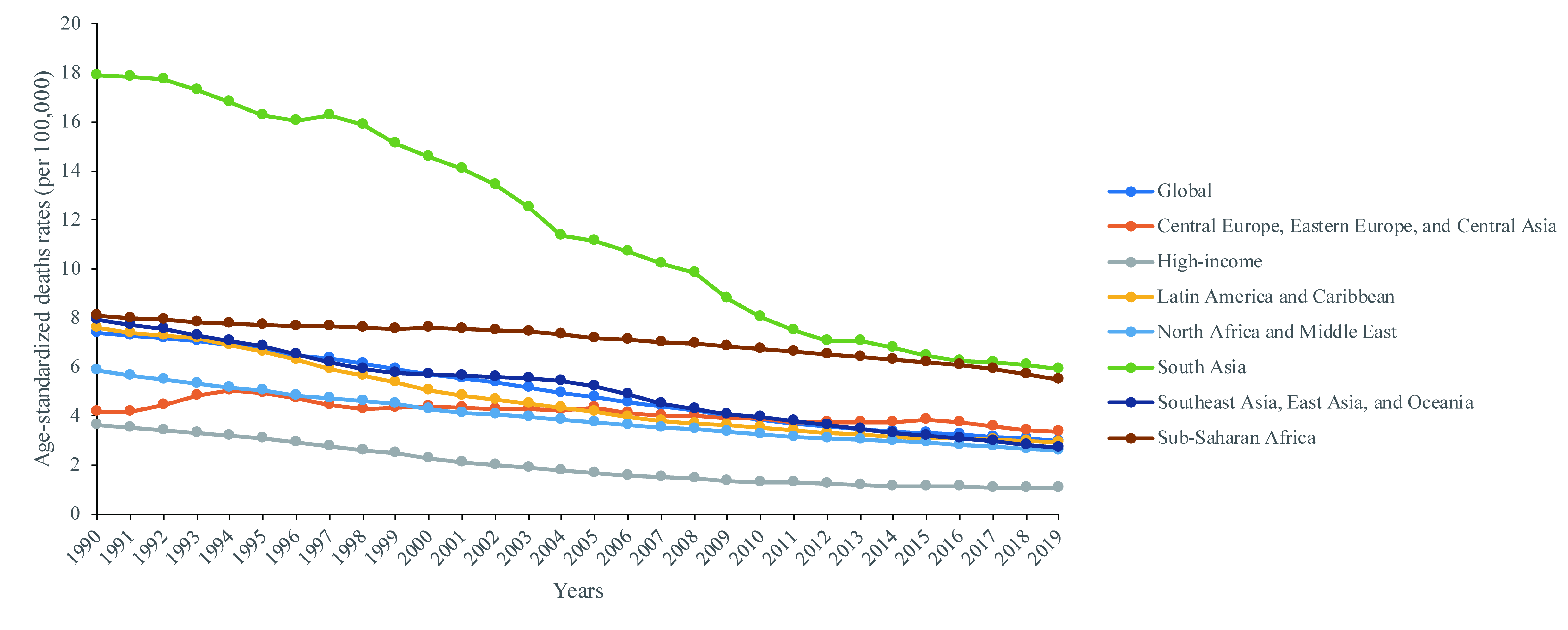


**Additional file 1:**

**Figure S8. Trends of age-standardized DALY rates (per 100,000 population) in seven super-regions in all years from 1990 to 2019**


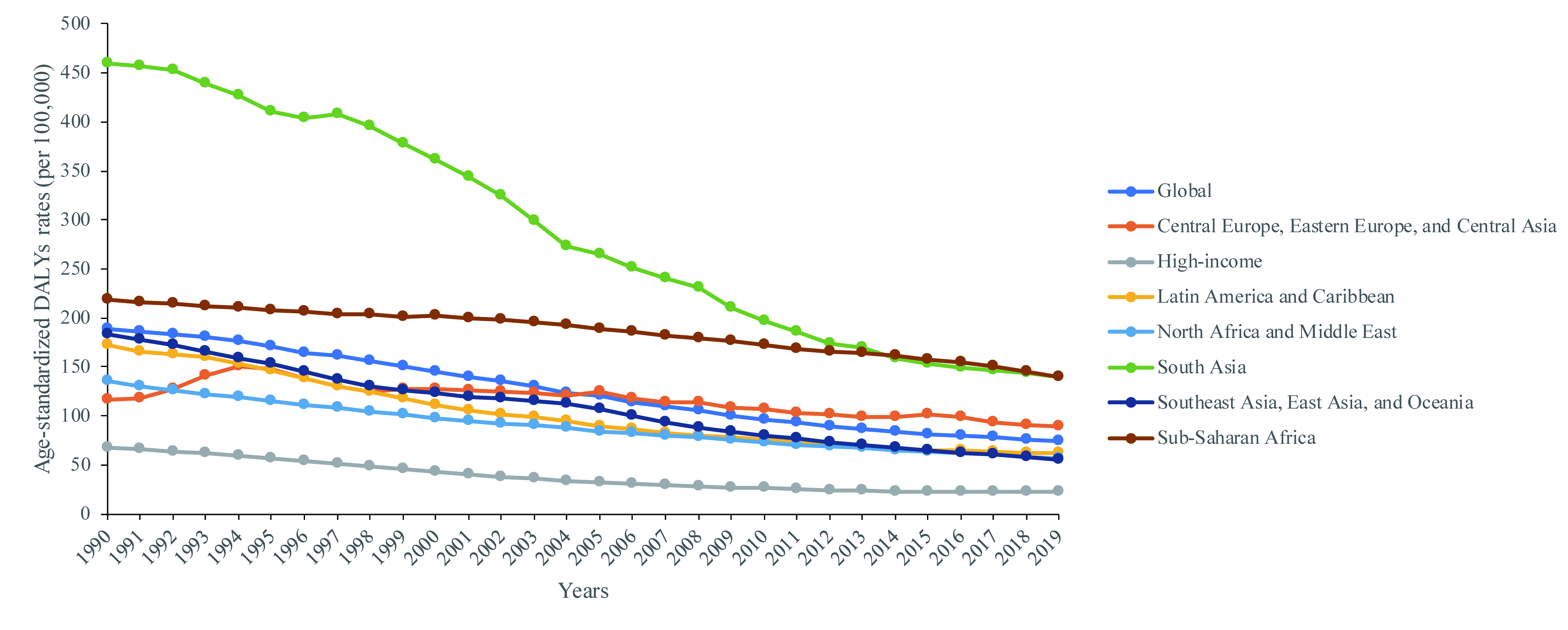


DALYs=disability-adjusted life years.

**Additional file 1:**

**Figure S9. Trends of prevalent cases of PUD in 21 GBD regions in all years from 1990 to 2019**


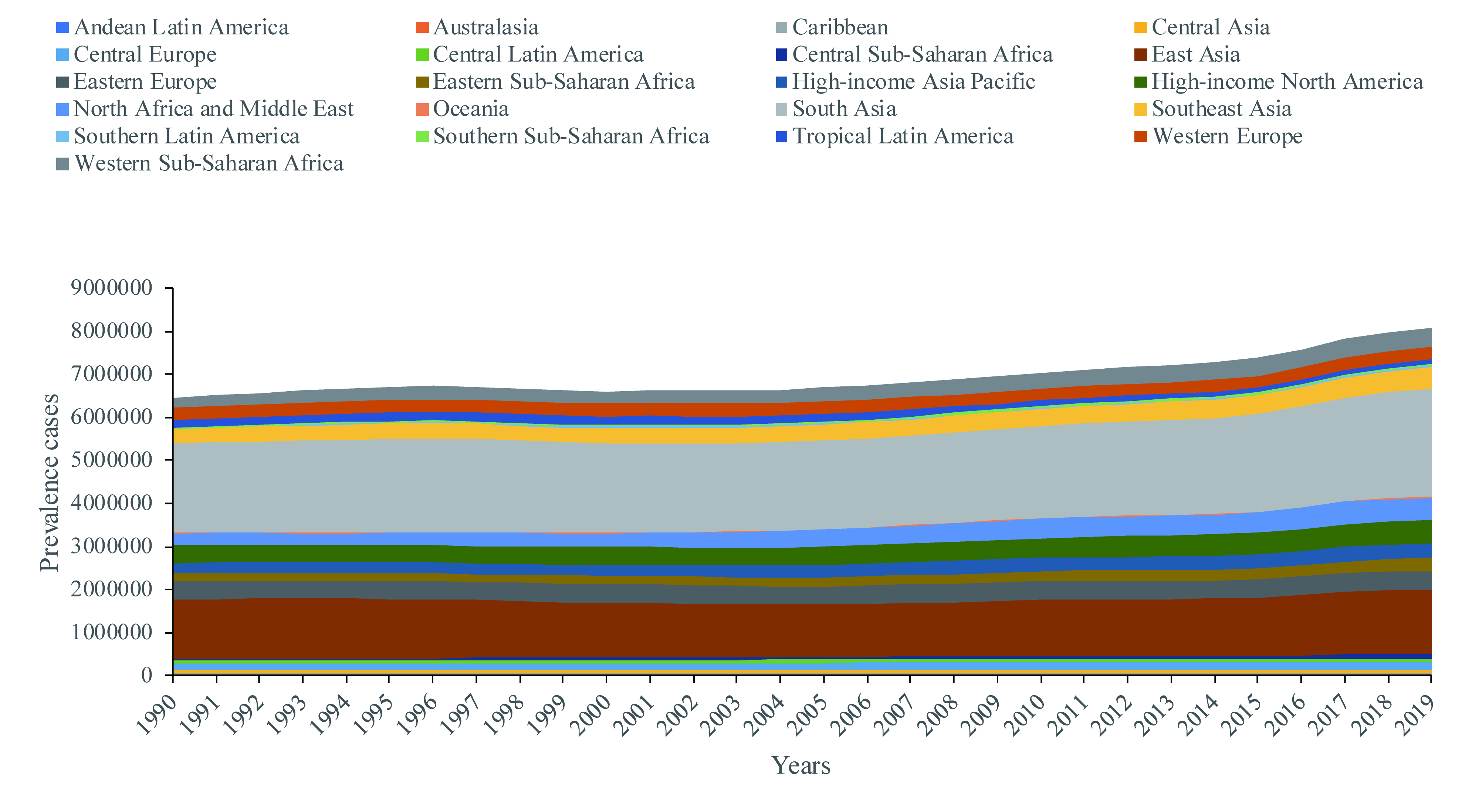


PUD=peptic ulcer disease, GBD=Global Burden of Disease, Injuries and Risk Factors Study.

**Additional file 1:**

**Figure S10. Trends of incident cases of PUD in 21 GBD regions in all years from 1990 to 2019**


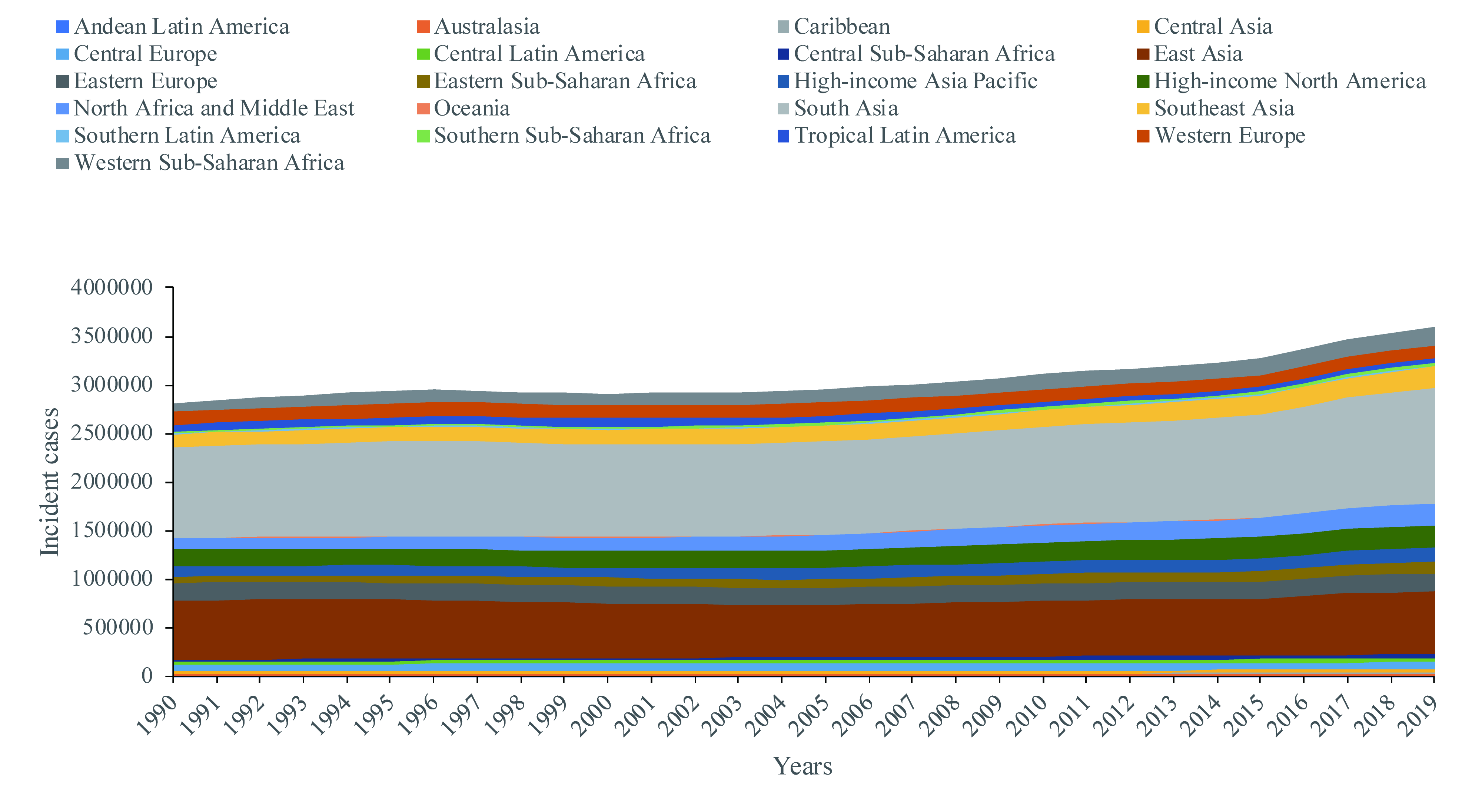


PUD=peptic ulcer disease, GBD=Global Burden of Disease, Injuries and Risk Factors Study.

**Additional file 1:**

**Figure S11. Trends of DALYs due to PUD in 21 GBD regions in all years from 1990 to 2019**


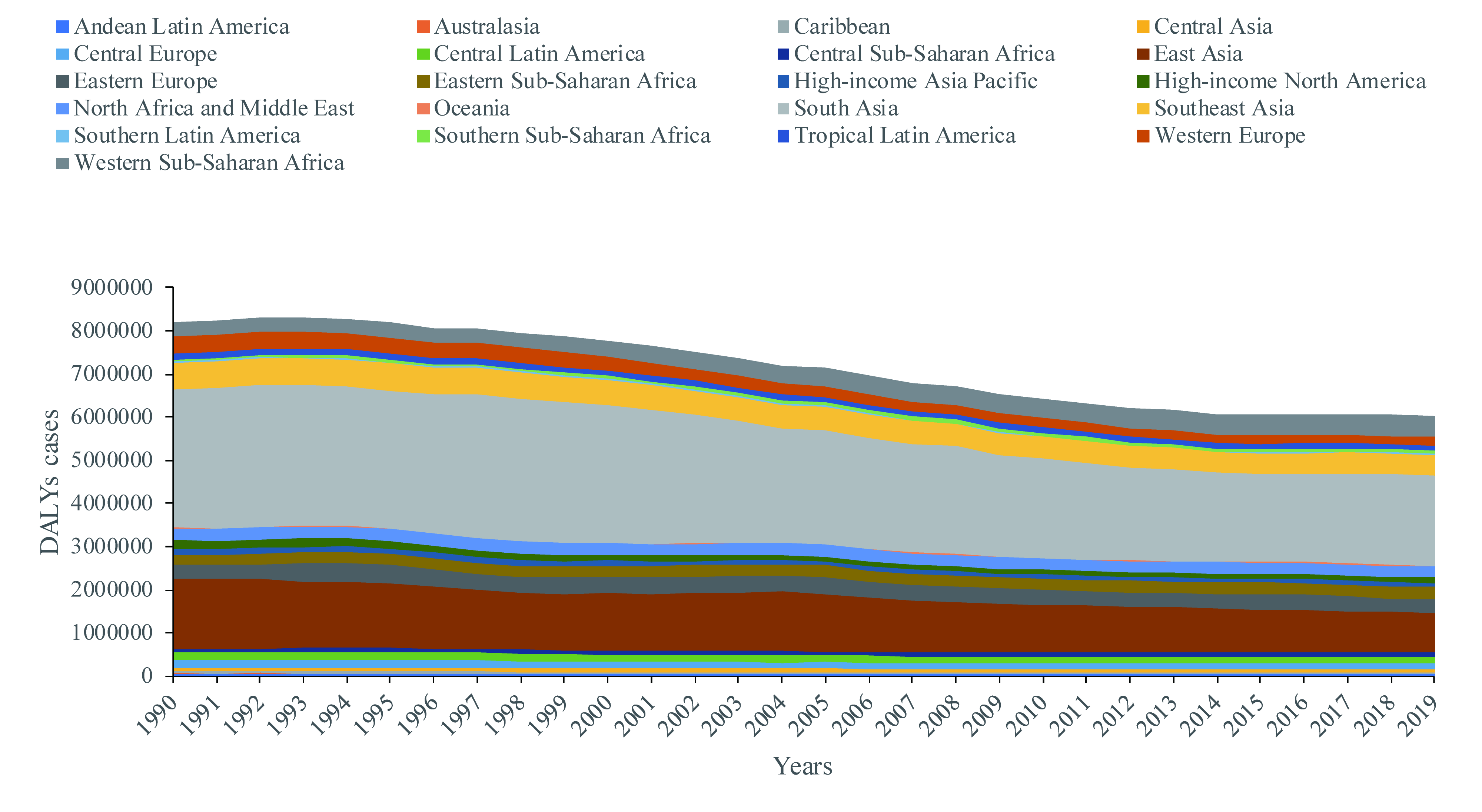


DALYs=disability-adjusted life years, PUD=peptic ulcer disease, GBD=Global Burden of Disease, Injuries and Risk Factors Study.

**Additional file 1:**

**Figure S12. Trends of PUD-related deaths in 21 GBD regions in all years from 1990 to 2019**


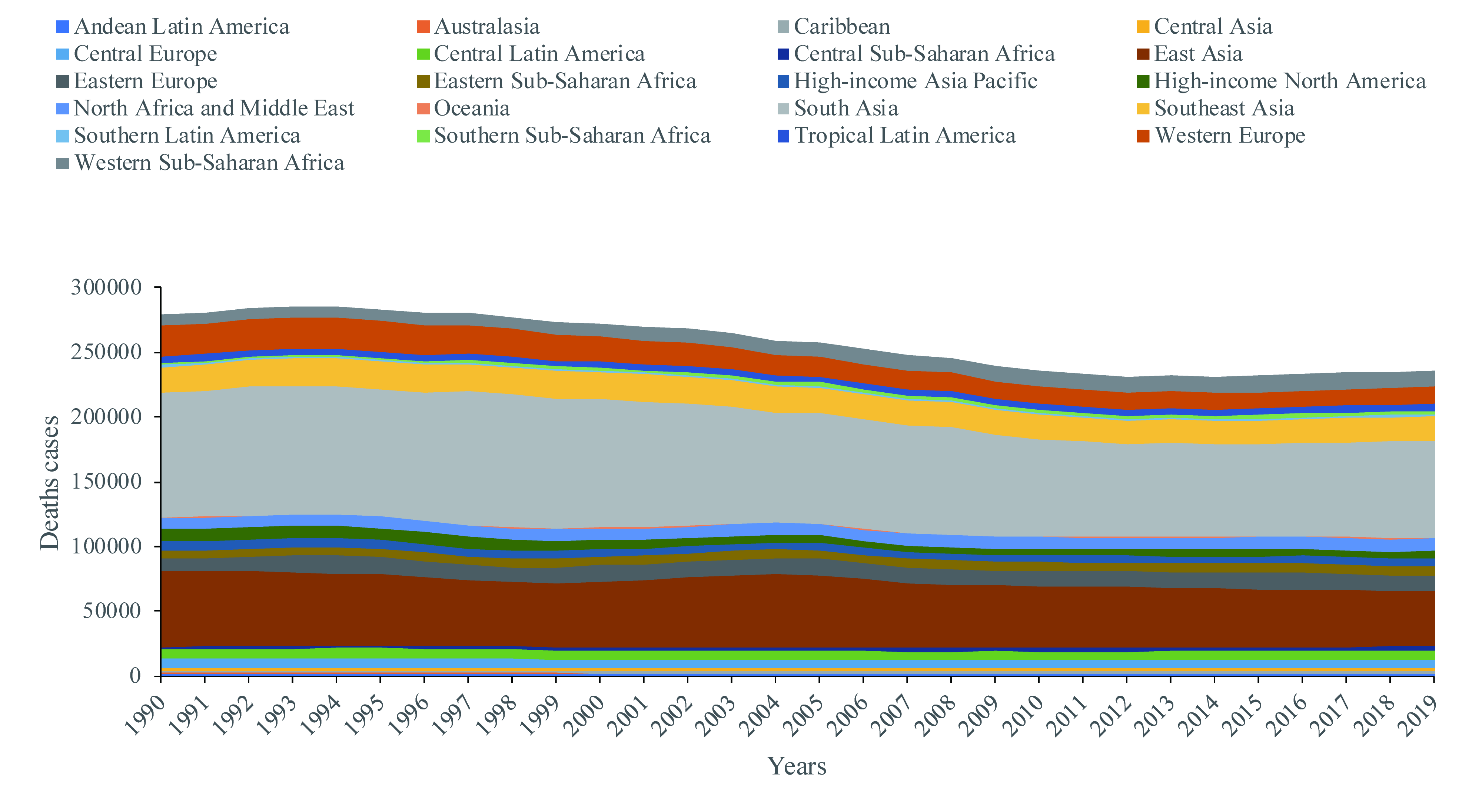


PUD=peptic ulcer disease, GBD=Global Burden of Disease, Injuries and Risk Factors Study.

**Additional file 1:**

**Figure S13. Age-standardized prevalence rates (per 100,000 population) of PUD in males and females in 21 GBD regions in 2019**





PUD=peptic ulcer disease, GBD=Global Burden of Disease, Injuries and Risk Factors Study, UI=uncertainty intervals.

**Additional file 1:**

**Figure S14. Age-standardized incident rates (per 100,000 population) of PUD in males and females in 21 GBD regions in 2019**





PUD=peptic ulcer disease, GBD=Global Burden of Disease, Injuries and Risk Factors Study, UI=uncertainty intervals.

**Additional file 1:**

**Figure S15. Age-standardized DALY rates (per 100,000 population) due to PUD in males and females in 21 GBD regions in 2019**





DALY=disability-adjusted life years, PUD=peptic ulcer disease, GBD=Global Burden of Disease, Injuries and Risk Factors Study, UI=uncertainty intervals.

**Additional file 1:**

**Figure S16. Age-standardized death rates (per 100,000 population) due to PUD in males and females in 21 GBD regions in 2019**





PUD=peptic ulcer disease, GBD=Global Burden of Disease, Injuries and Risk Factors Study, UI=uncertainty intervals.

**Additional file 1:**

**Figure S17. Estimated annual percentage changes in age-standardized prevalent rates in 21 GBD regions between 1990 and 2019**

**
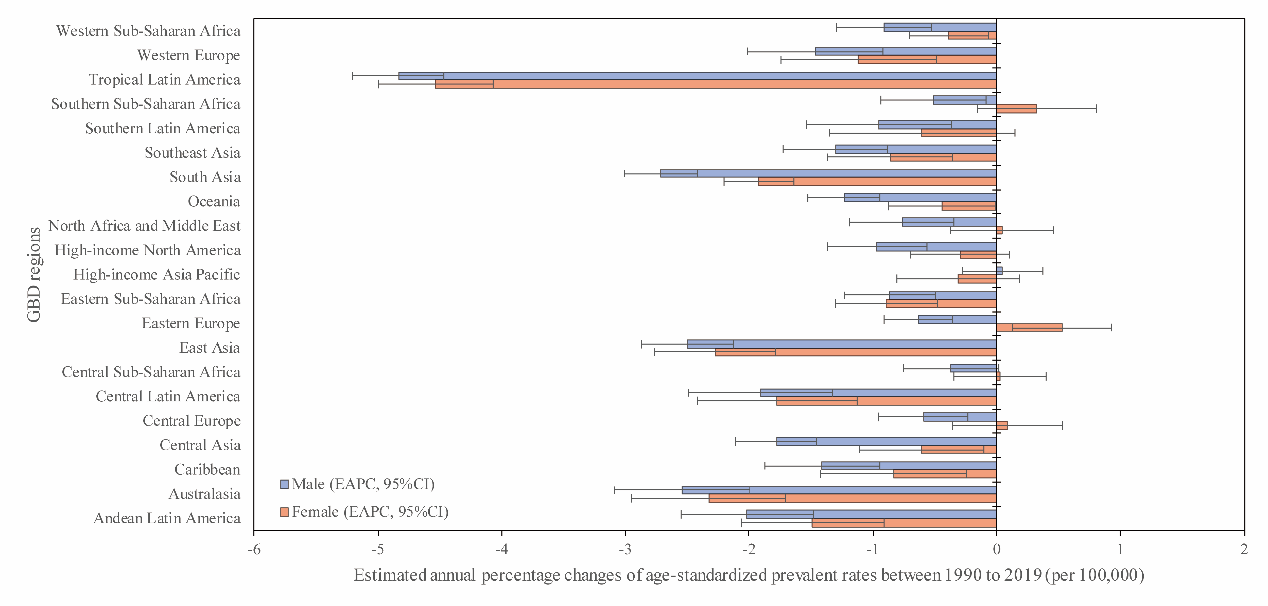
**

GBD=Global Burden of Disease, Injuries and Risk Factors Study, EAPC=estimated annual percentage changes, CI=confidence interval.

**Additional file 1:**

**Figure S18. Estimated annual percentages of age-standardized incident rates (per 100,000 population) in 21 GBD regions between 1990and 2019**





GBD=Global Burden of Disease, Injuries and Risk Factors Study, EAPC=estimated annual percentage changes, CI=confidence interval.

**Additional file 1:**

**Figure S19. Estimated annual percentages of age-standardized DALY rates (per 100,000 population) in 21 GBD regions between 1990and 2019**





DALY=disability-adjusted life years, GBD=Global Burden of Disease, Injuries and Risk Factors Study, EAPC=estimated annual percentage changes, CI=confidence interval.

**Additional file 1:**

**Figure S20. Estimated annual percentages of age-standardized death rates (per 100,000 population) in 21 GBD regions between 1990and 2019**





GBD=Global Burden of Disease, Injuries and Risk Factors Study, EAPC=estimated annual percentage changes, CI=confidence interval.

**Additional file 1:**

**Figure S21. Distributions of age-standardized incidence rates (per 100,000 population) of PUD in different regions in 2019**


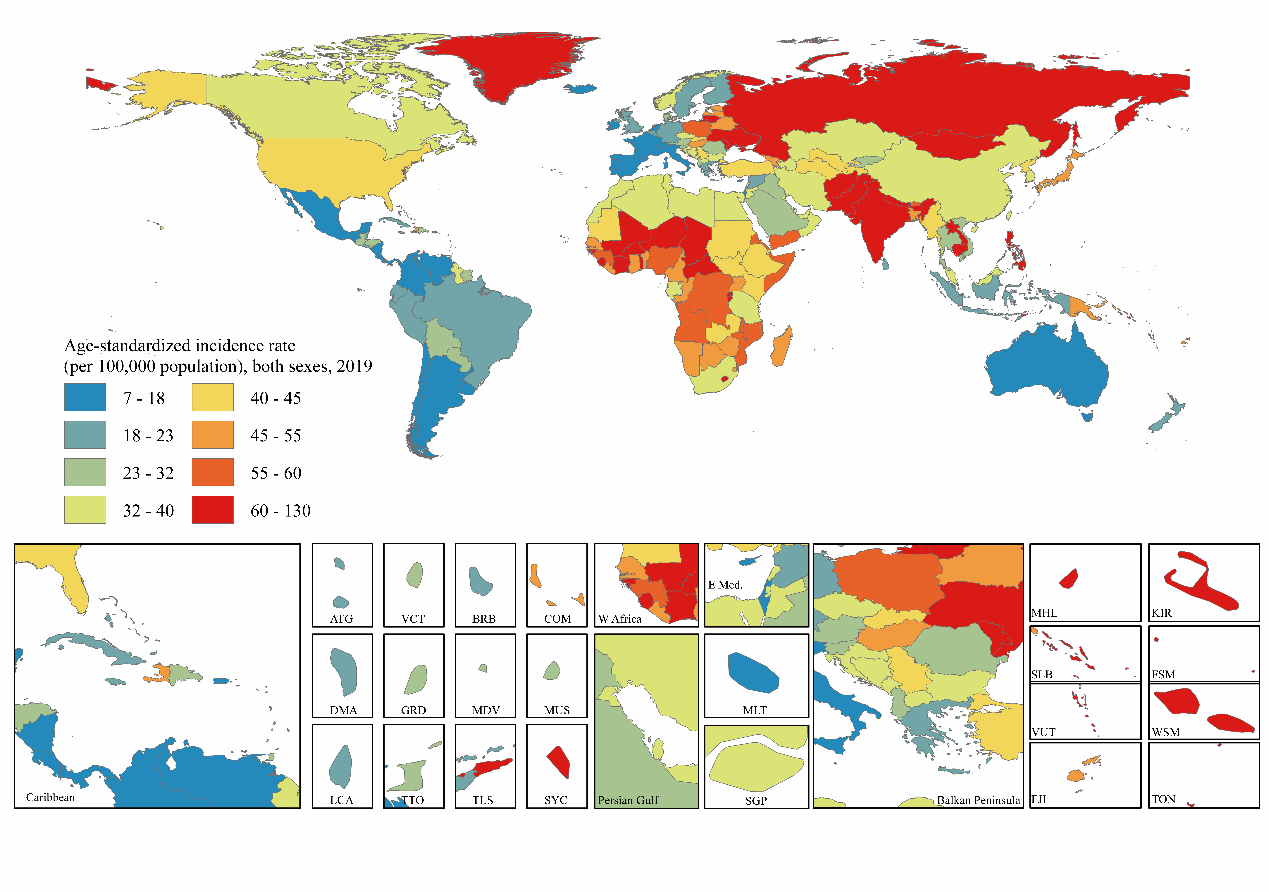


PUD=peptic ulcer disease, ATG=Antigua and Barbuda, BRB=Barbados, COM=Comoros, DMA=Dominica, FJI=Fiji, FSM= Federated States of Micronesia, GRD=Grenada, KIR=Kiribati, LCA=Saint Lucia, MDV=Maldives, MHL=Marshall Islands, MLT=Malta, MUS=Mauritius, SGP=Singapore, SLB=Solomon Islands, SYC=Seychelles, TLS=Timor-Leste, TON=Tonga, TTO=Trinidad and Tobago, VCT=Saint Vincent and the Grenadines, VUT=Vanuatu, WSM=Samoa.

**Additional file 1:**

**Figure S22. Distributions of age-standardized incidence rates (per 100,000 population) of PUD in different regions from 1999 to 2019**


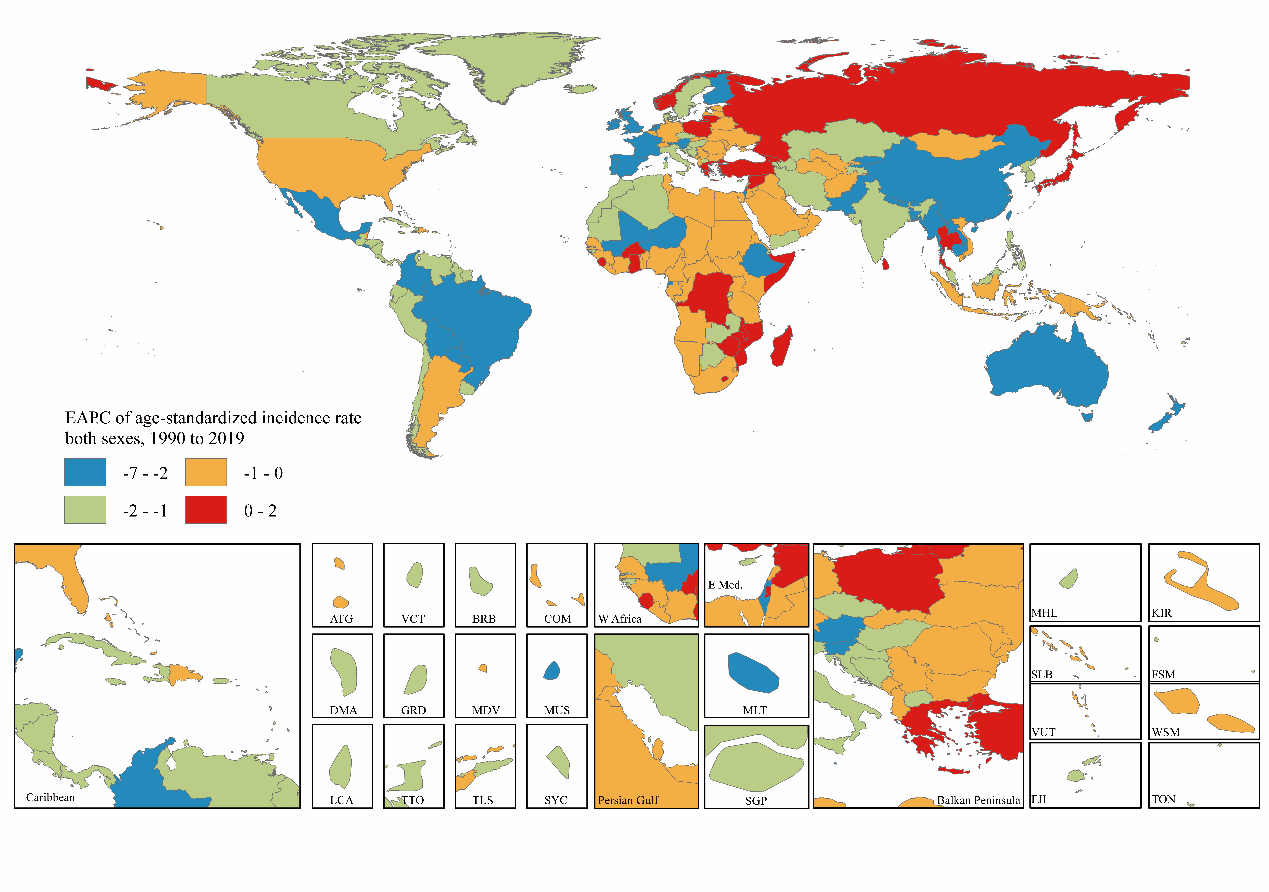


PUD=peptic ulcer disease. ATG=Antigua and Barbuda, BRB=Barbados, COM=Comoros, DMA=Dominica, FJI=Fiji, FSM= Federated States of Micronesia, GRD=Grenada, KIR=Kiribati, LCA=Saint Lucia, MDV=Maldives, MHL=Marshall Islands, MLT=Malta, MUS=Mauritius, SGP=Singapore, SLB=Solomon Islands, SYC=Seychelles, TLS=Timor-Leste, TON=Tonga, TTO=Trinidad and Tobago, VCT=Saint Vincent and the Grenadines, VUT=Vanuatu, WSM=Samoa.

**Additional file 1:**

**Figure S23. Distributions of age-standardized death rates and EAPCs in age-standardized death rates of PUD globally**

**
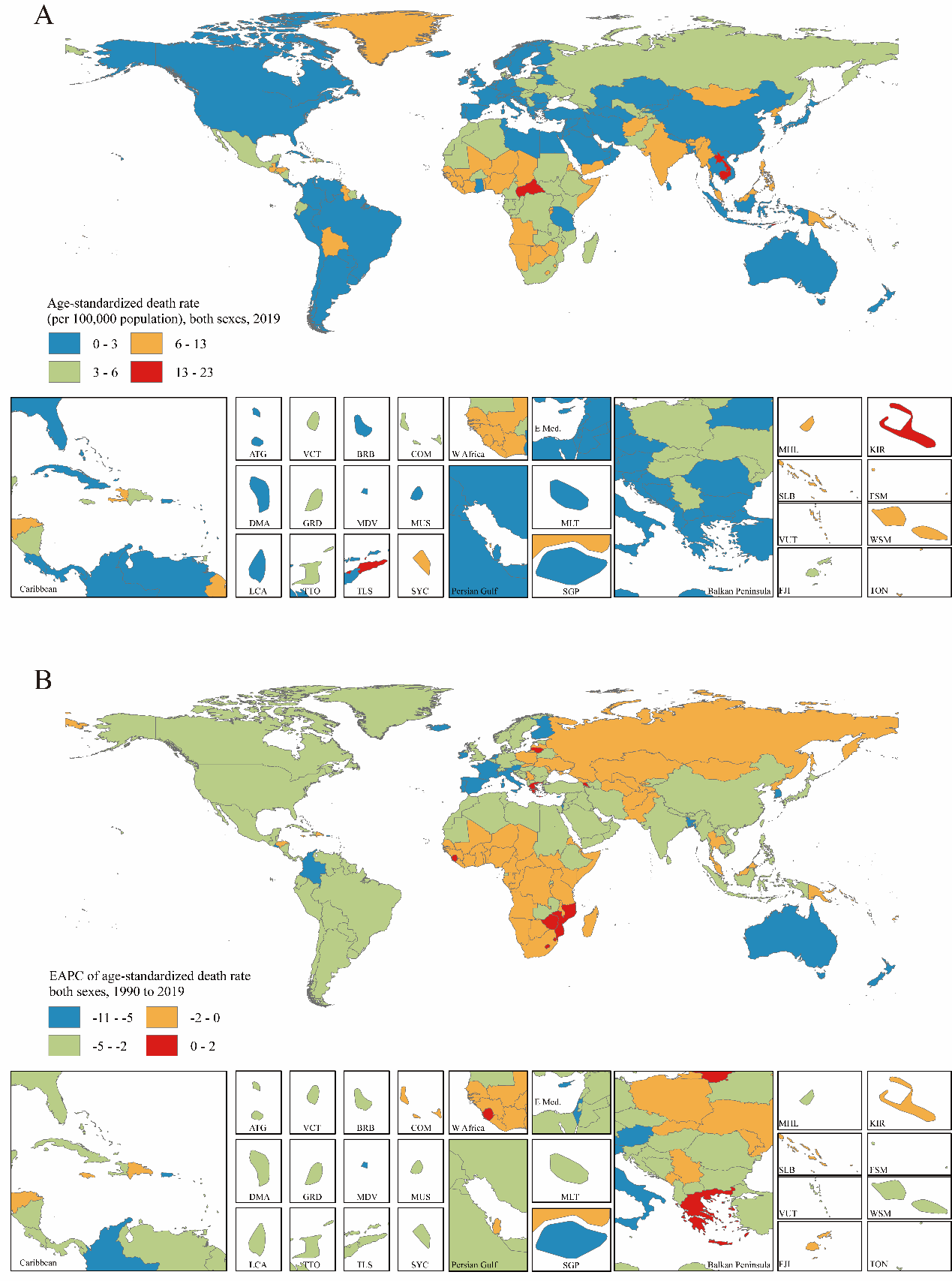
**

(a) The age-standardized death rate (per 100,000 population) in both sexes globally in 2019. (b) The EAPC in the age-standardized death rates in both sexes globally from 1990 to 2019. PUD=peptic ulcer disease, EAPC=estimated annual percentage changes. ATG=Antigua and Barbuda, BRB=Barbados, COM=Comoros, DMA=Dominica, FJI=Fiji, FSM= Federated States of Micronesia, GRD=Grenada, KIR=Kiribati, LCA=Saint Lucia, MDV=Maldives, MHL=Marshall Islands, MLT=Malta, MUS=Mauritius, SGP=Singapore, SLB=Solomon Islands, SYC=Seychelles, TLS=Timor-Leste, TON=Tonga, TTO=Trinidad and Tobago, VCT=Saint Vincent and the Grenadines, VUT=Vanuatu, WSM=Samoa.

**Additional file 1:**

**Figure S24. Trends of age-standardized prevalence rates (per 100,000 population) in different SDI regions from 1990 to 2019**

**
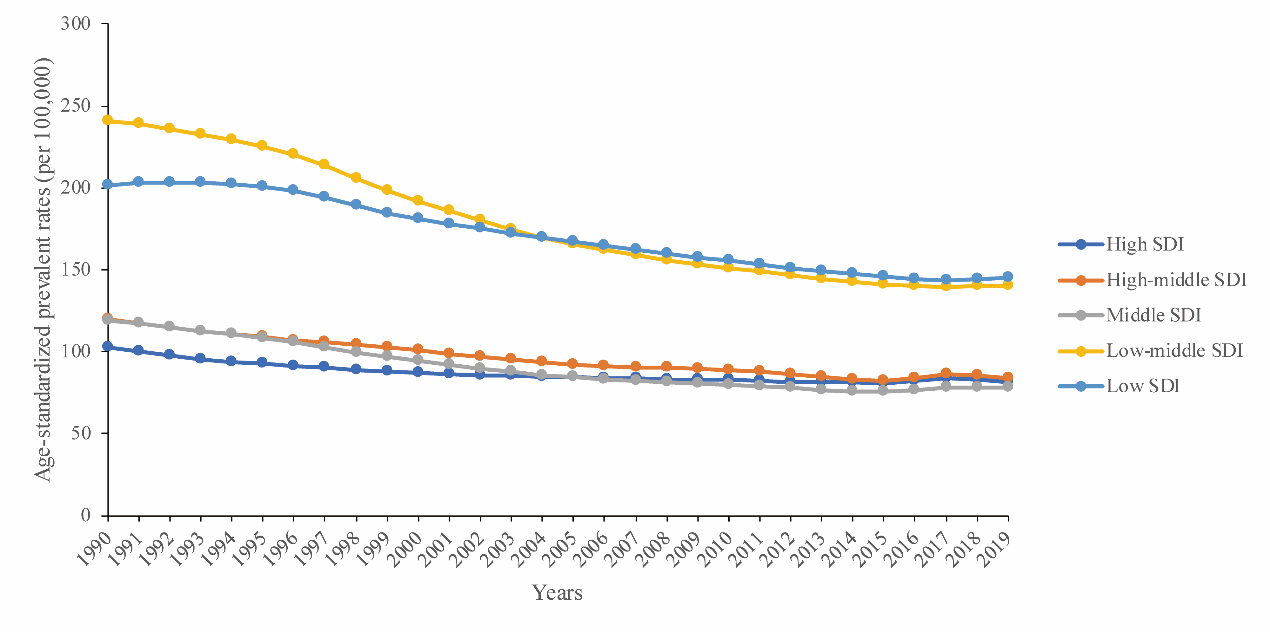
**

SDI=sociodemographic index.

**Additional file 1:**

**Figure S25. Age-standardized death rates (per 100,000 population) from 1990 to 2019 in different SDI regions**


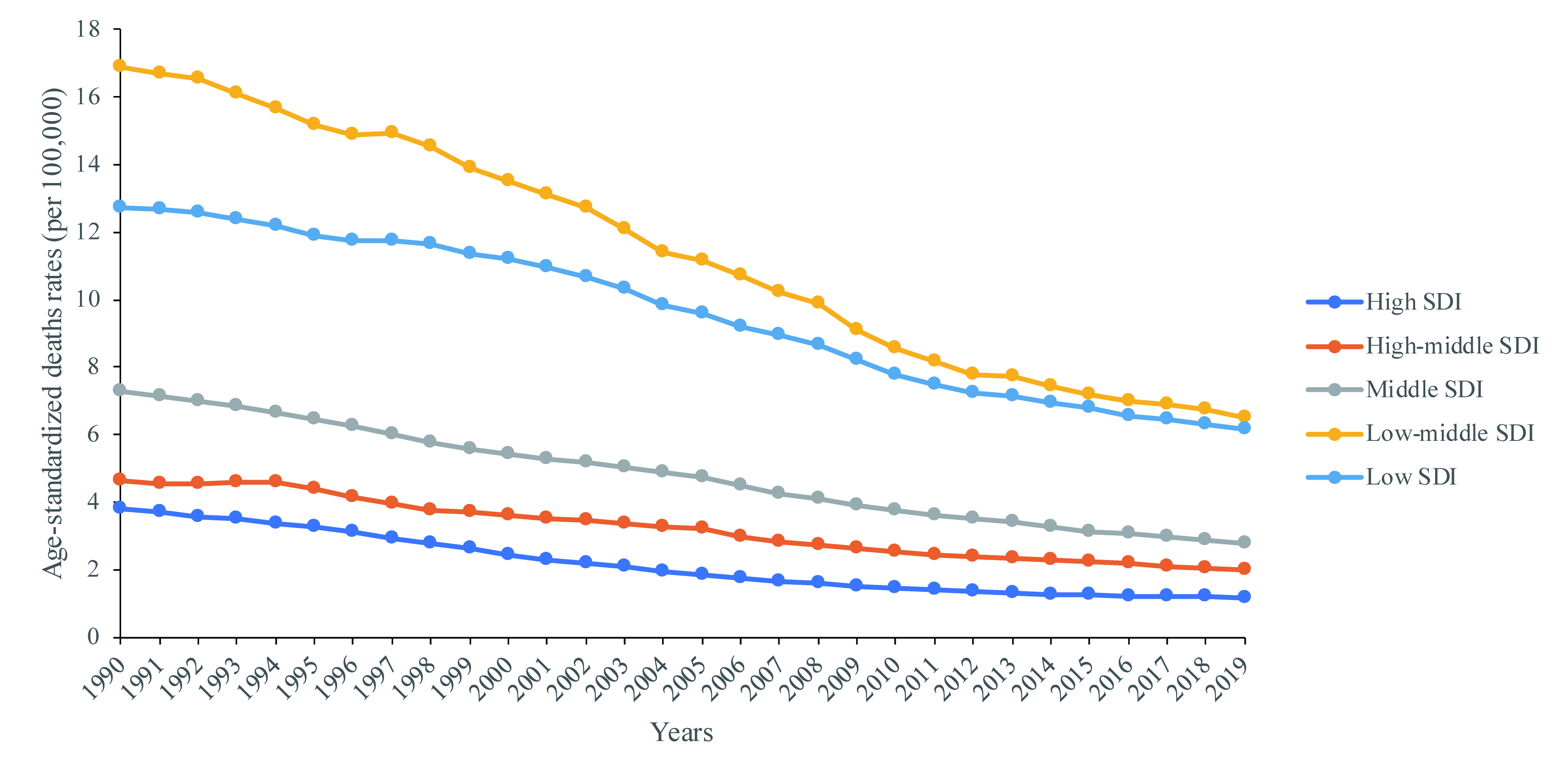


SDI=sociodemographic index.

**Additional file 1:**

**Figure S26. Trends of age-standardized incidence rates (per 100,000 population) of PUD in 21 GBD regions by SDI**


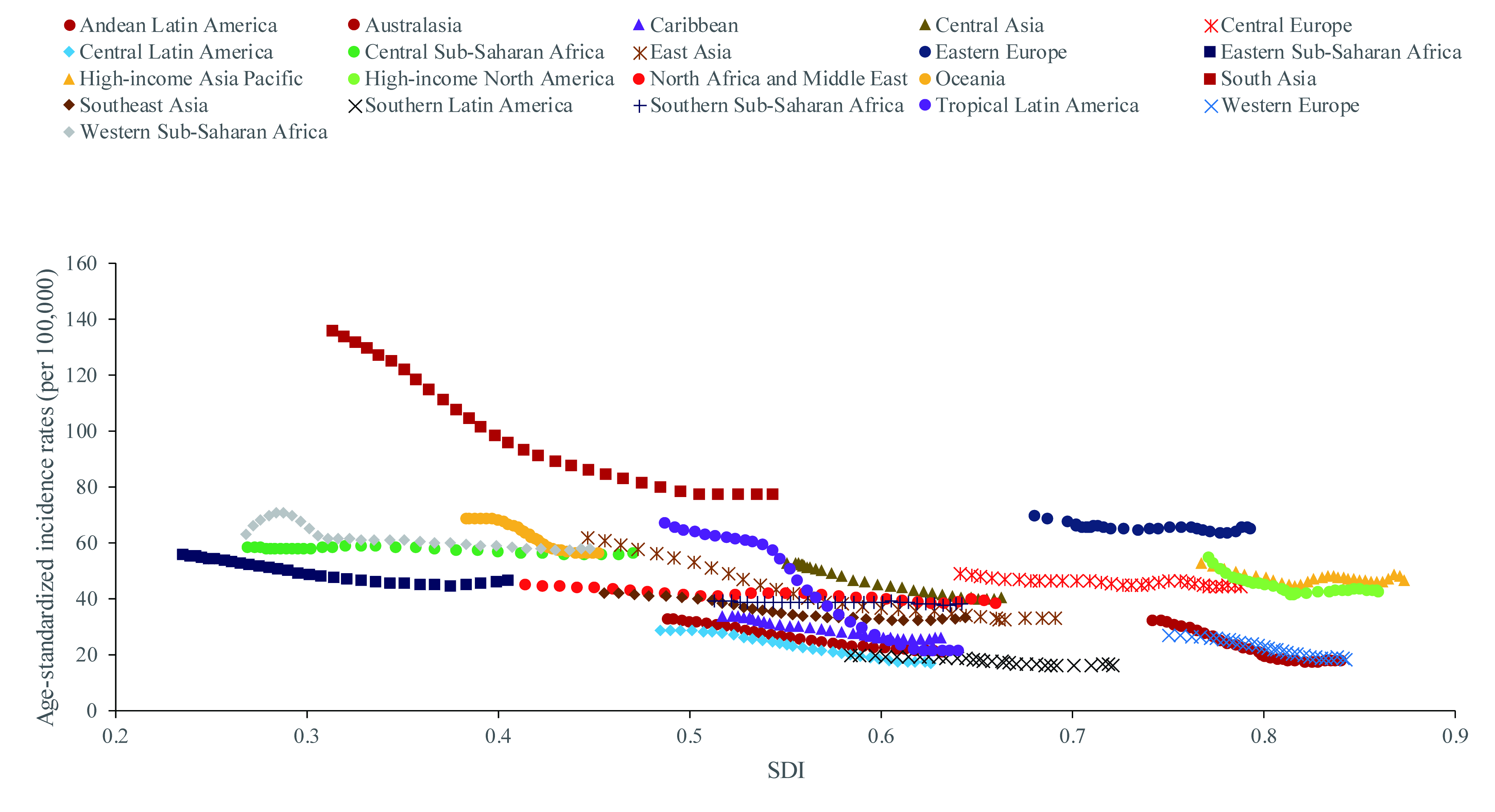


PUD=peptic ulcer disease, GBD=Global Burden of Disease, Injuries and Risk Factors Study(GBD), SDI=sociodemographic index.

**Additional file 1:**

**Figure S27. Trends of age-standardized DALY rates (per 100,000 population) of PUD in 21 GBD regions by SDI**


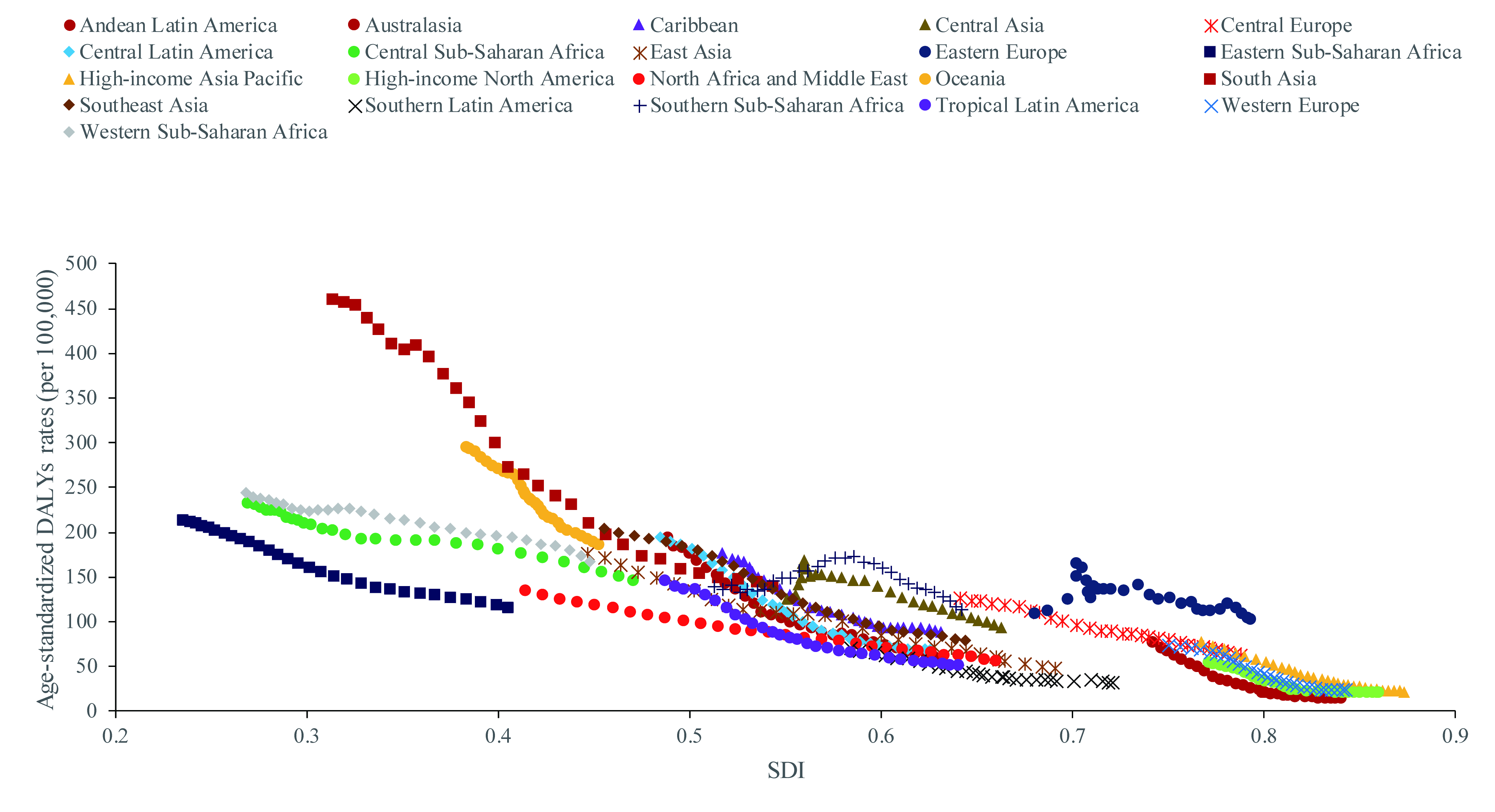


DALY=disability-adjusted life years, PUD=peptic ulcer disease, GBD=Global Burden of Disease, Injuries and Risk Factors Study(GBD), SDI=sociodemographic index.

**Additional file 1:**

**Figure S28. Age-standardized DALY rates (per 100,000 population) due to PUD globally in 204 countries and territories by SDI in 2019**


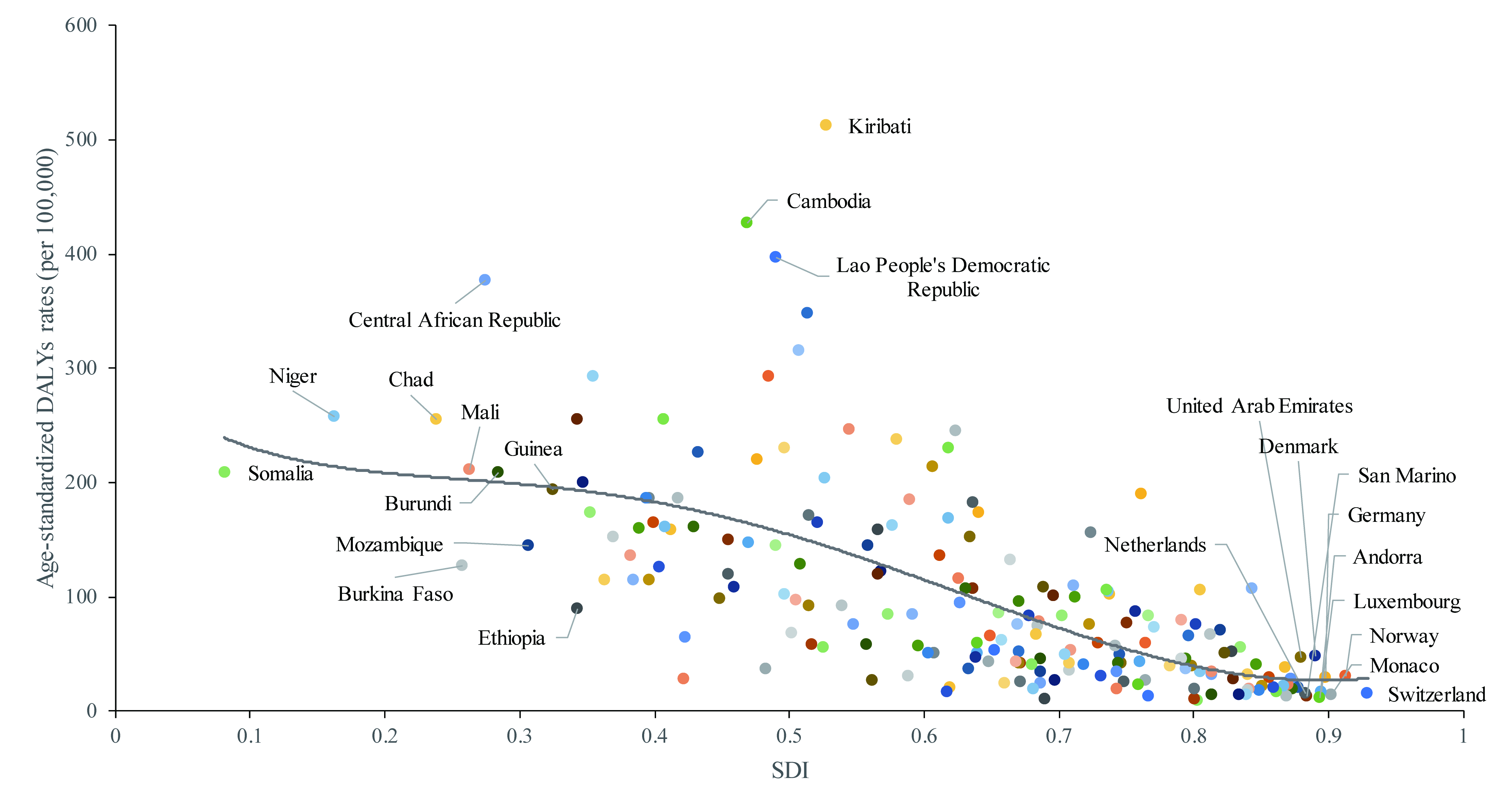


The gray line represents the expected age-standardized DALY rate based on the SDI in 2019. DALY=disability-adjusted life years, PUD=peptic ulcer disease, SDI=sociodemographic index.
